# Heavy Alkali Metal Manganate Complexes: Synthesis, Structures and Solvent‐Induced Dissociation Effects

**DOI:** 10.1002/chem.202201716

**Published:** 2022-08-03

**Authors:** Gerd M. Ballmann, Thomas X. Gentner, Alan R. Kennedy, Eva Hevia, Robert E. Mulvey

**Affiliations:** ^1^ WestCHEM Department of Pure and Applied Chemistry University of Strathclyde Glasgow G1 1XL UK; ^2^ Department für Chemie und Biochemie Universität Bern Freiestrasse 3 3012 Bern Switzerland

**Keywords:** alkali metals, amides, ligand redistribution, manganese, structural elucidation

## Abstract

Rare examples of heavier alkali metal manganates [{(AM)Mn(CH_2_SiMe_3_)(N^‘Ar^)_2_}_∞_] (AM=K, Rb, or Cs) [N^‘Ar^=N(SiMe_3_)(Dipp), where Dipp=2,6‐*i*Pr_2_‐C_6_H_3_] have been synthesised with the Rb and Cs examples crystallographically characterised. These heaviest manganates crystallise as polymeric zig‐zag chains propagated by AM⋅⋅⋅π‐arene interactions. Key to their preparation is to avoid Lewis base donor solvents. In contrast, using multidentate nitrogen donors encourages ligand scrambling leading to redistribution of these bimetallic manganate compounds into their corresponding homometallic species as witnessed for the complete Li ‐ Cs series. Adding to the few known crystallographically characterised unsolvated and solvated rubidium and caesium s‐block metal amides, six new derivatives ([{AM(N^‘Ar^)}_∞_], [{AM(N^‘Ar^)⋅TMEDA}_∞_], and [{AM(N^‘Ar^)⋅PMDETA}_∞_] where AM=Rb or Cs) have been structurally authenticated. Utilising monodentate diethyl ether as a donor, it was also possible to isolate and crystallographically characterise sodium manganate [(Et_2_O)_2_Na(^*n*^Bu)Mn[(N^‘Ar^)_2_], a monomeric, dinuclear structure prevented from aggregating by two blocking ether ligands bound to sodium.

## Graphical abstract

**Heavy alkali metal** alkyls can form ate compounds with manganese bis(amides) when donor solvents are absent but introducing the common donors TMEDA or PMDETA leads to breakdown of the ates together with ligand exchange, which results in the formation of new solvated heavier alkali metal amides and alkylmanganese amides.

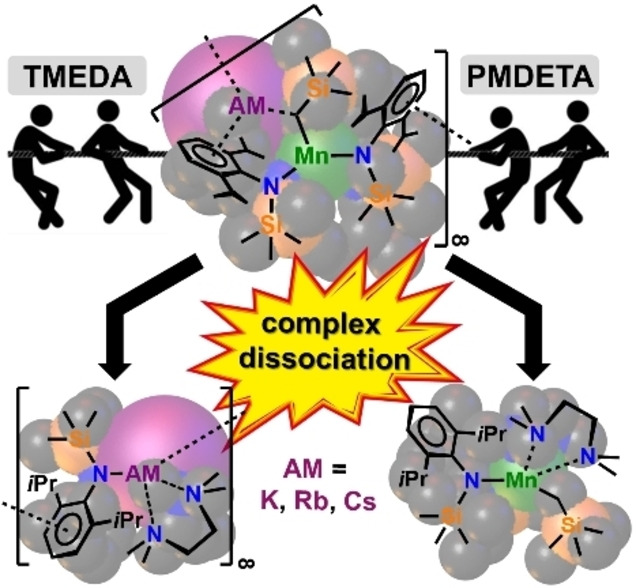

## Introduction

Manganese occupies a unique position in the centre of the periodic table as it exists in oxidation states ranging from −3 to +7,^[1]^ indicating a most versatile reactivity in reactions with organic substrates. The oxidation state +2 illustrates its uniqueness in particular, distinguishing it from its transition metal neighbours by significant ionic contribution of the metal‐carbon bond. This has been attributed to the significant harder character of Mn(II) when compared to other first row transition metal ions.^[2]^ This “main‐group‐mimic” characteristic in combination with accessible redox‐chemistry and the fact that manganese is i) earth abundant ii) non‐toxic and iii) inexpensive compared to other transition metals[^3^, ^4^] has led to a wide range of applications of organometallic Mn(II) compounds, as for example in acylation, addition and other C−C bond forming or oxidation reactions.[^5^, ^6^]

The first applications of organomanganese(II) rest on the pioneering reports by Normant and Cahiez in the 1970s.^[7]^ Subsequently, four main organomanganese(II) reagent types have received most attention: namely, organomanganese halides, RMnX;^[8]^ diorganyl manganese compounds MnR_2_;[^9^, ^10^, ^11^] lower‐order triorganomanganates [MnR_3_]^−^,^[12]^ and higher order tetraorganomanganates [MnR_4_]^2−^.[^11^, ^12^, ^13^] The third and fourth types require cations for charge balance. In general, the lower‐ and higher‐order ate compounds benefit from increased thermal stability and solubility compared to the monometallic analogues, hence the chemistry of polyalkyl‐manganates has been extensively studied and reviewed by Cahiez,^[14]^ Duplais and Buendia,^[7]^ and by Oshima.^[15]^ Despite their interesting organic applications, the identity of these species has remained largely concealed, although recent studies by Uzelac and Hevia have shown the applications of structurally‐defined lithium manganate [(TMEDA)_2_Li_2_Mn(CH_2_SiMe_3_)_4_] to promote oxidative homocoupling of aryliodides.^[16]^

An interesting feature is the unique synergic reactivity exhibited by certain heterobimetallic or mixed metal (ate) compounds that occurs upon mixing Mn(II)[^17^, ^18^, ^19^] or other divalent metal complexes (e. g., Mg[^20^, ^21^, ^22^, ^23^, ^24^, ^25^, ^26^] or Zn[^23^, ^24^, ^27^, ^28^, ^29^]) with alkali metal precursors AM−R (with AM=usually Li, Na, or K, but rarely Rb and Cs).[^22^, ^30^] The synergistic reactivity seen with alkali metal manganates (notably monoalkyl‐bisamido manganates) has been labelled alkali‐metal‐mediated *manganation* (AMM*Mn*),[^18^, ^19^, ^31^] in respect to metallation (C−H to C‐metal exchange) reactions, which fail to work at all or to work efficiently without the intervention of an alkali metal.

Though not studied in much detail, AMM*Mn* bears a close similarity to the more extensively studied alkali‐metal mediated *magnesiations* (AMM*Mg*) due to shared features between Mn(II) and Mg(II). It is therefore not surprising that analogous Mg(II) complexes (or *vice versa*) can be found in the literature for a large number of Mn(II) structures. Prominent examples include Power's mixed lithium‐manganate trisamide complex [Mn{N(SiMe_3_)_2_}_3_Li(THF)]^[32]^ and isostructural lithium‐magnesiate trisamide complex [Mg{N(SiMe_3_)_2_}_3_Li(THF)]^[33]^ or the hydride encapsulated inverse crown sodium‐magnesiate [Na_2_Mg_2_(μ‐H)_2_{N(*i*Pr)_2_}_4_(toluene)_2_]^[34]^ and its manganese(II) analogue [Na_2_Mn_2_(μ‐H)_2_{N(*i*Pr)_2_}_4_(toluene)_2_]^[35]^ reported by Mulvey. Recently, as part of a growing movement to develop the use of main group compounds in catalysis, chemists have begun to study magnesiate compounds in homogeneous catalysis.[^36^, ^37^, ^38^] Interestingly, heavier alkali metals, nearly always potassium, but also to a much lesser extent, rubidium and caesium, have played key roles in this development.^[39]^ In this context Guan and co‐workers have successfully utilised mixtures of saline potassium hydride KH and group 2 metal bis‐hexamethyldisilazides M(HMDS)_2_ [M=Mg, Ca; HMDS=N(SiMe_3_)_2_] that form effective catalysts for hydrogenation of olefins.^[40]^ The active species in these transformations in the case of magnesium (M=Mg) is evidenced to be the mixed amido‐/hydrido‐ potassium magnesiate [KMg(H)(HMDS)_2_]_2_, which was first reported and structurally characterised by Hill.^[41]^

We would advocate that it is now considered vital to include the whole of group 1 (Li ‐ Cs) when investigating catalytic performance, since gradations in reactivity can take place depending on which alkali metal is used. Investigations to gain more structural insights in whole series of group 1 complexes often exclude the heavier homologues due to the synthetic challenge or inaccessibility,[^42^, ^43^] hence studies such as that by Schulz,^[44]^ Roesky,^[45]^ or Liddle,^[46]^ in which light is shed on the whole of group 1 within the same ligand framework, remain exceptional. One of the most widely utilized ligands to study alkali metals in complexes are amide derivatives since they are of central importance in many chemical transformations as metallation and amide‐transfer reagents or as Brønsted bases.[^47^, ^48^, ^49^] Again, the amide chemistry of the heaviest group 1 elements rubidium and caesium is less developed with structurally characterised compounds being restricted to few examples of i) silyl‐stabilised (trimethylsilyl)‐ or (phenyl‐dimethylsilyl) amides,[^50^, ^51^, ^52^, ^53^, ^54^, ^55^, ^56^, ^57^, ^58^, ^59^] ii) electron withdrawing perfluorinated alkyl‐ and aryl‐substituted amides^[60]^ or iii) sterically hindered 2,2,6,6‐tetramethylpiperidides.^[61]^ Silyl‐stabilised bulky amides, especially silyl‐aryl‐substituted derivatives, may offer a lower degree of aggregation and enable solubility in common organic solvents. This is particularly important for the heavier alkali metal amides as the increasingly electrostatic metal nitrogen bond favours the formation of insoluble salt like compounds. The possibility of the alkali metal to engage in inter‐ and intramolecular anagostic as well as AM⋅⋅⋅π‐arene interactions with the ligand framework can be the decisive factor in the formation of fundamentally different complex structures as highlighted in recent advances in low‐valent main group metal chemistry.[^62^, ^63^]

Investigation and deeper insight into the class of Mn(II) compounds remains challenging, since stringent exclusion of air to prevent unwanted oxidation to Mn(IV) species is paramount, while characterisation by NMR spectroscopy is hindered by the paramagnetism of Mn(II). Additionally, isolation and handling of the mediating alkali metal alkyl precursor is impeded with increasing electropositivity of the group 1 metal (Cs>Rb>K>Na>Li), as has recently been shown in the challenging structural elucidation of the heaviest alkali‐metal‐benzyl complexes by Strohmann.^[64]^ Hence, manganate(II) compounds bearing the heaviest alkali metals are scarce and only combinations of rubidium with manganese in mixed or higher oxidation states,^[65]^ in oxidation state +1^[66]^ or in hydrophilic coordination compounds^[67]^ and metal–organic frameworks^[68]^ (MOFs) are structurally identified. Similarly, while Cs^+^ is commonly found as a counter‐cation in water soluble coordination compounds or caged in cluster complexes containing manganese in the oxidation state +2 or +3,^[69]^ only one study from Berke reveals structural insight of air‐ and moisture‐sensitive organocaesium compounds in the tris(cyclopentadienyl) manganates.^[70]^

With this background, this study sets out to merge these different aspects by attempting to synthesise and characterise a series of new alkali metal manganates, with particular emphasis on rubidium and caesium examples. First, we highlight the influence of the coordinating donor ligands, THF, Et_2_O, TMEDA (*N,N,N’,N’*‐tetramethylethylenediamine) and PMDETA (*N,N,N’,N”,N”*‐pentamethyldiethylenetriamine) in the success or failure to isolate challenging manganate‐complexes instead of a mixture of their heteroleptic, monometallic alkyl‐amido Mn(II) compounds and alkali metal amides, derived from manganate‐complex destruction. The heaviest homologues of these alkali metal amides are also discussed extensively in terms of their structural diversity when solvated with said donors. We also reveal how exclusion of donor solvents allowed the successful synthesis and structural characterisation of the, to the best of our knowledge, first Rb‐manganate with manganese solely in the oxidation state +2 and its heavier Cs‐homologue prepared in hydrocarbon solvents.

## Results and Discussion

### Initial attempts to prepare alkali metal manganate complexes

**Scheme 1:**
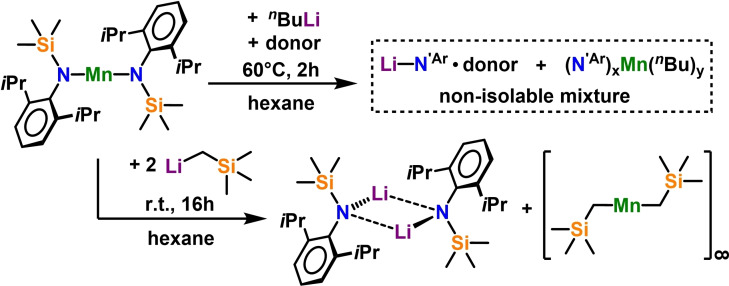
Reaction of Mn(N‘Ar)_2_ with lithium‐alkyls: formation of mixture of organomanganese compounds in presence of donor solvents (top) and two‐fold ligand redistribution in aliphatic solvents (bottom).

We started our investigations by mixing stoichiometric amounts of the literature known bis‐arylsilyl amide Mn(N^‘Ar^)_2_ ^[71]^ [N^‘Ar^=N(SiMe_3_)(Dipp), where Dipp=2,6‐*i*Pr_2_‐C_6_H_3_] and commercially available *n*‐butyl lithium solution in hexane. Unfortunately, these co‐complexation attempts, that is the formal addition of two monometallic species to yield a new bimetallic complex, repeatedly resulted in the formation of mixtures of lithium amide LiN^‘Ar^ or its monometallic coordination compounds with various donor solvents and yellowish residue, which failed to deposit any crystalline material for further investigation (see Scheme 1).

Changing the lithium alkyl precursor to the neosilyl lithium LiCH_2_SiMe_3_ reinforces this observation as, in separate experiments, crystals of homometallic LiN^‘Ar^ ⋅ (Et_2_O)_2_,^[72]^ LiN^‘Ar^ ⋅ (THF)_3_,^[73]^ LiN^‘Ar^ ⋅ (TMEDA)^[74]^ and LiN^‘Ar^ ⋅ (PMDETA)^[74]^ could be identified upon repeating the reaction with addition of Et_2_O, THF, TMEDA and PMDETA respectively, consistent at least in part with manganese‐lithium exchange phenomena. The total absence of additional donor solvent promoted the formation of formal twofold ligand exchange to yield one equivalent of the [LiN^‘Ar^]_2_ ^[75]^ dimer with one equivalent of polymeric [{Mn(CH_2_SiMe_3_)_2_}_∞_],[^12^, ^76^] which can be evidenced by X‐ray analysis and is observed by a colour change to the distinctive orange colour of [{Mn(CH_2_SiMe_3_)_2_}_∞_].

**Scheme 2:**
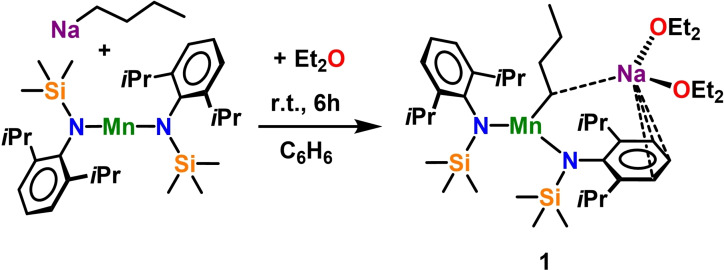
Synthesis of sodium manganate [(Et_2_O)_2_Na(^*n*^Bu)Mn[(N^‘Ar^)_2_] (**1**).

As this ligand redistribution seemingly follows Pearson's acid‐base concept with the hard Li^+^ cation preferring the harder nitrogen of the amide ligand, we next investigated the influence of heavier alkali metals on this metathesis reaction with respect to the donor solvents applied. Treatment of Mn(N^‘Ar^)_2_ dissolved in benzene with freshly prepared ^*n*^BuNa under reflux conditions leads to precipitation of a white solid of unknown composition. Attempts to recrystallize this material in aromatic solvents failed, but slow diffusion of *n*‐hexane into a concentrated Et_2_O solution yielded colourless crystals in 71 % yield and suitable for X‐ray crystallographic determination, revealing the bimetallic sodium manganate [(Et_2_O)_2_Na(^*n*^Bu)Mn[(N^‘Ar^)_2_] (**1**) (see Scheme 2).

**Figure 1:**
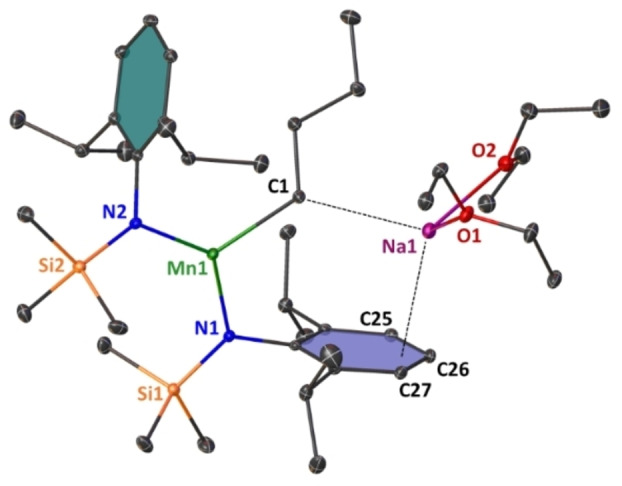
Molecular structure of monomeric, bimetallic [(Et_2_O)_2_Na(^*n*^Bu)Mn[(N^‘Ar^)_2_] **(1)**. Ellipsoids are displayed at 30 % probability and hydrogen atoms are omitted for clarity. Anagostic interaction and π‐arene interactions are shown as dashed lines. Ph rings are coloured for better clarity. Selected bond lengths [Å] and angles [°]: Mn1‐N1 2.0586(10); Mn1‐N2 2.046(1); Mn1‐C1 2.1668(13); Na1‐C1 2.8363(14); Na1‐O1 2.3563(10); Na1‐O2 2.4155(11); Na1‐C25 3.1190(14); Na1‐C26 2.8122(14); Na1‐C27 3.0180(13); N1‐Mn1‐N2 132.33(4); N2‐Mn1‐C1 121.64(5); C1‐Mn1‐N1 106.04(5).

Compound **1** was characterised by combining X‐ray crystallographic studies (see Figure 1) with elemental analysis and infrared spectroscopy (see Supporting Information for details). Attempts to record meaningful NMR spectra in benzene‐*d_6_* and THF‐*d_8_* solutions were unsuccessful due to the paramagnetic nature of Mn(II). However, Evans methods could be used to assess its magnetic moment (effective magnetic moment μ_eff_=5.94 μ_B_) which is consistent with five unpaired electrons in a high‐spin state for the Mn(II) centre. Manganate **1** crystallised in the monoclinic primitive space group *P*2_1_/*c* and can be classified as a contacted ion pair type complex, containing a 2 : 1 amido/butyl stoichiometric ratio, similar to that of previously reported Mn(II) synergistic metalating agents.[^19^, ^31^] In comparison to these systems, the arrangement of the amide ligand in **1** is strikingly different though, as none of the amide ligands lie in a bridging position between the Mn and Na metal but solely bind to the transition metal. Thus, Mn1 occupies a N_2_C trigonal planar coordination (sum of bond angles: 360.0°) comprising two amide ligands (mean Mn−N distance, 2.0523 Å) and one butyl ligand [Mn1‐C1, 2.1668(13) Å]. This three‐coordinate Mn moiety is connected through two bridges to the Na1 centre, which overcomes intermolecular bonding tendencies by coordination to two diethyl ether molecules. One bridge consists of the α‐carbon of the bridging butyl ligand and the other is the η^3^‐coordination (Na1‐C25: 3.1190(14) Å, Na1‐C26: 2.8122(14) Å, Na1‐C27: 3.0180(13) Å) to one of the amido ligand's Dipp groups. These alkali metal‐arene interactions are decisive for the formation of the polymeric helical chain structure in the related magnesium compound [{(Na)Mg(^*n*^Bu)(N^‘Ar^)_2_}_∞_]^[74]^ and are a common feature in main group metal chemistry in general, thus they have been discussed extensively in the literature.[^77^, ^78^, ^79^]

It should be noted that adding the more strongly donating oxygen donor solvent THF did not allow for isolation of a defined species. This has been the incentive to investigate for possibly complex formation tendencies with multidentate donor ligands.

Switching to the tridentate nitrogen donor PMDETA led to complex dissociation with the isolation of single metal Na(N^‘Ar^)⋅PMDETA. While the Mn counterpart could not be isolated, it can be assumed that Mn(N^‘Ar^)(^*n*^Bu)⋅PMDETA was also formed, though this species appears to be highly soluble in hydrocarbon solvents and attempts to isolate it as a crystalline solid were unsuccessful. This donor‐mediated dissociation process has already been observed in related magnesiate compounds.^[74]^

**Scheme 3:**
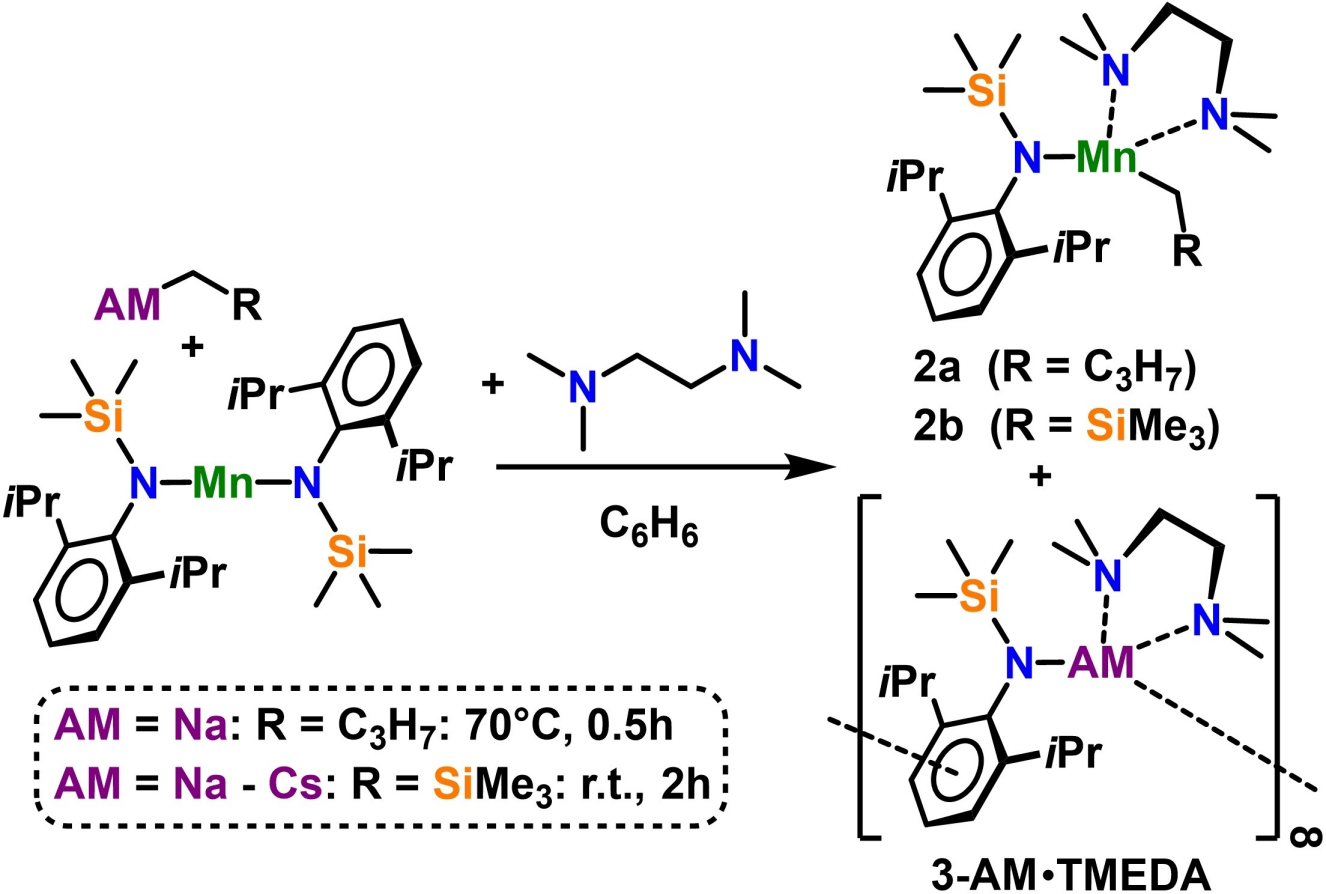
Ligand redistribution of mixed monoalkyl/bisamide sodium, potassium, rubidium, and caesium manganates in presence of TMEDA to give alkali metal amide and heteroleptic monoalkyl/monoamide manganese complexes in 1 : 1 ratios.

Treatment of a manganese bis‐amide solution in benzene with butyl sodium and excess of bidentate nitrogen donor TMEDA, as well as recrystallisation of crude [(Na)Mn(^*n*^Bu)(N^‘Ar^)_2_] in TMEDA/*n*‐hexane mixtures gave two distinct sets of crystals, suitable for X‐ray crystallographic characterisation, which were identified as monometallic complexes [Na(N^‘Ar^)⋅TMEDA]_2_ ^[74]^ and Mn(N^‘Ar^)(^*n*^Bu)⋅TMEDA (**2 a**) (see Scheme 3).

Isolation of neutral manganese complex **2 a** in its pure form is rendered difficult as it exhibits similar solubility to those of the corresponding alkali metal amide by‐products. In general, mixed amido‐alkyl Mn(II) species containing non‐stabilised *n*‐butyl ligands remain scarce in literature,[^14^, ^80^] since β‐hydride abstraction, followed by alkene dissociation is the principal decomposition pathway often observed in these compounds. Crystalline samples of **2 a** did not show any signs of decomposition in argon atmosphere at room temperature over the course of six weeks. Similarly, “β‐stable” Mn(N^‘Ar^)(CH_2_SiMe_3_)⋅TMEDA (**2 b**) cannot be isolated in pure form from this complex destruction pathway and attempts to synthesise **2 b** from stoichiometric mixtures of [{Mn(CH_2_SiMe_3_)_2_}_∞_] and free amine H‐N^‘Ar^ with one equivalent of TMEDA led to intractable product mixtures.

**Figure 2:**
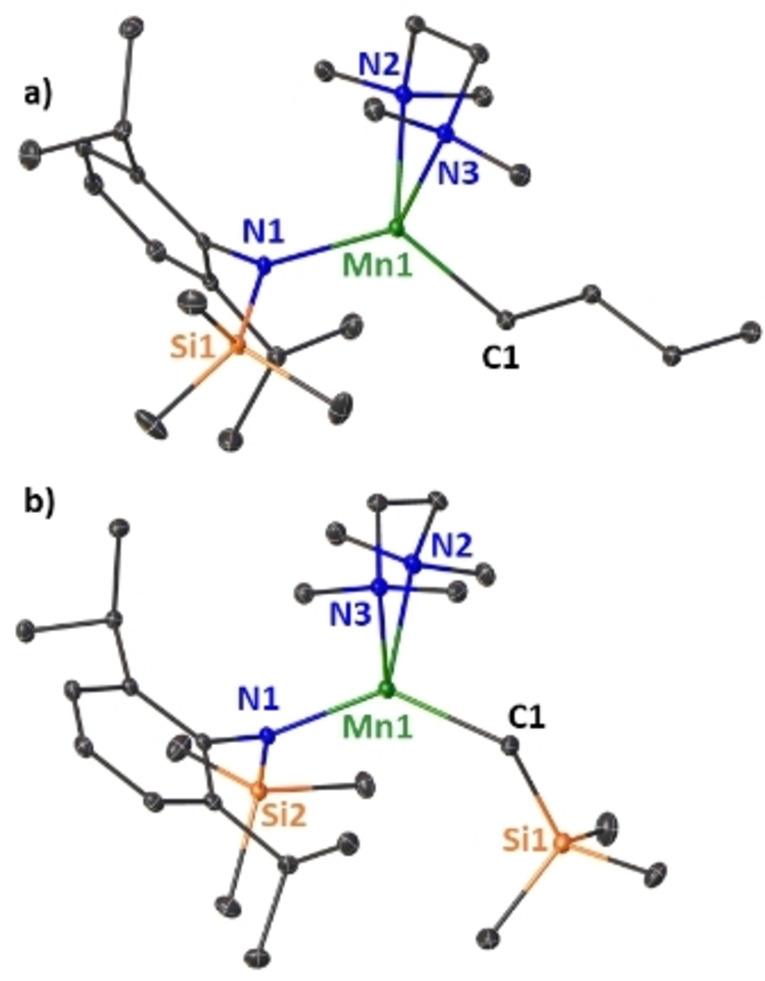
Molecular structures of neutral manganese (II) complexes a) Mn(N^‘Ar^)(^*n*^Bu)⋅TMEDA (**2 a**) and b) Mn(N^‘Ar^)(CH_2_SiMe_3_)⋅TMEDA (**2 b**). Ellipsoids are displayed at 30 % probability and hydrogen atoms are omitted for clarity. Selected bond lengths [Å] and angles [°]: a) Mn1‐C1 2.1479(17); Mn1‐N1 2.0741(12); Mn1‐N2 2.3239(13); Mn1‐N3 2.3042(12); N1‐Mn1‐C1 124.80(6); N2‐Mn1‐N3 79.96(4), b) Mn1‐C1 2.143(2); Mn1‐N1 2.0623(15); Mn1‐N2 2.3166(16); Mn1‐N3 2.3462(15); N1‐Mn1‐C1 135.19(7); N2‐Mn1‐N3 79.94(6).

Determined by X‐ray crystallography **2 a** displays a distorted‐tetrahedral coordination geometry in the solid state, which is typical for four‐coordinate Mn(II) complexes (see Figure 2a).

Mn1 forms short bonds to the anionic α‐carbon of the butyl ligand [Mn1‐C1: 2.1479(17) Å] and the amido‐nitrogen [Mn1‐N1: 2.0741(12) Å] and exhibits longer dative bonds to the TMEDA nitrogen atoms [Mn1‐N2: 2.3239(13) Å, Mn1‐N3: 2.3042(12) Å]. The metal‐TMEDA bite angle (N2‐Mn1‐N3) of 79.96(4)° is somewhat comparable to those of previously reported TMEDA‐ligated manganese(II) complexes, for example Mn(CH_2_SiMe_3_)_2_⋅TMEDA^[76]^ ([79.92(6)°], MnMe_2_⋅TMEDA^[81]^ [75.25(5)°], MnCp_2_⋅TMEDA^[82]^ [78.59(18)°], while the mean bond angle subtended at Mn1 in **2 a** is 104.81°. This distortion from an ideal tetrahedral geometry is pronounced by the widening of the amido‐metal‐alkyl bite angle [N1‐Mn1‐C1: 124.80(6)°], which emphasises the steric hindrance applied by the aryl‐silyl‐amide ligand.

In order to probe the stability, that is, the tendencies for complex dissociation with chelating N‐donor solvents for the heavier group 1 metals, the relevant alkali metal reagent needs to be optimised. The lower stability of the butyl compounds of potassium, rubidium and caesium necessitated the switching to more stable neosilyl‐alkyl reagents AM(CH_2_SiMe_3_)^[61]^ (AM=Na, K, Rb, Cs), in which β‐hydrogen elimination side reactions are impossible. Following the same methodology, AM(CH_2_SiMe_3_) reacts at room temperature with Mn(N^‘Ar^)_2_ to afford the co‐complexed products as a white precipitate from a yellow benzene reaction mixture. However, recrystallisation from a TMEDA\*n*‐hexane solution leads repeatedly to ligand scrambling to yield the monometallic complexes Mn(N^‘Ar^)(CH_2_SiMe_3_)⋅TMEDA (**2 b**) and the corresponding alkali metal amides AM(N^‘Ar^)⋅TMEDA. The lighter sodium and potassium homologues have already been structurally elucidated in the literature [AM=Na: dimeric [Na(N^‘Ar^)⋅TMEDA]_2_,^[74]^ AM=K: polymeric [{K(N^‘Ar^)⋅TMEDA}_∞_,^[74]^ whereas no structural clarification on the heaviest alkali metal amide complexes [{AM(N^‘Ar^)⋅TMEDA}_∞_, AM=Rb (**3‐Rb⋅TMEDA**) and AM=Cs (**3‐Cs⋅TMEDA**) has been collected to this point (see Scheme 3).

Determined by X‐ray crystallography, the molecular structure of **2 b** (see Figure 2b) has a Mn atom in a distorted tetrahedral environment made up by the anionic amide and the neosilyl‐alkyl ligands and is, like compound **2 a**, coordinatively satisfied by TMEDA, which chelates to the metal via its two nitrogen atoms. Akin to its butyl analogue, **2 b** crystallises in a monoclinic space group and the short Mn1‐C1 [2.143(2) Å] bond and Mn1‐N1 [2.0623(15) Å] bond compare favourably to the structurally similar complex **2 a**, with the main difference lying in the wider N1‐Mn1‐C1 bite angle of 135.19(7)° in the neosilyl‐substituted complex **2 b**, which is a consequence of the increased steric bulk induced by the CH_2_SiMe_3_ group.

### Synthesis and characterisation of unsolvated and solvated rubidium and caesium amides and their role in manganate formation

In accordance with previous observations regarding ligand scrambling, carrying out this set of co‐complexation reactions in the presence of tridentate PMDETA, instead of TMEDA, leads to formation of a yet unidentified Mn(N^‘Ar^)(CH_2_SiMe_3_)⋅PMDETA species and homometallic AM(N^‘Ar^)⋅PMDETA (AM=Na: Na(N^‘Ar^)⋅PMDETA,^[74]^ AM=K: [{K(N^‘Ar^)⋅PMDETA}_2_],^[74]^ AM=Rb: [{Rb(N^‘Ar^)⋅PMDETA}_∞_] (**3‐Rb⋅PMDETA**), AM=Cs: [{Cs(N^‘Ar^)⋅PMDETA}_∞_] (**3‐Cs⋅PMDETA**)) respectively.

**Scheme 4:**
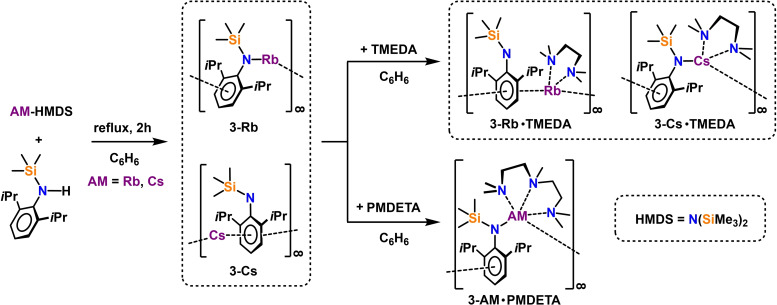
Synthesis of unsolvated alkali metal amides [{AM(N^‘Ar^)}_∞_] (**3‐Rb**: AM=Rb; **3‐Cs**: AM=Cs), TMEDA solvates [{AM(N^‘Ar^)⋅TMEDA}_∞_] (**3‐Rb⋅TMEDA**: AM=Rb; **3‐Cs⋅TMEDA**: AM=Cs) and PMDETA solvates [{AM(N^‘Ar^)⋅PMDETA}_∞_] (**3‐Rb⋅PMDETA**: AM=Rb; **3‐Cs⋅PMDETA**: AM=Cs).

Intrigued by the repeated isolation of solvated heavier group 1 amide by‐products, especially the rubidium and caesium homologues, we set out to find a rational synthetic pathway to increase the body of these species and gain access to the unsolvated parent alkali metal amides. Therefore, a reaction mixture of the literature known AM‐HMDS complex (AM=Rb and Cs) with one equivalent of H‐N^‘Ar^ in benzene solution was heated to reflux in a transamination reaction to yield the desired donor‐free [{Rb(N^‘Ar^)}_∞_] (**3‐Rb**) and [{Cs(N^‘Ar^)}_∞_] (**3‐Cs**), which can be crystallised and isolated from the reaction mixture in 82 % and 75 % yield, respectively (see Scheme 4).

**Figure 3:**
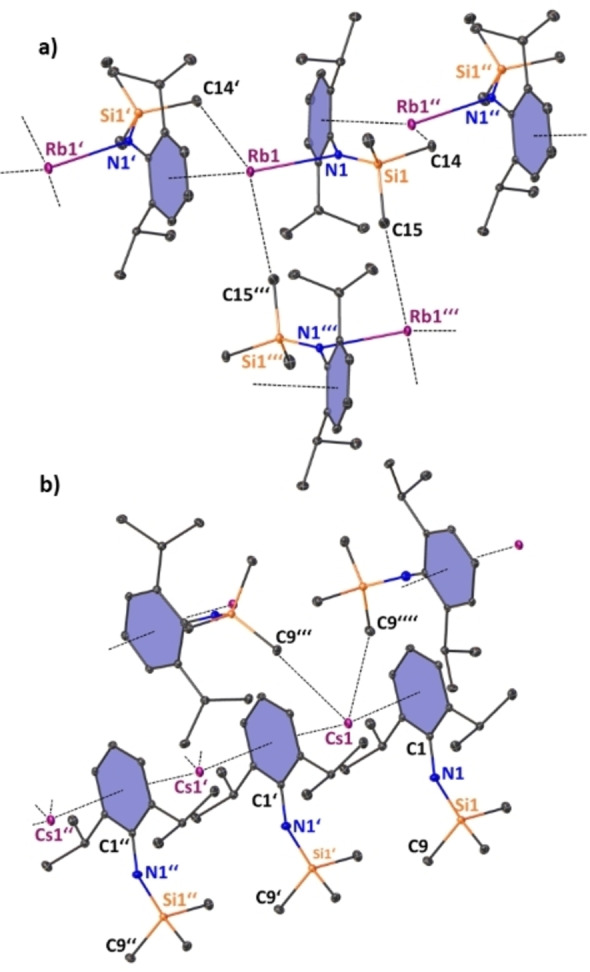
Molecular structures of heavier group 1 amide complexes **3‐AM** a) [{Rb(N^‘Ar^)}_∞_] (**3‐Rb**), symmetry operation to generate equivalent atoms denoted’: +x, 3/2‐y, 1/2 +z,'’: +x, 3/2‐y, −1/2+z and'’’: 1‐x, 1‐y, 1‐z; b) [{Cs(N^‘Ar^)}_∞_] (**3‐Cs**), symmetry operation to generate equivalent atoms denoted’: +x, +y, 1+z,'’: ‐1/2 +y, 1/2 +x, 2+z,'’’: 1‐y, +x, 1‐z and'’’’: ‐1/2 +x, 3/2‐y, 1‐z. Ellipsoids displayed at 30 % probability and H atoms omitted for clarity. Anagostic interactions and π‐arene interactions shown as dashed lines. Ph rings are coloured for better clarity. Selected bond lengths [Å] and angles [°]: a) Rb1‐N1 2.8321(15); Rb1‐C14’ 3.674(3); Rb1‐C15’’ 3.486(2); Rb1‐C_centroid_ 2.960; C1‐N1‐Si1 140.88(13), b) Cs1‐C_centroid_ 3.160; Cs1‐C9’’’ 3.661(1); Cs1‐C9’’’’ 3.661(1); C1‐N1‐Si1 148.8(5).

The rubidium and caesium amide complexes **3‐AM** could be identified by X‐ray crystallography, as well as by one‐ and two‐dimensional NMR and infrared spectroscopy (see Supporting Information for detailed analysis). **3‐Rb** crystalises in the monoclinic space group *P*2_1_/*c* and **3‐Cs** in the tetragonal space group *P*‐42_1_ *m* while both complexes adopt a polymeric chain‐type structure interconnected by attractive intermolecular anagostic interactions in the solid state (see Figure 3).

For rubidium amide **3‐Rb** the formally one‐dimensional polymeric chains propagate parallel to the crystallographic c‐axis through stabilizing Rb⋅⋅⋅π‐arene interactions which are best described as η^6^‐coordinated [Rb⋅⋅⋅C_centroid_: 2.960 Å, range of Rb⋅⋅⋅C_arom_ distances: 3.3046(19) ‐ 3.321(2) Å] and anagostic Rb⋅⋅⋅C(H_3_)SiMe_2_ interactions [Rb⋅⋅⋅C14’=3.674(3) Å]. Neighbouring chains link along the b‐axis through a second anagostic Rb⋅⋅⋅C(H_3_)SiMe_2_ interaction [Rb1⋅⋅⋅C15’’’=3.486(2) Å]. The strongest metal bond in **3‐Rb** is the mostly ionic metal amide bond [Rb1‐N1=2.8322(15) Å], which completes the coordination sphere around the rubidium metal centre leading to a much wider *ipso*‐carbon‐nitrogen‐silicon angle [C1‐N1‐Si1=140.88(13)°] in the amide ligand compared to lighter sodium [Na1‐N1=2.2587(15) Å, C1‐N1‐Si1=123.83(11)°] and potassium [K1‐N1=2.6755(15) Å, C1‐N1‐Si1=137.47(13)°] homologues. The heaviest group 1 amide **3‐Cs** has strikingly different structural properties as the packing in this ionic compound renders the Cs−N distances too long to be considered a chemical bond [Cs1⋅⋅⋅N1=3.725(1) Å and Cs1⋅⋅⋅N1’=4.326(1) Å]. While the crystal structure can still be described as that of a polymeric chain structure, the propagation alongside the crystallographic c‐axis proceeds through a second Cs⋅⋅⋅π‐arene interaction towards the Dipp group of the next asymmetric unit with both aryl‐rings being coordinated in a η^6^‐fashion [Cs⋅⋅⋅C_centroid_: 3.160 Å and 3.166 Å, range of Cs⋅⋅⋅C_arom_ distances: 3.249(1)–3.622(2) Å]. This energetically favourable π‐stabilisation in addition to two intermolecular anagostic Cs⋅⋅⋅C(H_3_)SiMe_2_ interactions [Cs1⋅⋅⋅C9’’’=Cs1⋅⋅⋅C9’’’’=3.661(1) Å] and intramolecular longer Cs⋅⋅⋅C(H_3_)SiMe_2_ contacts towards the isopropyl moieties [Cs1⋅⋅⋅C7=3.679(1) Å] fill the coordination sphere around the large Cs metal. With the metal being more separated from the amide N atom, the corresponding *ipso*‐carbon‐nitrogen‐silicon angle in the amide ligand in **3‐Cs** is widened significantly [C1‐N1‐Si1=148.8(5)°]. In comparison to the vast body of well‐studied lithium amides in the literature, the number of crystallographically characterised polymeric sodium and potassium amides are limited^[83]^ and corresponding polymeric rubidium and caesium amides are scarce.[^45a^, ^50^, ^56^, ^59^, ^60^, ^83k^, ^84^]

**Figure 4:**
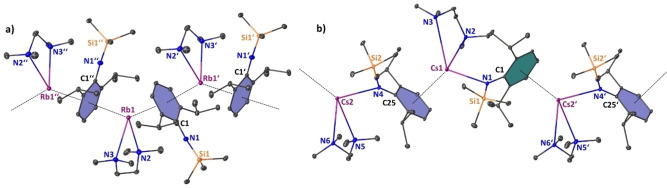
Molecular structures of heavier group 1 amide TMEDA complexes **3‐AM⋅TMEDA** a) [{Rb(N^‘Ar^)⋅TMEDA}_∞_] (**3‐Rb⋅TMEDA**), symmetry operation to generate equivalent atoms denoted’: 1/2 ‐x, 1/2 +y, 1/2 ‐z and'’: 1/2 ‐x, ‐1/2 +y, 1/2 ‐z; b) [{Cs(N^‘Ar^)⋅TMEDA}_∞_] (**3‐Cs⋅TMEDA**), symmetry operation to generate equivalent atoms denoted’: −1+x, +y, +z. Ellipsoids at 30 % probability and H atoms omitted for clarity. Anagostic interaction and π‐arene interactions shown as dashed lines. Ph rings are coloured for better clarity. Selected bond lengths [Å] and angles [°]: a) Rb1‐N2 3.0056(18); Rb1‐N3 3.0035(17); Rb1‐C_centroid_ 3.047 and 3.143; C1‐N1‐Si1 174.41(16); N2‐Rb1‐N3 61.08(5), b) Cs1‐N1 3.067(6); Cs1‐N2 3.216(7); Cs1‐N3 3.425(7); Cs1‐C_centroid_ 3.299; Cs2‐N4 3.089(6); Cs2‐N5 3.284(7); Cs2‐N6 3.167(7); Cs2’‐C_centroid_ 3.325; N2‐Cs1‐N3 58.10(17); N5‐Cs2‐N6 59.73(18); C1‐N1‐Si1 135.4(5); C25‐N4‐Si2 138.3(6).

Suspending **3‐AM** in hexane and subsequent addition of chelating TMEDA led to transparent solutions, which yielded colourless crystals after storage at −20 °C. X‐ray crystallography and one‐ and two‐dimensional NMR spectroscopy confirmed the same products **3‐AM⋅TMEDA**, as have been found in the corresponding dissociation of the co‐complexed manganate. In contrast to the lighter Li to K congeners, **3‐Rb⋅TMEDA** and **3‐Cs⋅TMEDA** are not deaggregated by the donor solvent, but a polymeric chain structure is retained (see Figure 4).

The crystallographic determination of **3‐Rb⋅TMEDA** established it as a contacted TMEDA solvate where the metal centre binds to the neutral nitrogen donor, that is *syn* orientated towards the ligand's N(SiMe_3_) moiety. In contrast to parent amide **3‐Rb**, the absence of interconnecting anagostic contacts leads to an infinite zig‐zag chain in **3‐Rb⋅TMEDA** that propagates parallel to the crystallographic b‐axis through one η^6^‐coordinating Rb⋅⋅⋅π‐arene interaction [Rb1⋅⋅⋅C_centroid_: 3.05 Å, range of Rb1⋅⋅⋅C_arom_ distances: 3.323(2) Å ‐ 3.413(2) Å] and one η^4^‐coordinating Rb⋅⋅⋅π‐arene interaction [Rb1⋅⋅⋅C_centroid_: 3.14 Å, range of Rb1⋅⋅⋅C_arom_ distances: 3.260(19)–3.639(21) Å]. In addition to the metal‐ π interactions the metal's coordination sphere is filled by the two dative Rb nitrogen bonds from TMEDA [Rb1‐N2=3.0056(18) Å, Rb1‐N3=3.0035(17) Å] leading to the absence of an explicit alkali metal amide bond comparable to the structural motif in the heavier donor‐free complex **3‐Cs**. The distances of the rubidium metal to the amide nitrogen atoms in **3‐Rb⋅TMEDA** [Rb1‐N1=4.4575(17) Å and Rb1‐N1’=4.0571(17) Å] are too long to be considered a chemical bond, rendering the Dipp‐N‐(SiMe_3_) moiety to an almost linear geometry [C1‐N1‐Si1=174.41(16)°], which emphasizes the strong tendencies for the soft alkali metal to predominantly bind to softer ligand moieties. Similar to its lighter rubidium homologue the TMEDA complex **3‐Cs⋅TMEDA** crystallises in a monoclinic space group and forms linear but twisted chain structures that propagate alongside the crystallographic a‐axis. However, in contrast to **3‐Rb⋅TMEDA**, there are two similar but crystallographically independent Cs environments. While each metal coordination site is built up by two dative metal nitrogen bonds [mean: Cs‐N_TMEDA_=3.27 Å] towards TMEDA, only one η^6^‐coordinating Cs⋅⋅⋅π‐arene interaction [Cs1⋅⋅⋅C_centroid_: 3.30 Å, range of Cs1⋅⋅⋅C_arom_ distances: 3.444(8)–3.702(8) Å, Cs2⋅⋅⋅C_centroid_: 3.33 Å, range of Cs2⋅⋅⋅C_arom_ distances: 3.439(8)–3.792(7) Å] towards the Dipp group of the amide is formed; whereas a strong metal amide bond [Cs1‐N1=3.067(6) Å, Cs2‐N4=3.090(6) Å] together with weaker Cs^+^⋅⋅⋅Me^δ−^‐Si^δ+^ electrostatic interactions is present at both Cs sites. The Cs coordination to the amide nitrogen consequently narrows the Dipp‐N‐(SiMe_3_) angle [C1‐N1‐Si1=135.5(5)° and C25‐N2‐Si2=138.4(6)°] for both coordination sites in **3‐Cs⋅TMEDA**, so that the SiMe_3_ groups exhibit a *syn* conformation within the chain structure.

**Figure 5:**
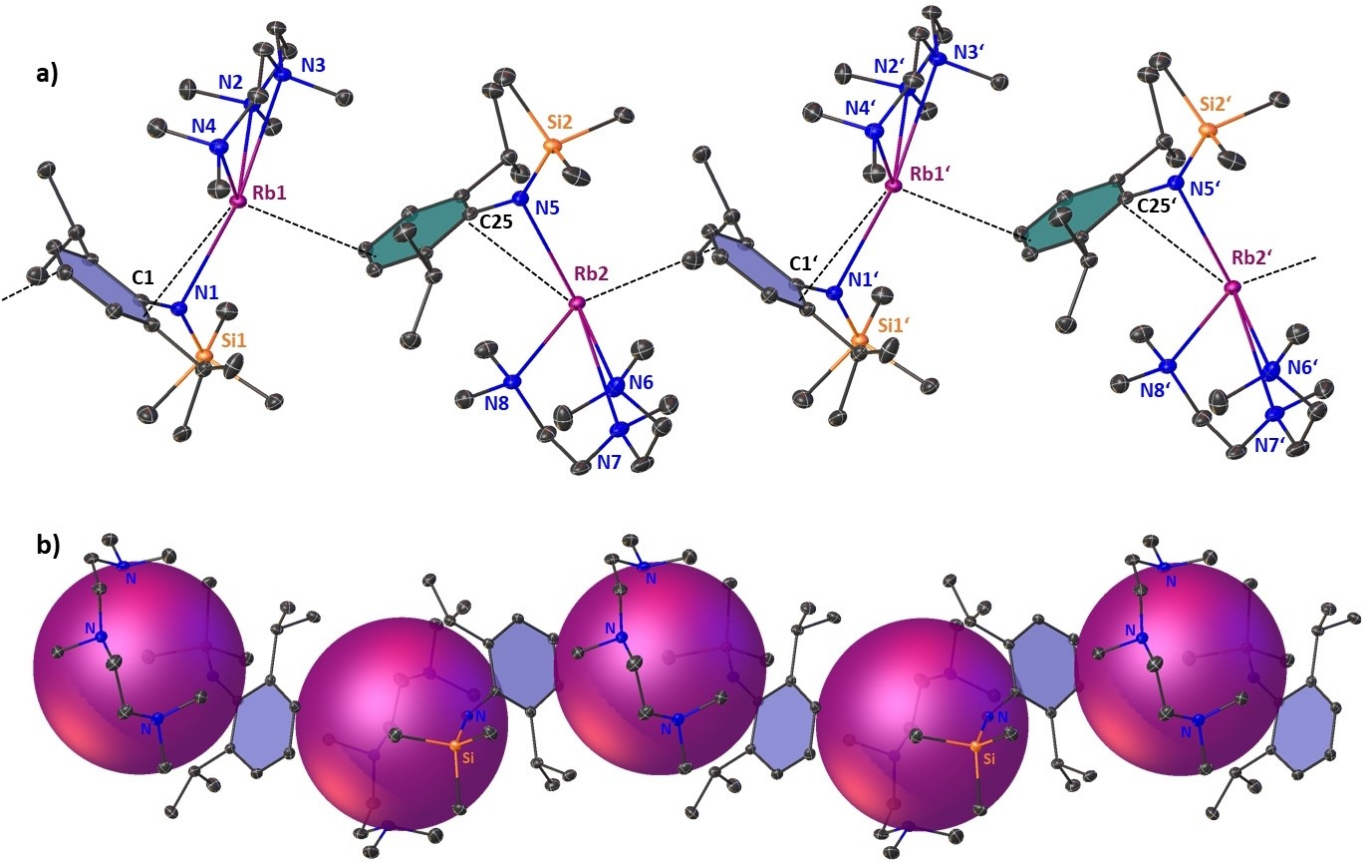
Molecular structure of a) [{Rb(N^‘Ar^)⋅PMDETA}_∞_] (**3‐Rb⋅PMDETA**), symmetry operation to generate equivalent atoms denoted’: 1+x, +y, +z. b) [{Cs(N^‘Ar^)⋅PMDETA}_∞_] (**3‐Cs⋅PMDETA**), non‐metal elements represented as ellipsoids and Cs atoms represented as space filling model. Ellipsoids displayed at 30 % probability and H atoms are omitted for clarity. Anagostic interaction and π‐arene interactions shown as dashed lines. Ph rings are coloured for better clarity. Selected bond lengths [Å] and angles [°]: a) Rb1‐N1 2.966(2); Rb1‐N2 3.043(2); Rb1‐N3 3.041(2); Rb1‐N4 3.049(3); Rb1‐C_centroidblue_ 3.659; Rb1‐C_centroidgreen_ 4.082; Rb2‐N5 2.881(2); Rb2‐N6 3.022(3); Rb2‐N7 3.000(3); Rb2‐N8 3.032(2); Rb2‐C_centroidgreen_ 3.862; Rb2‐C_centroidblue_ 4.103; C1‐N1‐Si1 146.5(2); C25‐N5‐Si2 146.5(2), b) Cs1‐N1 3.035(4); Cs1‐N2 3.162(5); Cs1‐N3 3.219(5); Cs1‐N4 3.153(4); Cs‐C_centroid_ 4.073 and 4.134; C1‐N1‐Si1 152.8(4).

As a previous study has shown, amide salts of the lighter homologues lithium, sodium and potassium AM(N^‘Ar^) (AM=Li−K) can be similarly deaggregated by adding tridentate PMDETA. In the case of Li(N^‘Ar^)⋅PMDETA and Na(N^‘Ar^)⋅PMDETA this deaggregation takes place all the way down to the monomers; whereas the heavier potassium amide PMDETA adduct [{K(N^‘Ar^)⋅PMDETA}_2_] forms a dimer in the solid state.^[74]^ Descending down group 1, suspensions of parent amides **3‐AM** (AM=Rb, Cs) in benzene or hexane treated with PMDETA yielded clear solutions and storing mixtures of **3‐AM** in hexane/PMDETA at −20 °C yielded colourless crystals of the corresponding alkali metal amide donor adduct complexes [{AM(N^‘Ar^)⋅PMDETA}_∞_] (**3‐AM⋅PMDETA**, AM=Rb, Cs). The compounds could be structurally identified by X‐ray crystallography and one‐ and two‐dimensional NMR spectroscopy, confirming the previous findings from the complex destruction pathway in the corresponding bimetallic manganate complexes (see Scheme 4). Unlike in the lighter alkali metal adduct complexes PMDETA is not sufficient to coordinatively saturate the heaviest group 1 metals and therefore **3‐Rb⋅PMDETA** and **3‐Cs⋅PMDETA** exhibit polymeric chain structures similar to their TMEDA analogues in the crystal as depicted in Figure 5.

Unlike in **3‐AM** and **3‐AM⋅TMEDA**, in which a significant structural difference is exhibited between the rubidium and caesium homologue, **3‐Rb⋅PMDETA** and **3‐Cs⋅PMDETA** are structurally more closely related and both compounds crystallise in the monoclinic space group *P*2_1_/*c*. Note that in **3‐Cs⋅PMDETA** the asymmetric unit cell contains two crystallographic independent molecules with slightly different structural parameters, but for brevity only one is discussed here. In the Rb and Cs complex the metal centre occupies a distorted tetragonal pyramidal site stabilised by a strong alkali metal amide bond [Rb1‐N1=2.966(2) Å and Rb2‐N5=2.882(2) Å; Cs1‐N1=3.036(4) Å] and three shorter dative metal‐nitrogen bonds from the PMDETA chelating ligand [mean: Rb‐N_PMDETA_=3.031 Å; mean Cs‐N_PMDETA_=3.178 Å] with the alkali metal being additionally capped by the neighbouring Dipp moiety via η^2^‐coordination to the arene ring in **3‐Rb⋅PMDETA** and with a η^1^‐coordination in the heavier caesium homologue. Encapsulation of the alkali metal in the amide/PMDETA donor pocket in combination with different crystal packing in **3‐AM⋅PMDETA** compared to the packing observed in **3‐Cs** and **3‐Rb⋅TMEDA** (see Figure 5b) seemingly hinders the formation of stronger π‐arene interactions, indicating that the harder, electrostatic interactions in **3‐AM⋅PMDETA** are favoured over ideal π‐arene interactions. A second intramolecular π‐arene interaction of the *ipso*‐carbon with the alkali metal renders the metal in close vicinity, leading to values for the Dipp‐N‐(SiMe_3_) angles C1‐N1‐Si1 [mean: 146.5(2)° in **3‐Rb⋅PMDETA** and 152.8(4)° in **3‐Cs⋅PMDETA**] in the amide ligand that lie in between that of the parent amides **3‐AM** and **3‐Rb⋅TMEDA**.

**Scheme 5:**
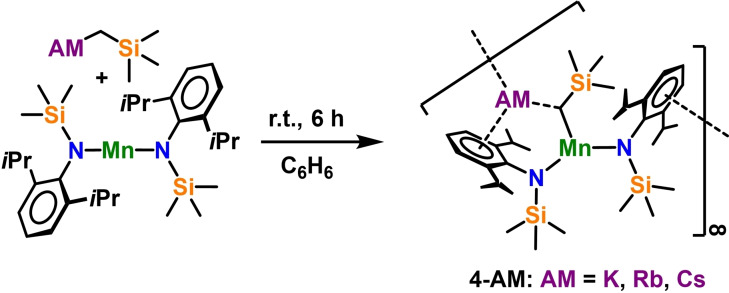
Synthesis of bimetallic ates [{(AM)Mn(CH_2_SiMe_3_)(N^‘Ar^)_2_}_∞_] (**4‐K**: AM=K; **4‐Rb**: AM=Rb; **4‐Cs**: AM=Cs).

As interesting as this structural diversity induced by the usage of chelating nitrogen donors in these monometallic amide complexes is, it severely hindered the successful synthesis of bimetallic manganate species. Thus, co‐complexation reactions with stringent exclusion of donors were carried out. The absence of donor solvent ultimately favours the formation of polymeric bimetallic manganates [{(AM)Mn(CH_2_SiMe_3_)(N^‘Ar^)_2_}_∞_] (**4‐AM**: **4‐K**: AM=K; **4‐Rb**: AM=Rb; **4‐Cs**: AM=Cs), which precipitate from benzene reaction mixtures and can be isolated in reasonable yields of 81 % (AM=K), 52 % (AM=Rb), and 66 % (AM=Cs), respectively (see Scheme 5).

**Figure 6:**
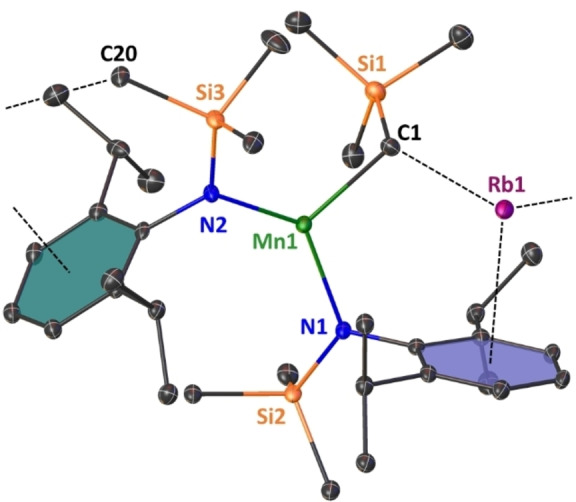
Asymmetric unit of crystalline [{(Rb)Mn(CH_2_SiMe_3_)(N^‘Ar^)_2_}_∞_] (**4‐Rb**). Ellipsoids at 30 % probability and H atoms omitted for clarity. Anagostic interactions and π‐arene interactions shown as dashed lines. Ph rings are coloured for better clarity. Selected bond lengths [Å] and angles [°]: Mn1‐C1 2.165(6); Mn1‐N1 2.090(4); Mn1‐N2 2.068(4); Rb1‐C1 3.189(6); Rb1‐C_centroidblue_ 3.007; Rb1‐C_centroidgreen_ 3.019; Rb1‐C20’ 3.307(6); N1‐Mn1‐N2 130.11(18); N2‐Mn1‐C1 123.1(2), C1‐Mn1‐N1 106.1(2).

**Figure 7:**
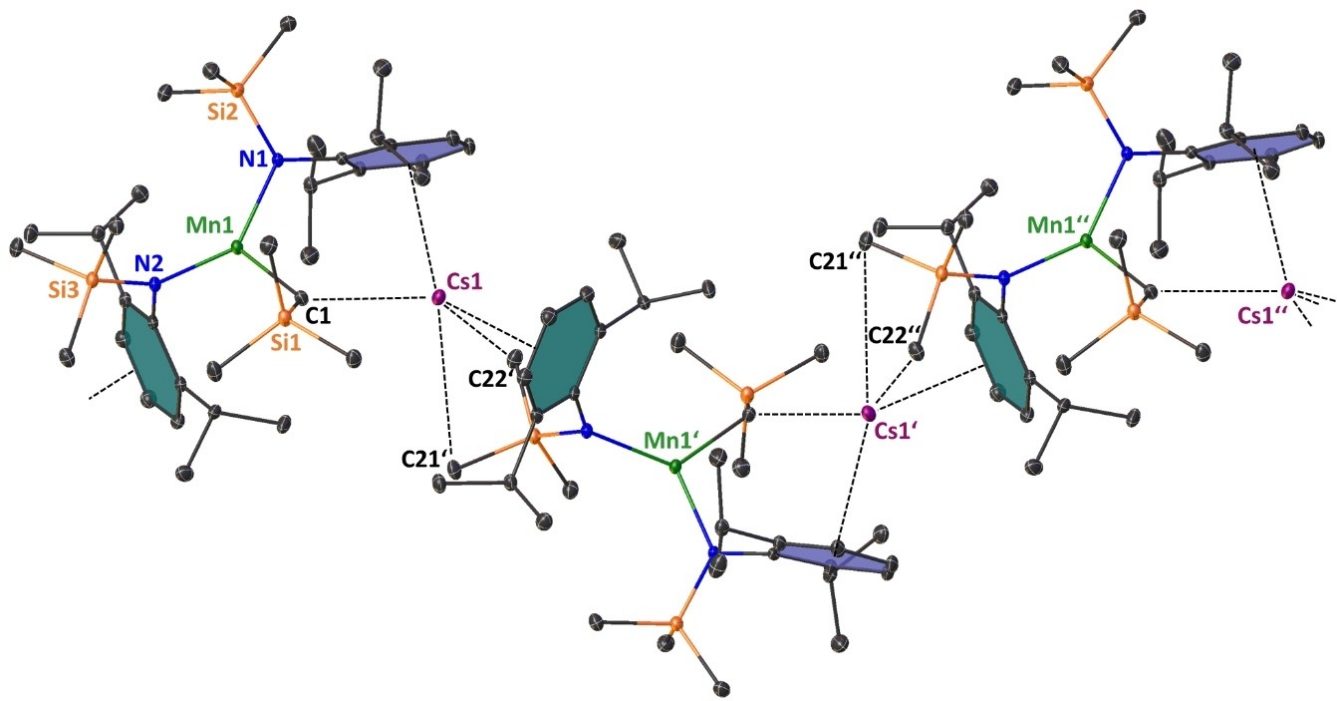
Polymeric section highlighting the zig‐zag chain structure of [{(Cs)Mn(CH_2_SiMe_3_)(N^‘Ar^)_2_}_∞_] (**4‐Cs**). Symmetry operation to generate equivalent atoms denoted’: 3/2 ‐ x, 1/2 +y, 1/2 ‐ z and'’: x, 1+y, z. Ellipsoids at 30 % probability and H atoms are omitted for clarity. Anagostic interactions and π‐arene interactions shown as dashed lines. Ph rings are coloured for better clarity. Selected bond lengths [Å] and angles [°]: Mn1‐C1 2.155(4); Mn1‐N1 2.063(3); Mn1‐N2 2.093(3); Cs1‐C1 3.413(4); Cs1‐C_centroidblue_ 3.185; Cs1‐C_centroidgreen_ 3.189; Cs1‐C21’ 4.102(5); Cs1‐C22’ 3.699(4); N1‐Mn1‐N2 128.57(12); N2‐Mn1‐C1 125.54(13), C1‐Mn1‐N1 105.69(13).

Recrystallisation from toluene solutions via slow diffusion‐layering with *n*‐hexane yielded single crystals of [{(Rb)Mn(CH_2_SiMe_3_)(N^‘Ar^)_2_}_∞_] (**4‐Rb**) and isostructural [{(Cs)Mn(CH_2_SiMe_3_)(N^‘Ar^)_2_}_∞_] (**4‐Cs**) amenable to X‐ray crystallographic determination (see Figures 6 and 7) and all heavier manganates **4‐AM** were characterised via elemental analysis and infrared spectroscopy (see Supporting Information for details). Attempts to record meaningful NMR spectra in benzene‐*d_6_* and THF‐*d_8_* were unsuccessful due to the paramagnetic nature of Mn(II). A solution state magnetic measurement of **4‐AM** via the Evans method gave *μ*_eff_ values (**4‐K**: 5.81 *μ*_B_, **4‐Rb**: 6.12 *μ*_B_, **4‐Cs**: 5.69 *μ*_B_) that lie within the range of the expected spin‐only value for a high spin Mn^II^‐d^5^ configuration (*μ*_S.O._=5.92 *μ*_B_).

The heavier group 1 metal manganate complexes **4‐Rb** and **4‐Cs** crystallise in the monoclinic primitive space groups P2_1_\c and P2_1_\n, respectively. Both compounds form infinite zig‐zag type chain structures, in which the tris‐coordinate manganate centres are interconnected via group 1 metals engaging in stabilizing AM⋅⋅⋅π‐arene interactions. Each alkali metal is coordinatively satisfied by interaction with the alkyl ligand's alpha carbon atom [in **4‐Rb**: Rb1‐C1=3.189(6) Å; in **4‐Cs**: Cs1‐C1=3.413(4) Å], with one η^6^‐coordination to one aryl unit within the asymmetric unit and one η^6^‐coordination with an aryl unit within the neighbouring unit, thus propagating through the crystallographic c‐axis in the case of **4‐Rb** and the crystallographic b‐axis for **4‐Cs**. The coordination environment surrounding the Mn(II) centre is best described as distorted trigonal planar with the sum of bond angles around the Mn centre being 359.32° in **4‐Rb** and 359.79° in **4‐Cs**. The mean Mn amide bond lengths (**4‐Rb**: Mn1‐N, 2.079 Å; **5‐Cs**: Mn1‐N=2.078 Å) and manganese carbon bond length [in **4‐Rb**: Mn1‐C1=2.165(6) Å; in **4‐Cs**: Mn1‐C1=2.155(4) Å] match in dimensions, not only showing the structural similarity of the manganate core within the two complexes, but also being consistent with the fact that the group 1 metal and the manganate ion are predominately electrostatic in nature. A possible flexibility of the system regarding the compensation of the group 1 metal's size is exerted by rotation of one silyl group. In **4‐Rb** all three silyl moieties are orientated in a facial way allowing for a shorter intermolecular anagostic Rb1⋅⋅⋅C(H_3_)SiMe_2_ contact [Rb1‐C20=3.307(6) Å], while **4‐Cs** exhibits one longer and one shorter anagostic Cs1⋅⋅⋅HCH_2_SiMe_2_ contact [Cs1⋅⋅⋅C21’=4.103(5) Å and Cs1⋅⋅⋅C22’=3.699(4) Å]. These AM^+1^⋅⋅⋅Me^δ−^‐Si^δ+^ electrostatic interactions are commonly observed in s‐block chemistry in general^[85]^ and rely heavily on the induced polarisation by the main group metal, so they are especially strong regarding the large difference in electronegativity of the Si atom and the heavier group 1 metals Rb and Cs.[^52^, ^86^]

Exposure of manganates **4‐AM** (AM=K, Rb and Cs) in the aromatic solvents benzene or toluene to the coordinating oxygen donors Et_2_O or THF did not yield crystalline material suitable for X‐ray crystallographic analysis, which may be explained by a lower tendency of these non‐chelating ligands to disintegrate the bimetallic species. In contrast to previously reported Mn(II) metallators^[18]^ extended heating of benzene or toluene solutions of manganates **4‐AM** did not show any signs of direct manganation or group 1 arene metallation reactions, which emphasises the importance of template effects that play a crucial role in AMM*Mn* reactions.

## Conclusion

In conclusion, we have shown that treatment of literature known manganese‐bisamide Mn(N^‘Ar^)_2_ with alkyl lithium reagents give reaction mixtures with limited stability under investigated conditions and exclusively led to twofold ligand exchanged monometallic lithium amide and bis‐alkyl‐manganese compounds. Addition of the polydentate coordinating solvents TMEDA and PMDETA allowed for more detailed structural identification of heteroleptic, monometallic alkyl‐amido Mn(II) complexes **2 a** and **2 b** which were crystallographically characterised, as well as the monometallic alkali metal amide complexes **3‐AM⋅TMEDA** and **3‐AM⋅PMDETA** (AM=Rb, Cs), which in contrast to their lighter homologues adopt infinite linear or zig‐zag arrangements as identified in their solid state structures. Structural diversity in these adduct‐complexes and in the parent, donor‐free alkali metal amide compounds **3‐Rb** and **3‐Cs**, obtained from a transamination reaction, is discussed, emphasising the complexity in seemingly simple alkali metal amide systems. The co‐complexation of butyl‐sodium with said Mn(II) amide yields monomeric, bimetallic sodium manganate **1** in diethyl ether, a potential metalating agent in AMM*Mn* chemistry, which will be targeted in future work. The first structurally characterised Rb‐manganate **4‐Rb** with manganese solely in the oxidation state +2 is reported, together with its heavier Cs‐homologue **4‐Cs**, which represents the first example of a mixed amido‐alkyl caesium manganate. Presumably the AM‐arene interactions of the heavier AM metals Rb and Cs favour formation of the bimetallic species, contrary to the lighter Li and Na homologues in which these arene interactions are less pronounced, which leads to ligand redistribution reactions. Likewise, adding the donor solvents TMEDA and PMDETA may block the coordination sites of the AM metals, which competes with the AM‐arene interactions, thus favouring AM cleavage and complex destruction.

With these alkali metal complexes now in hand, future work will investigate the application of these complexes in stoichiometric and catalytic reactions.

## Experimental Section

Experimental details can be found in the Supporting Information.

Deposition Number(s) 2175911, 2175912, 2175913, 2175914, 2175915, 2175916, 2175917, 2175918, 2175919, 2175920, 2175921 and 2175922 contain(s) the supplementary crystallographic data for this paper. These data are provided free of charge by the joint Cambridge Crystallographic Data Centre and Fachinformationszentrum Karlsruhe Access Structures service.

## Conflict of interest

The authors declare no conflict of interest.

1

## Supporting information

As a service to our authors and readers, this journal provides supporting information supplied by the authors. Such materials are peer reviewed and may be re‐organized for online delivery, but are not copy‐edited or typeset. Technical support issues arising from supporting information (other than missing files) should be addressed to the authors.

Supporting Information

## Data Availability

A dataset underlying this research can be located at https://doi.org/10.15129/7fc4c5a1‐535a–4204‐bee8‐58821f91b22d
