# Supplementary material for: Heavy Alkali Metal Manganate Complexes: Synthesis, Structures and Solvent‐Induced Dissociation Effects
Source: Chemistry. 2022 Aug 3;28(55):e202201716. doi: 10.1002/chem.202201716 (PMC9804227; doi:10.1002/chem.202201716)
Supplement: Supplementary file 1 — Supporting Information [file CHEM-28-0-s001.pdf]

Chemistry–A European Journal

Supporting Information

Heavy Alkali Metal Manganate Complexes: Synthesis, Structures and Solvent-Induced Dissociation Effects

Gerd M. Ballmann, Thomas X. Gentner, Alan R. Kennedy, Eva Hevia, and Robert E. Mulvey*

Contents

1) General experimental procedures and complex syntheses S 2

2) Crystal structure data S 8

3) Selected ^{1}H-, ^{13}C-, DEPT-135-, DEPT-90-, ^{1}H-^{1}H COSY-, HSQC-, HMBC-, ^{29}Si- and paramagnetic NMR spectra S33

4) Selected infrared spectra S61

5) References S64

1) General experimental procedures and complex syntheses

General experimental procedures

All experiments were carried out in dry glassware under N_2 or Ar using standard Schlenk techniques. Hexane, THF and diethyl ether were dried by heating to reflux over sodium benzophenone ketyl and then distilled under nitrogen prior to use. Pentane, benzene, and toluene were degassed with nitrogen, dried over activated aluminium oxide (Innovative Technology, Pure Solv 400-4-MD, Solvent Purification System), and then stored under inert atmosphere over activated 4 Å molecular sieves. TMEDA and PMDETA were dried over CaH_2, distilled, and stored over activated 4 Å molecular sieves prior to use. Starting materials and research chemicals were obtained from commercial suppliers where appropriate and used without further purification. H{N($SiMe_3$)Dipp} was prepared via a modified one-pot synthesis, originally described by Wigley.[1] Li{N($SiMe_3$)Dipp} was prepared according to a modified literature procedure from Roesky.[2] ^{n}BuNa,[3] Na(CH_2SiMe_3),[4] and K(CH_2SiMe_3)[5] were synthesized according to previously published methods. [Rb{N($SiMe_3$)$_2$}]$_2$[6] and [Cs{N($SiMe_3$)$_2$}]$_2$[6] were prepared according to the halide exchange methodology of O´Hara.[7] The literature known methodology for the synthesis of Rb(CH_2SiMe_3)[8] and Cs(CH_2SiMe_3)[8] was followed but instead of the *tert*-butyl alkali metal precursors, the more soluble Rb{OC(Me)$_2$Et}[9] and Cs{OC(Me)$_2$Et}[9] precursors were used. C_6D_6, toluene-d_8 and THF-d_8 were degassed by freeze-pump-thaw methods and stored over activated 4 Å molecular sieves. NMR spectra were recorded on a Bruker AV3 or AV 400 MHz spectrometer operating at 400.13 MHz for ^{1}H, 100.62 MHz for ^{13}C. All ^{13}C spectra were proton decoupled. ^{1}H and ^{13}C{^{1}H} chemical shifts are expressed in parts per million (δ, ppm) and referenced to residual solvent peaks. Coupling constants (*J*) are given in Hertz (Hz). For describing signal multiplicities common abbreviations were used: s (singlet), d (doublet), t (triplet), q (quartet), hept (heptet), m (multiplet) and br (broad). Solution magnetic susceptibilities were determined by the Evans method at 300 K.[10–12] Infrared spectra of starting materials and selected products were obtained as Nujol mulls on NaCl plates. Mulls were prepared in the glove box using anhydrous Nujol, which was dried and stored over activated 4 Å molecular sieves under argon, and then transferred to the spectrometer in a desiccator. Spectra were recorded on a Nicolet 360 FTIR spectrometer spanning 4000-400 cm^{-1}. The melting points of selected products and starting materials were determined as follows. A small sample of crystalline/powdered material was loaded into a melting point tube in the glove box. This tube was then sealed with Plasticine® before removal from the glove box. The melting point was then determined in the usual manner using a Buchi Melting Point B-545 apparatus. Elemental analysis was conducted by the Elemental Analysis Service at London Metropolitan University. Crystallographic data for complexes **1** (CCDC2175912), **2a** (CCDC2175913), **2b** (CCDC2175914), **3-Rb** (CCDC2175918), **3-Cs** (CCDC2175915), **3-Rb·TMEDA** (CCDC2175920), **3-Cs·TMEDA** (CCDC2175917), **3-Rb·PMDETA** (CCDC2175919), **3-Cs·PMDETA** (CCDC2175916), **4-Rb** (CCDC2175922) and **4-Cs** (CCDC2175921) were collected on an Oxford Diffraction Gemini S instrument with graphite-monochromated Mo−Kα (λ 0.71073 Å) radiation or on Rigaku XtaLAB Synergy-S with monochromated Cu−Kα (λ 1.54184 Å) radiation. The measured data was processed with the CrysAlisPro[13] software package. Using Olex2,[14] the structure was solved with the ShelXT[15] structure solution program using Intrinsic Phasing and refined with the ShelXL[16] refinement package using Least Squares minimization or by the full-matrix least-squares method using SHELXL-2018 implemented within WINGX.[15,17] All non-hydrogen atoms were refined using anisotropic thermal parameters unless noted otherwise.

Complex syntheses

Synthesis of $Mn(N^{`Ar})_2$

Although the compound is well known in the literature, the synthesis procedure is described here to highlight its relevance to our work. The same methodology established by the groups of Roesky[18] and Bontemps[19] was followed: A 250 mL J. Young Schlenk flask was charged with anhydrous $MnCl_2$ (1.150 g, 9.139 mmol, 1.00 equiv.) and suspended in dry Et_2O (80 mL). $[\{Li(N^{`Ar})\}_2]$ (4.600 g, 18.01 mmol, 1.97 equiv.) was added and the greyish reaction mixture was stirred at room temperature for 4 days. All volatiles were removed under vacuum and the residue was extracted with pentane (5x 20 mL). The combined yellow filtrates were concentrated and stored at −20 °C. After several days, yellow crystals of the title compound were isolated in 72 % (3.624 g, 6.570 mmol) yield. Crystals suitable for x-ray diffraction analysis were obtained directly from this synthetic procedure.

m.p.: 173 °C – 176 °C (dec.)

A satisfactory elemental analysis for the bulk material of $Mn(N^{`Ar})_2$was not obtained, which may be attributed to decomposition during shipping and/or sample preparation. Best values are given, nevertheless. Elemental analysis: Calculated values for $C_{30}H_{52}N_2Si_2Mn$ (551.87 g/mol): C 65.29, H 9.50, N 5.08; Found: C 63.85, H 9.51, N 5.20.

IR (Nujol, cm^{-1}): 1\λ= 1587 (w), 1426 (s), 1313 (s), 1243 (s), 1192 (s), 1110 (m), 1040 (m), 926 (s), 844 (s), 823 (s), 790 (s), 672 (m), 535 (m).

Synthesis of $[(Na)Mn(^nBu)(N^{`Ar})_2(OEt_2)_2]$ (1)

In a 25 mL Schlenk flask $Mn(N^{`Ar})_2$ (454 mg, 0.823 mmol) was dissolved in C_6H_6 (14 mL) to give a yellow solution. Freshly prepared Na^nBu (66 mg, 0.824 mmol, 1.00 equiv.) was added and the resulting yellow reaction mixture was heated to 70 °C for 30 minutes to give a yellow solution. The solvent was removed in high vacuum to give a white solid, which is triturated with *n*-hexane (10 mL), filtered off and washed with *n*-hexane (2 x 4 mL). Drying at high vacuum yielded a white solid and crystals of the title compound suitable for single crystal X-ray diffraction analysis were grown by slow diffusion of *n*-hexane into a concentrated Et2O solution at -20 °C in 71 % (454 mg, 0.582 mmol) yield.

Solution magnetic moment (THF-*d8*, 300 K) = 5.94 μ_B

m.p.: 68 °C – 71 °C (dec.)

A satisfactory elemental analysis for the bulk material of **1** was not obtained, which may be attributed to decomposition during shipping and/or sample preparation. Carbon and hydrogen values are too low, which hints towards loss of Et_2O molecules. Best values are given, nevertheless. Elemental analysis: Calculated values for $C_{38}H_{71}MnN_2NaOSi_2$ (706.10 g/mol): C 64.64, H 10.14, N 3.97; Found: C 62.98, H 9.92, N 4.01.

IR (Nujol, cm^{-1}): 1\λ= 1417 (m), 1311 (m), 1240 (s), 1195 (m), 1100 (m), 1039 (m), 936 (m), 834 (m), 789 (m), 743 (w), 662 (w).

Synthesis of Mn($N^{`Ar}$)(^{n}Bu)·TMEDA (2a)

A 25 mL Schlenk flask was charged with Mn($N^{`Ar}$)$_2$ (454 mg, 0.823 mmol) and freshly prepared Na^{n}Bu (66 mg, 0.824 mmol, 1.00 equiv.). Benzene (14 mL) was added, and the yellow reaction mixture was heated to 70 °C for 30 minutes to give a clear yellow solution. Removal of the solvent under high vacuum gave a white solid (454 mg). Single crystals of the title compound suitable for X-ray diffraction analysis were obtained by dissolving the crude white solid in a *n*-hexane/TMEDA mixture and crystallisation of the colourless solution at -20 °C to afforded colourless blocks. The crystalline yield was not determined. This complex decomposition process is not considered a reproducible synthetic procedure.

Synthesis of Mn($N^{`Ar}$)(CH_2SiMe_3)·TMEDA (2b)

From Li-precursor: In a 25 mL Schlenk flask Mn($N^{`Ar}$)$_2$ (359 mg, 0.651 mmol) was suspended in *n*-hexane (10 mL) to give a yellow cloudy solution within stirring the reaction mixture for 30 minutes. A solution of Li(CH_2SiMe_3) (64 mg, 0.680 mmol, 1.05 equiv.) in *n*-hexane (5 mL) was added at room temperature and the reaction mixture was stirred at room temperature for 2 hours. The solvent was removed in vacuo yielding a beige solid (299 mg). Recrystallisation from a concentrated solution in *n*-hexane (1 mL) and TMEDA (0.100 mL, 1.11 mmol, 1.72 equiv.) at -20 °C yielded colourless crystalline blocks. The yield was not determined. This complex decomposition process is not considered a reproducible synthetic procedure.

From heavier AM-precursors (AM = Na, K, Rb, Cs): A similar methodology as with Li(CH_2SiMe_3) was followed. Due to solubility the preparation of the title compound with AM(CH_2SiMe_3) (AM = Na, K, Rb, Cs) needs to be conducted in benzene. Cs(CH_2SiMe_3) readily reacts and decomposes in neat benzene, thus Cs(CH_2SiMe_3) is added as a solid in one portion at room temperature. Removal of the benzene solvent and recrystallisation of the crude reaction mixture from a concentrated solution of *n*-

hexane/TMEDA at -20 °C yielded similarly the title compound as colourless crystalline blocks. The yield was not determined. This complex decomposition process is not considered a reproducible synthetic procedure.

Synthesis of [{Rb(N`Ar)}n] (3-Rb)

In a 25 mL Schlenk flask $[Rb\{N(SiMe_3)_2\}]_2$ (1.23 g, 5.00 mmol) was dissolved in C_6H_6 (10 mL) to give a colourless solution. $HN^{`Ar}$ (1.25 g, 5.00 mmol, 1.00 equiv.) was added and the light-yellow reaction mixture was heated to reflux for two hours. Upon cooling to ambient temperatures crystalline material precipitated, which was collected via filtration and subsequently washed with *n*-hexane (3 x 5 mL). Drying at high vacuum the target compound was obtained as white crystalline solid in 82 % (1.37 g, 4.11 mmol) yield.

^{1}H NMR (400 MHz, C_6D_6:THF-*d8* (10:1), 25 °C): δ = 6.97 (d, *J* = 7.1 Hz, 2H, Dipp-C*H*), 6.42 (t, *J* = 7.3 Hz, 1H, Dipp-C*H*), 3.78 – 4.00 (m, 2H, C*H*$(CH_3)_2$), 1.24 (d, *J* = 6.7 Hz, 12H, CH$(CH_3)_2$), 0.36 (s, 9H, Si$(CH_3)_3$).

^{13}C{^{1}H} NMR (101 MHz, C_6D_6:THF-*d8* (10:1), 25°C): δ = 156.9 (C_{arom}), 140.8 (C_{arom}), 122.9 (C_{arom}), 110.0 (C_{arom}), 27.3 (C_{aliph}), 24.5 (C_{aliph}), 5.5 (C_{aliph}).

m.p.: 193 °C – 199 °C (dec.)

Elemental analysis: Calculated values for $C_{15}H_{26}NRbSi$ (333.93 g/mol): C 53.95, H 7.85, N 4.19; Found: C 53.64, H 8.17, N 4.06.

IR (Nujol, cm^{-1}): 1\λ= 1577 (m), 1348 (s), 1291 (m), 1260 (m), 1232 (m), 1134 (m), 1103 (m), 1044 (m), 985 (m), 907 (m), 837 (s), 806 (m), 753 (m), 726 (w), 648 (w).

Synthesis of [{Cs(N`Ar)}∞] (3-Cs)

In a 25 mL Schlenk flask $[Cs\{N(SiMe_3)_2\}]_2$ (1.47 g, 5.00 mmol) was dissolved in C_6H_6 (15 mL) to give a colourless solution. $HN^{`Ar}$ (1.25 g, 5.00 mmol, 1.00 equiv.) was added and the light-yellow reaction mixture was heated to reflux for two hours. Upon cooling to ambient temperatures crystalline material precipitated, which was collected via filtration and subsequently washed with *n*-hexane (3 x 5 mL). Drying at high vacuum the target compound was obtained as white crystalline solid in 75 % (1.43 g, 3.74 mmol) yield.

^{1}H NMR (400 MHz, C_6D_6:THF-*d8* (10:1), 25 °C): δ = 6.95 (d, *J* = 7.3 Hz, 2H, Dipp-C*H*), 6.40 (t, *J* = 7.3 Hz, 1H, Dipp-C*H*), 3.82 (hept, *J* = 6.9 Hz, 2H, C*H*$(CH_3)_2$), 1.23 (d, *J* = 6.9 Hz, 12H, CH$(CH_3)_2$), 0.35 (s, 9H, Si$(CH_3)_3$).

$^{13}C\{^1H\}$ NMR (101 MHz, C_6D_6:THF-*d8* (10:1), 25°C): δ = 156.5 (C_{arom}), 140.5 (C_{arom}), 123.1 (C_{arom}), 109.7 (C_{arom}), 27.4 (C_{aliph}), 24.4 (C_{aliph}), 5.6 (C_{aliph}).

m.p.: 259 °C – 262 °C (dec.)

Elemental analysis: Calculated values for $C_{15}H_{26}NCsSi$ (381.37 g/mol): C 47.33, H 6.62, N 3.42; Found: C 47.24, H 6.87, N 3.67.

IR (Nujol, cm^{-1}): 1\λ= 1572 (m), 1520 (s), 1348 (m), 1302 (w), 1264 (m), 1233 (m), 1222 (m), 1131 (w), 1105 (w), 1046 (m), 998 (m), 933 (w), 845 (m), 815 (m), 748 (s), 652 (w).

Synthesis of [{KMn(CH_2SiMe_3)($N^{`Ar}$)$_2$}$_\infty$] (4-K)

In a 25 mL Schlenk flask Mn($N^{`Ar}$)$_2$ (324 mg, 0.587 mmol) was dissolved in C_6H_6 (10 mL) to give a yellow solution. Upon stirring K(CH_2SiMe_3) (74 mg, 0.586 mmol, 1.00 equiv.) was added in small portions resulting in the precipitation of a white solid. After stirring for further 6 hours at room temperature the precipitate was collected via filtration and subsequently washed with benzene (2 x 3 mL) and with *n*-hexane (2 x 3 mL). Drying at high vacuum the target compound was obtained as white solid in 81 % (321 mg, 0.473 mmol) yield. Despite many attempts, crystals suitable for single crystal X-ray diffraction analysis were not obtained.

Solution magnetic moment (THF-*d8*, 300 K) = 5.81 μ_B

m.p.: 242 °C – 246 °C (dec.)

Elemental analysis: Calculated values for $C_{34}H_{63}N_2Si_3MnK$ (678.18 g/mol): C 60.22, H 9.36, N 4.13; Found: C 59.88, H 9.11, N 3.97.

IR (Nujol, cm^{-1}): 1\λ= 1415 (m), 1361 (w), 1310 (m), 1253 (m), 1236 (m), 1192 (m), 1105 (w), 1039 (w), 917 (m), 834 (m), 784 (m), 730 (w), 661 (w).

Synthesis of [{RbMn(CH_2SiMe_3)($N^{`Ar}$)$_2$}$_\infty$] (4-Rb)

In a 25 mL Schlenk flask Mn($N^{`Ar}$)$_2$ (554 mg, 1.00 mmol) was dissolved in C_6H_6 (12 mL) to give a yellow solution. Upon stirring Rb(CH_2SiMe_3) (173 mg, 1.00 mmol, 1.00 equiv.) was added in small portions resulting in the precipitation of a white solid. After stirring for further 6 hours at room temperature the precipitate was collected via filtration and subsequently washed with benzene (2 x 3 mL) and with *n*-hexane (2 x 3 mL). Drying at high vacuum the target compound was obtained as white solid in 52 %

(377 mg, 0.520 mmol) yield. Crystals suitable for single crystal X-ray diffraction analysis were grown from a concentrated toluene solution layered with *n*-hexane at -20 °C.

Solution magnetic moment (THF-*d8*, 300 K) = 6.12 μ_B

m.p.: 236 °C – 238 °C (dec.)

Elemental analysis: Calculated values for $C_{34}H_{63}N_2Si_3MnRb$ (724.55 g/mol): C 56.36, H 8.76, N 3.87; Found: C 56.34, H 8.71, N 3.50.

IR (Nujol, cm^{-1}): 1\λ= 1415 (w), 1310 (w), 1256 (w), 1235 (m), 1193 (w), 1104 (w), 1038 (w), 918 (w), 833 (w), 784 (w), 726 (w).

Synthesis of [{CsMn(CH$_2$SiMe$_3$)(N$^{`Ar}$)$_2$}$_\infty$] (4-Cs)

In a 25 mL Schlenk flask Mn(N$^{`Ar}$)$_2$ (529 mg, 0.959 mmol) was dissolved in C_6H_6 (8 mL) to give a yellow solution. Cs(CH$_2$SiMe$_3$) (201 mg, 0.957 mmol, 1.00 equiv.) was added in small portions and the resulting yellow reaction mixture is stirred for 6 hours at room temperature. The solvent was removed in high vacuum to give a slightly yellow solid, which was triturated with *n*-hexane (15 mL), filtered off and washed with *n*-hexane (3 x 6 mL). Drying at high vacuum the target compound was obtained as white solid in 66 % (490 mg, 0.520 mmol) yield. Crystals suitable for single crystal X-ray diffraction analysis were grown from a concentrated toluene solution layered with *n*-hexane at -20 °C.

Solution magnetic moment (THF-*d8*, 300 K) = 5.69 μ_B

m.p.: 134 °C – 137 °C (dec.)

Elemental analysis: Calculated values for $C_{34}H_{63}N_2Si_3MnCs$ (771.99 g/mol): C 52.90, H 8.23, N 3.63; Found: C 53.19, H 8.10, N 3.30.

IR (Nujol, cm^{-1}): 1\λ= 15787 (w), 1313 (w), 1256 (w), 1237 (m), 1093 (w), 1018 (w), 868 (s), 837 (m), 816 (m), 768 (w), 734 (w), 644 (w).

2) Crystal structure data

Crystal structure of Mn{N($SiMe_3$)Dipp}$_2$

A yellow crystal of Mn{N($SiMe_3$)Dipp}$_2$ was embedded in inert perfluoropolyalkylether (viscosity 1800 cSt; ABCR GmbH) and mounted using a glass fiber. The crystal was then flash cooled to 123 K in a nitrogen gas stream and kept at this temperature during the experiment. The crystal structure was measured with an Oxford Diffraction Gemini E instrument with graphite-monochromated Mo−Kα (λ = 0.71073 Å) radiation. The measured data was processed with the CrysAlisPro[13] software package. Using Olex2[14], the structure was solved with the ShelXT[15] structure solution program and refined to convergence against F^2 for all independent reflections. All non-hydrogen atoms were refined using anisotropic thermal parameters.

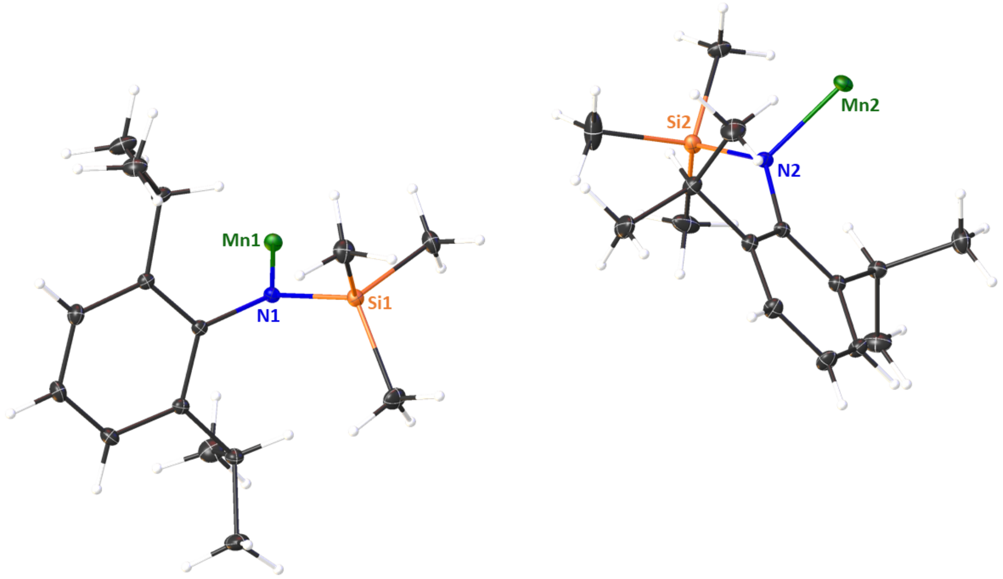

Figure S1. ORTEP plot of the contents of the asymmetric unit of Mn{N($SiMe_3$)Dipp}$_2$ showing two halves of the two independent molecules (ellipsoids drawn at 30% probability).

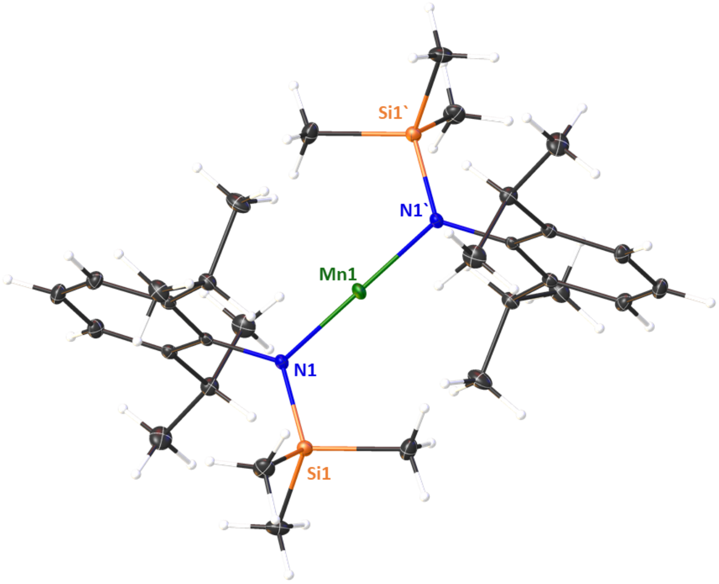

Figure S2. ORTEP plot of one of the crystallographically independent molecules of Mn{N(SiMe$_3$)Dipp}$_2$ (ellipsoids drawn at 30% probability).

**Table S1.** Crystal data and structure refinement for Mn{N(SiMe$_3$)Dipp}$_2$	
Identification code	GB136_MnN((Dipp)(TMS))2
Empirical formula	$C_{30}H_{52}MnN_2Si_2$
Formula weight	551.85
Temperature/K	123(2)
Crystal system	Triclinic
Space group	P-1
a/Å	9.5810(2)
b/Å	9.6720(2)
c/Å	17.8410(3)
α/°	95.935(2)
β/°	94.169(2)
γ/°	98.291(2)
Volume/Å^3	1620.77(6)
Z	2
ρ_{calc}g/cm^3	1.131
μ/mm^{-1}	0.501
F(000)	598.0
Crystal size/mm^3	0.632 × 0.395 × 0.286
Crystal colour	yellow
Radiation	Mo Kα (λ = 0.71073)
2Θ range for data collection/°	6.916 to 58.7
Index ranges	-13 ≤ h ≤ 13, -12 ≤ k ≤ 13, -24 ≤ l ≤ 24
Reflections collected	42670
Independent reflections	8003 [R_{int} = 0.0413, R_{sigma} = 0.0304]
Data/restraints/parameters	8003/0/333
Goodness-of-fit on F^2	1.053
Final R indexes [I>=2σ (I)]	R_1 = 0.0392, wR_2 = 0.1028
Final R indexes [all data]	R_1 = 0.0503, wR_2 = 0.1105
Largest diff. peak/hole / e Å^{-3}	0.36/-0.62

Crystal structure of [Na(^{n}Bu)Mn{N(SiMe$_3$)Dipp}$_2$(Et$_2$O)$_2$] (1)

A colourless crystal of [Na(^{n}Bu)Mn{N(SiMe$_3$)Dipp}$_2$(Et$_2$O)$_2$] (**1**) was embedded in inert perfluoropolyalkylether (viscosity 1800 cSt; ABCR GmbH) and mounted using a glass fiber. The crystal was then flash cooled to 123 K in a nitrogen gas stream and kept at this temperature during the experiment. The crystal structure was measured with an Oxford Diffraction Gemini E instrument with graphite-monochromated Mo−Kα (λ = 0.71073 Å) radiation. . The measured data was processed with the CrysAlisPro[13] software package. Using Olex2[14], the structure was solved with the ShelXT[15] structure solution program and refined to convergence against F^2 for all independent reflections. All non-hydrogen atoms were refined using anisotropic thermal parameters.

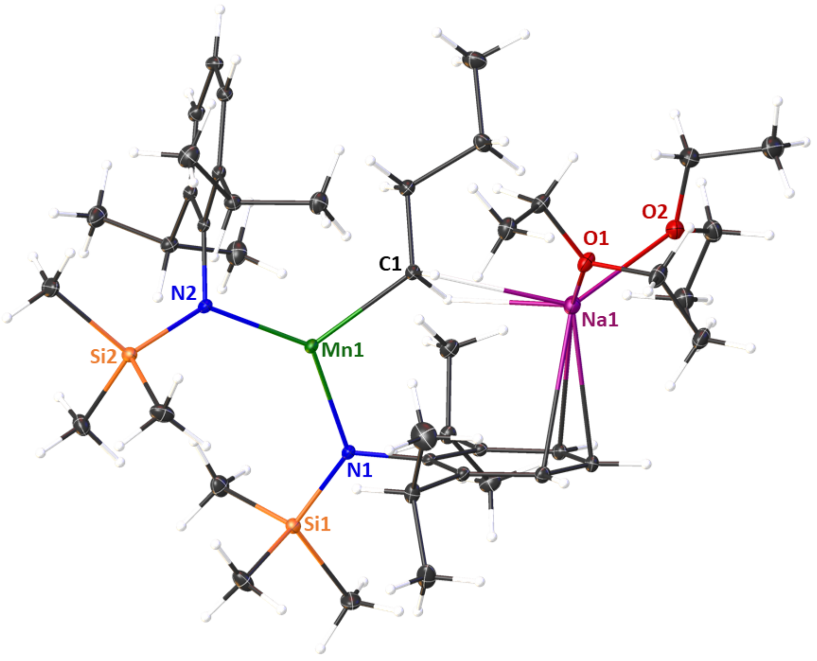

Figure S3. ORTEP plot of the asymmetric unit of [Na(Me$_3$SiCH$_2$)$_2$Mn{N(SiMe$_3$)Dipp}(Et$_2$O)$_2$] (**1**) (ellipsoids drawn at 30% probability).

**Table S2.** Crystal data and structure refinement for [Na(^{n}Bu)Mn{N(SiMe$_3$)Dipp}$_2$(Et$_2$O)$_2$] (**1**)	
Identification code	GB156_Et2O
Empirical formula	$MnNaSi_2O_2N_2C_{42}H_{81}$
Formula weight	780.19
Temperature/K	123(2)
Crystal system	Monoclinic
Space group	$P2_1/c$
a/Å	12.2582(1)
b/Å	21.8135(2)
c/Å	18.7285(2)
α/°	90
β/°	106.826(1)
γ/°	90

Volume/Å^3	4793.50(8)
Z	4
ρ_{calc}g/cm^3	1.081
μ/mm^{-1}	0.367
F(000)	1708.0
Crystal size/mm^3	0.75 × 0.40 × 0.28
Crystal colour	colourless
Radiation	Mo Kα (λ = 0.71073)
2Θ range for data collection/°	6.734 to 58.564
Index ranges	-16 ≤ h ≤ 16, -30 ≤ k ≤ 29, -25 ≤ l ≤ 25
Reflections collected	124709
Independent reflections	12343 [R_{int} = 0.0436, R_{sigma} = 0.0234]
Data/restraints/parameters	12343/0/478
Goodness-of-fit on F^2	1.025
Final R indexes [I>=2σ (I)]	R_1 = 0.0334, wR_2 = 0.0804
Final R indexes [all data]	R_1 = 0.0466, wR_2 = 0.0872
Largest diff. peak/hole / e Å^{-3}	0.46/-0.30

Crystal structure of ^{n}BuMn{N(SiMe$_3$)Dipp} (2a)

A colourless crystal of ^{n}BuMn{N(SiMe$_3$)Dipp} (**2a**) was embedded in inert perfluoropolyalkylether (viscosity 1800 cSt; ABCR GmbH) and mounted using a glass fiber. The crystal was then flash cooled to 123 K in a nitrogen gas stream and kept at this temperature during the experiment. The crystal structure was measured with an Oxford Diffraction Gemini E instrument with graphite-monochromated Mo−Kα (λ = 0.71073 Å) radiation. The measured data was processed with the CrysAlisPro[13] software package. Using Olex2[14], the structure was solved with the ShelXT[15] structure solution program and refined to convergence against F^2 for all independent reflections. All non-hydrogen atoms were refined using anisotropic thermal parameters.

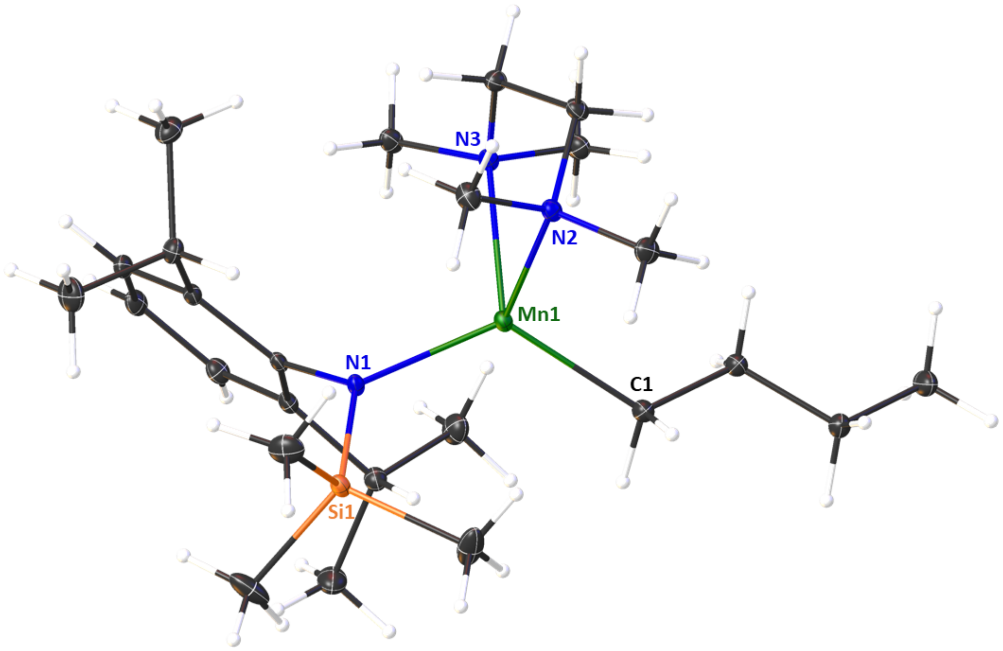

Figure S4. ORTEP plot of ^{n}BuMn{N(SiMe$_3$)Dipp} (**2a**) (ellipsoids drawn at 30% probability).

**Table S3.** Crystal data and structure refinement for ^{n}BuMn{N(SiMe$_3$)Dipp} (**2a**)	
Identification code	GB156_TMEDA
Empirical formula	$C_{25}H_{51}N_3SiMn$
Formula weight	476.71
Temperature/K	123(2)
Crystal system	Monoclinic
Space group	$P2_1/c$
a/Å	10.5532(2)
b/Å	13.8512(2)
c/Å	19.6317(3)
α/°	90
β/°	91.9230(10)
γ/°	90
Volume/Å^3	2868.04(8)

Z	4
ρ_{calc}g/cm^3	1.104
μ/mm^{-1}	0.517
F(000)	1044.0
Crystal size/mm^3	0.849 × 0.48 × 0.327
Crystal colour	colourless
Radiation	Mo Kα (λ = 0.71073)
2Θ range for data collection/°	6.89 to 58.682
Index ranges	-14 ≤ h ≤ 14, -18 ≤ k ≤ 19, -26 ≤ l ≤ 26
Reflections collected	61440
Independent reflections	7153 [R_{int} = 0.0513, R_{sigma} = 0.0260]
Data/restraints/parameters	7153/0/291
Goodness-of-fit on F^2	1.061
Final R indexes [I>=2σ (I)]	R_1 = 0.0395, wR_2 = 0.1072
Final R indexes [all data]	R_1 = 0.0483, wR_2 = 0.1141
Largest diff. peak/hole / e Å^{-3}	0.48/-0.54

Crystal structure of $Me_3SiCH_2Mn\{N(SiMe_3)Dipp\}$ (2b)

A colourless crystal of $Me_3SiCH_2Mn\{N(SiMe_3)Dipp\}$ (**2b**) was embedded in inert perfluoropolyalkylether (viscosity 1800 cSt; ABCR GmbH) and mounted using a glass fiber. The crystal was then flash cooled to 123 K in a nitrogen gas stream and kept at this temperature during the experiment. The crystal structure was measured with an Oxford Diffraction Gemini S instrument with graphite-monochromated Mo−Kα (λ = 0.71073 Å) radiation. The measured data was processed with the CrysAlisPro[13] software package. Using Olex2[14], the structure was solved with the ShelXT[15] structure solution program and refined to convergence against F^2 for all independent reflections. All non-hydrogen atoms were refined using anisotropic thermal parameters.

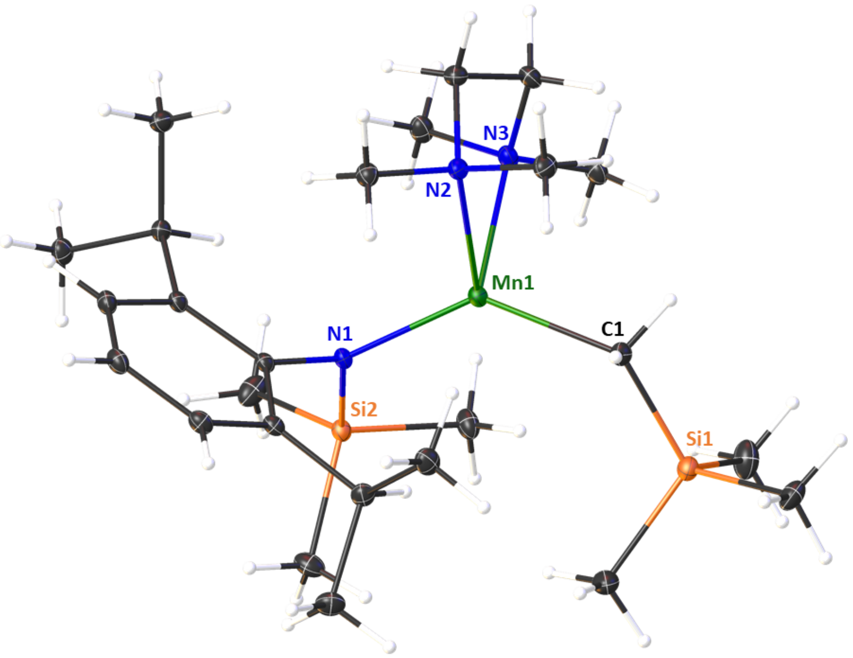

Figure S5. ORTEP plot of $Me_3SiCH_2Mn\{N(SiMe_3)Dipp\}$ (**2b**) (ellipsoids drawn at 30% probability).

**Table S4.** Crystal data and structure refinement for $Me_3SiCH_2Mn\{N(SiMe_3)Dipp\}$ (**2b**)	
Identification code	GB169_TMEDA
Empirical formula	$C_{25}H_{53}N_3Si_2Mn$
Formula weight	506.83
Temperature/K	123(2)
Crystal system	Monoclinic
Space group	$P2_1/n$
a/Å	9.3649(1)
b/Å	18.4575(2)
c/Å	17.8458(2)
α/°	90
β/°	99.669(1)
γ/°	90
Volume/Å^3	3040.87(6)

Z	4
ρ_{calc}g/cm^3	1.107
μ/mm^{-1}	0.529
F(000)	1108.0
Crystal size/mm^3	0.426 × 0.264 × 0.182
Crystal colour	colourless
Radiation	Mo Kα (λ = 0.71073)
2Θ range for data collection/°	6.912 to 58.596
Index ranges	-12 ≤ h ≤ 12, -24 ≤ k ≤ 24, -24 ≤ l ≤ 23
Reflections collected	78837
Independent reflections	7705 [R_{int} = 0.0554, R_{sigma} = 0.0307]
Data/restraints/parameters	7705/0/302
Goodness-of-fit on F^2	1.041
Final R indexes [I>=2σ (I)]	R_1 = 0.0433, wR_2 = 0.1113
Final R indexes [all data]	R_1 = 0.0592, wR_2 = 0.1231
Largest diff. peak/hole / e Å^{-3}	0.37/-0.48

Crystal structure of Rb{N($SiMe_3$)Dipp} (3-Rb)

A colourless crystal of Rb{N($SiMe_3$)Dipp} (**3-Rb**) was embedded in inert perfluoropolyalkylether (viscosity 1800 cSt; ABCR GmbH) and mounted using a glass fiber. The crystal was then flash cooled to 123 K in a nitrogen gas stream and kept at this temperature during the experiment. The crystal structure was measured with an Oxford Diffraction Gemini instrument with monochromated Cu−Kα (λ 1.54184 Å) radiation. The measured data was processed with the CrysAlisPro[13] software package. Using Olex2[14], the structure was solved with the ShelXT[15] structure solution program using and refined to convergence against *F*^{2} for all independent reflections. All non-hydrogen atoms were refined using anisotropic thermal parameters.

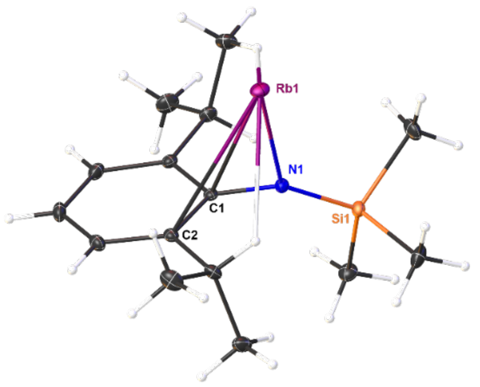

Figure S6. ORTEP plot of asymmetric unit of Rb{N(SiMe$_3$)Dipp} (**3-Rb**) (ellipsoids drawn at 30% probability).

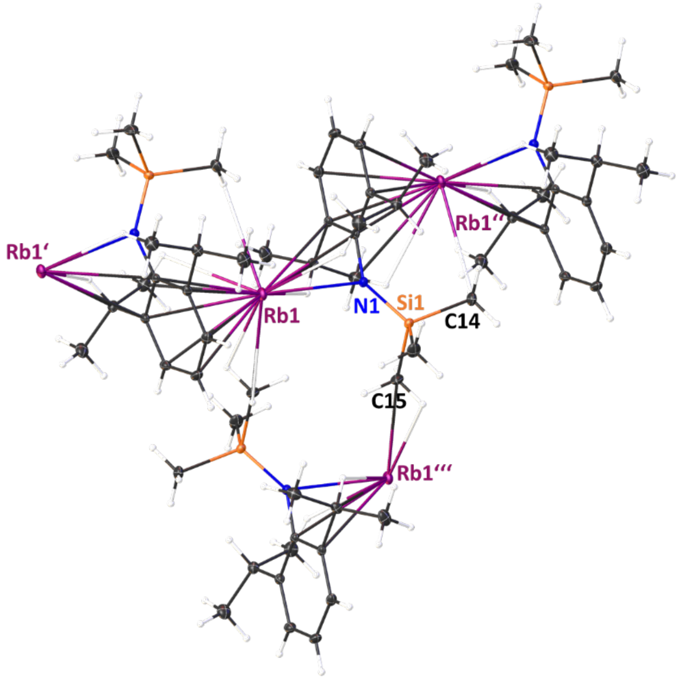

Figure S7. ORTEP plot of cut-out of polymeric structure of Rb{N(SiMe$_3$)Dipp} (**3-Rb**)(Ellipsoids drawn at 30% probability).

**Table S5.** Crystal data and structure refinement for Rb{N(SiMe$_3$)Dipp} (**3-Rb**)	
Identification code	GT_RbDIPPSiMe3
Empirical formula	$RbSiNC_{15}H_{26}$
Formula weight	333.93
Temperature/K	123(2)
Crystal system	Monoclinic
Space group	$P2_1/c$
a/Å	11.5274(1)
b/Å	13.6550(1)
c/Å	12.0556(1)
α/°	90
β/°	114.306(1)
γ/°	90

Volume/Å^3	1729.43(3)
Z	4
ρ_{calc}g/cm^3	1.283
μ/mm^{-1}	4.466
F(000)	696.0
Crystal size/mm^3	0.2 × 0.2 × 0.2
Crystal colour	Colourless
Radiation	Cu Kα (λ = 1.54184)
2Θ range for data collection/°	8.416 to 146.256
Index ranges	-14 ≤ h ≤ 14, -16 ≤ k ≤ 16, -14 ≤ l ≤ 14
Reflections collected	15222
Independent reflections	3428 [R_{int} = 0.0313, R_{sigma} = 0.0232]
Data/restraints/parameters	3428/0/170
Goodness-of-fit on F^2	1.042
Final R indexes [I>=2σ (I)]	R_1 = 0.0276, wR_2 = 0.0706
Final R indexes [all data]	R_1 = 0.0310, wR_2 = 0.0728
Largest diff. peak/hole / e Å^{-3}	0.44/-0.36

Crystal structure of Rb{N($SiMe_3$)Dipp}(TMEDA) (3-Rb·TMEDA)

A colourless crystal of Rb{N($SiMe_3$)Dipp}(TMEDA) (**3-Rb·TMEDA**) was embedded in inert perfluoropolyalkylether (viscosity 1800 cSt; ABCR GmbH) and mounted using a glass fiber. The crystal was then flash cooled to 100 K in a nitrogen gas stream and kept at this temperature during the experiment. The crystal structure was measured with a Rigaku XtaLAB Synergy-i with monochromated Cu−Kα (λ = 1.54184 Å) radiation. The measured data was processed with the CrysAlisPro[13] software package. Using Olex2[14], the structure was solved with the ShelXT[15] structure solution program and refined to convergence against F^2 for all independent reflections. The TMEDA ligand was modelled as disordered over two sites. Restraints and constraints were added to this disordered group to ensure

that geometry and displacement ellipsoids approximated normal behavior. The occupancies of the disordered sites refined to 84.0(6):16.0(6).https://chemistry-europe.onlinelibrary.wiley.com/doi/full/10.1002/cctc.202100218 - cctc202100218-bib-0053 All non-hydrogen atoms were refined using anisotropic thermal parameters.

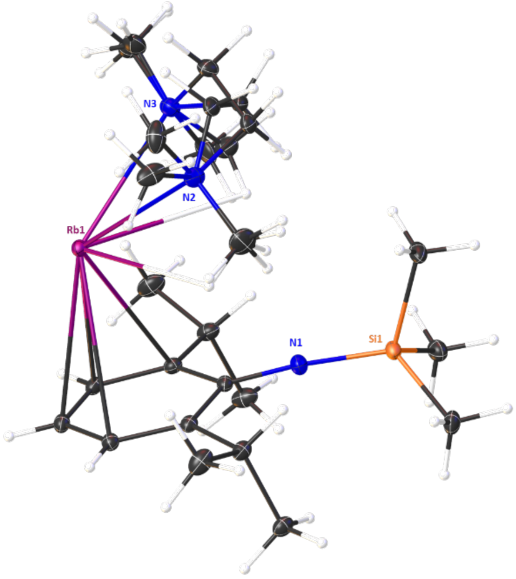

Figure S8. ORTEP plot of Rb{N(SiMe$_3$)Dipp}(TMEDA) (**3-Rb·TMEDA**) (ellipsoids drawn at 30% probability).

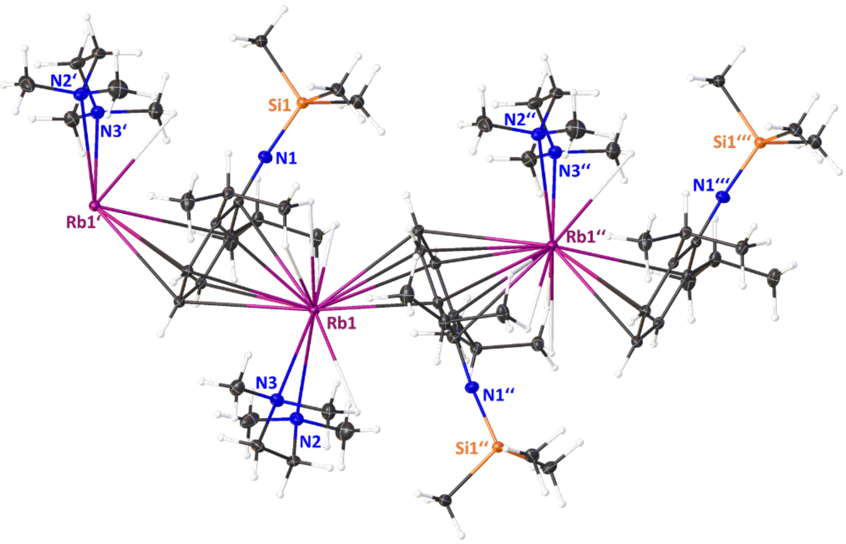

Figure S9. ORTEP plot of cut-out of infinite chain structure of Rb{N(SiMe$_3$)Dipp}(TMEDA) (**3-Rb·TMEDA**) (Ellipsoids drawn at 30% probability).

Table S6. Crystal data and structure refinement for Rb{N(SiMe$_3$)Dipp}(TMEDA) (**3-Rb·TMEDA**)

Identification code	GB_RbN_TMEDA
Empirical formula	$RbSiN_3C_{21}H_{42}$
Formula weight	450.13
Temperature/K	100(2)
Crystal system	Monoclinic
Space group	C2/c
a/Å	20.8411(2)
b/Å	11.6104(1)
c/Å	21.5822(2)
α/°	90
β/°	103.755(1)
γ/°	90
Volume/Å^3	5072.55(8)
Z	8
ρ_{calc}g/cm^3	1.179
μ/mm^{-1}	3.186
F(000)	1920.0
Crystal size/mm^3	0.452 × 0.076 × 0.054
Crystal colour	Colourless
Radiation	Cu Kα (λ = 1.54184)
2Θ range for data collection/°	8.436 to 145.24
Index ranges	-25 ≤ h ≤ 25, -14 ≤ k ≤ 9, -26 ≤ l ≤ 26
Reflections collected	26856
Independent reflections	5027 [R_{int} = 0.0371, R_{sigma} = 0.0266]
Data/restraints/parameters	5027/116/339
Goodness-of-fit on F^2	1.059
Final R indexes [I>=2σ (I)]	R_1 = 0.0306, wR_2 = 0.0855
Final R indexes [all data]	R_1 = 0.0330, wR_2 = 0.0872
Largest diff. peak/hole / e Å^{-3}	0.69/-0.46

Crystal structure of Rb{N(SiMe$_3$)Dipp}(PMDETA) (3-Rb·PMDETA)

A colourless crystal of Rb{N(SiMe$_3$)Dipp}(PMDETA) (**3-Rb·PMDETA**) was embedded in inert perfluoropolyalkylether (viscosity 1800 cSt; ABCR GmbH) and mounted using a glass fiber. The crystal was then flash cooled to 100 K in a nitrogen gas stream and kept at this temperature during the experiment. The crystal structure was measured with a Rigaku XtaLAB Synergy-i with monochromated Cu−Kα (λ 1.54184 Å) radiation. The measured data was processed with the CrysAlisPro[13] software package. Using Olex2[14], the structure was solved with the ShelXT[15] structure solution program and refined to convergence against F^2 for all independent reflections by the full-matrix least-squares. All non-hydrogen atoms were refined using anisotropic thermal parameters.

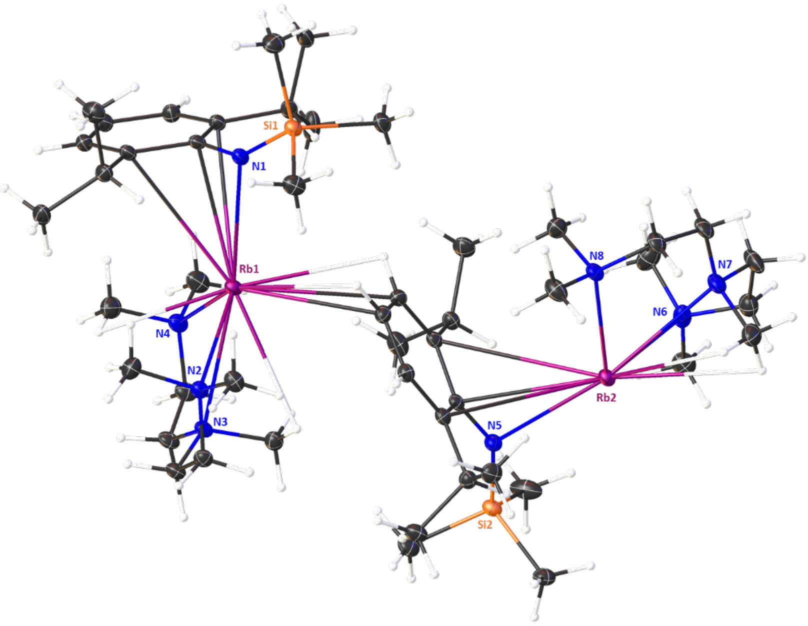

Figure S10. ORTEP plot of Rb{N(SiMe$_3$)Dipp}(PMDETA) (**3-Rb·PMDETA**) (ellipsoids drawn at 30% probability).

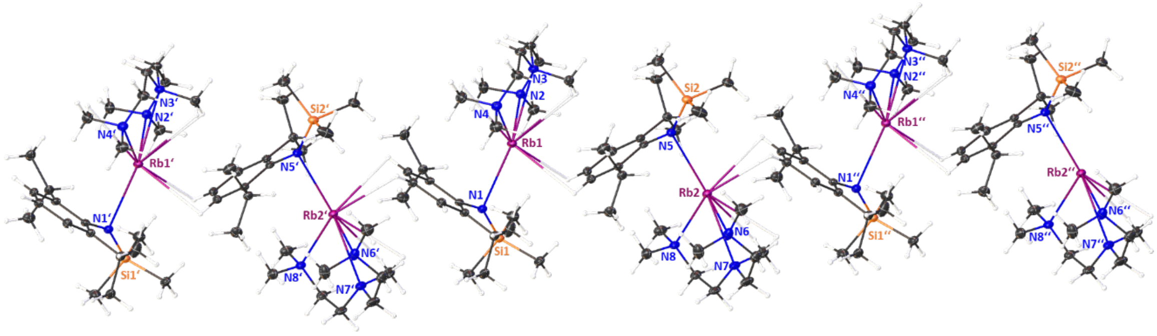

Figure S11. ORTEP plot of cut-out of infinite chain structure of Rb{N(SiMe$_3$)Dipp}(PMDETA) (**3-Rb·PMDETA**) (Ellipsoids drawn at 30% probability).

**Table S6.** Crystal data and structure refinement for Rb{N(SiMe$_3$)Dipp}(PMDETA) **3-Rb·PMDETA**)	
Identification code	GB_RbN_PMDETA
Empirical formula	$RbSiN_4C_{24}H_{49}$
Formula weight	507.23
Temperature/K	100(2)
Crystal system	Monoclinic
Space group	$P2_1/c$
a/Å	14.6139(1)
b/Å	21.5364(2)
c/Å	18.7195(2)
α/°	90
β/°	97.256(1)
γ/°	90

Volume/Å^3	5844.42(9)
Z	8
ρ_{calc}g/cm^3	1.153
μ/mm^{-1}	2.827
F(000)	2176.0
Crystal size/mm^3	0.1 × 0.1 × 0.1
Crystal colour	Colourless
Radiation	Cu Kα (λ = 1.54184)
2Θ range for data collection/°	6.096 to 145.384
Index ranges	-17 ≤ h ≤ 14, -26 ≤ k ≤ 23, -23 ≤ l ≤ 23
Reflections collected	64319
Independent reflections	11561 [R_{int} = 0.0743, R_{sigma} = 0.0435]
Data/restraints/parameters	11561/0/565
Goodness-of-fit on F^2	1.063
Final R indexes [I>=2σ (I)]	R_1 = 0.0500, wR_2 = 0.1401
Final R indexes [all data]	R_1 = 0.0578, wR_2 = 0.1464
Largest diff. peak/hole / e Å^{-3}	0.83/-1.11

Crystal structure of Cs{N($SiMe_3$)Dipp} (3-Cs)

A colourless crystal of Cs{N($SiMe_3$)Dipp} (**3-Cs**) was embedded in inert perfluoropolyalkylether (viscosity 1800 cSt; ABCR GmbH) and mounted using a glass fiber. The crystal was then flash cooled to 123 K in a nitrogen gas stream and kept at this temperature during the experiment. The crystal structure was measured with an Oxford Diffraction Gemini diffractometer with monochromated Mo−Kα (λ 0.71073 Å) radiation. The measured data was processed with the CrysAlisPro[13] software package. Using Olex2[14], the structure was solved with the ShelXT[15] structure solution program and refined to convergence against F^2 for all independent reflections by the full-matrix least-squares method. All non-hydrogen atoms were refined using anisotropic thermal parameters.

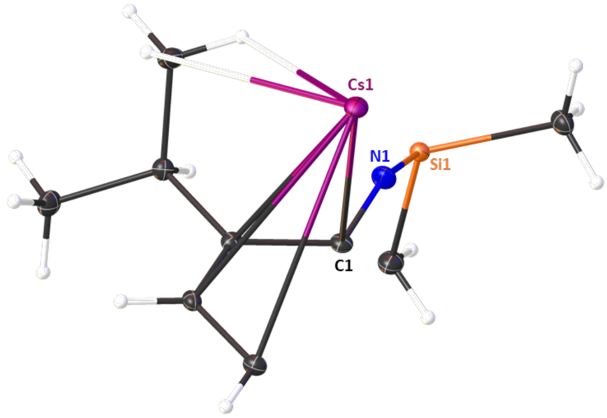

Figure S12. ORTEP plot of asymmetric unit of Cs{N(SiMe$_3$)Dipp} (**3-Cs**) (ellipsoids drawn at 30% probability).

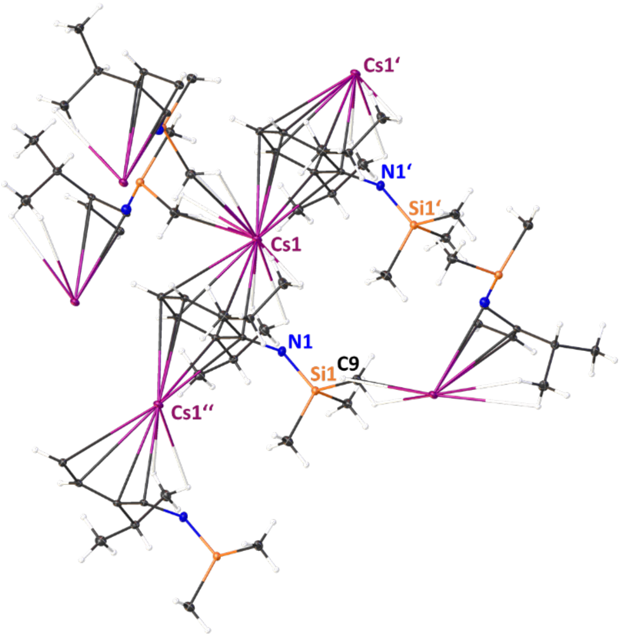

Figure S13. ORTEP plot of part of the polymeric structure of Cs{N(SiMe$_3$)Dipp} (**3-Cs**) (Ellipsoids drawn at 30% probability).

**Table S7.** Crystal data and structure refinement for Cs{N(SiMe$_3$)Dipp} (**3-Cs**)	
Identification code	GT_153_xtals
Empirical formula	$CsSiNC_{15}H_{26}$
Formula weight	381.37
Temperature/K	123(2)
Crystal system	Tetragonal
Space group	P-42$_1$m
a/Å	16.5323(2)
b/Å	16.5323(2)
c/Å	6.30490(1)
α/°	90
β/°	90
γ/°	90

Volume/Å^3	1723.24(5)
Z	4
ρ_{calc}g/cm^3	1.470
μ/mm^{-1}	2.205
F(000)	768.0
Crystal size/mm^3	0.36 × 0.25 × 0.2
Crystal colour	Colourless
Radiation	Mo Kα (λ = 0.71073)
2Θ range for data collection/°	6.916 to 58.448
Index ranges	-20 ≤ h ≤ 21, -20 ≤ k ≤ 22, -7 ≤ l ≤ 8
Reflections collected	10478
Independent reflections	2243 [R_{int} = 0.0374, R_{sigma} = 0.0305]
Data/restraints/parameters	2243/0/145
Goodness-of-fit on F^2	1.043
Final R indexes [I>=2σ (I)]	R_1 = 0.0256, wR_2 = 0.0570
Final R indexes [all data]	R_1 = 0.0283, wR_2 = 0.0587
Largest diff. peak/hole / e Å^{-3}	0.42/-0.65
Flack parameter	-0.018(18)

Crystal structure of Cs{N($SiMe_3$)Dipp}(TMEDA) (3-Cs·TMEDA)

A colourless crystal of Cs{N($SiMe_3$)Dipp}(TMEDA) (**3-Cs·TMEDA**) was embedded in inert perfluoropolyalkylether (viscosity 1800 cSt; ABCR GmbH) and mounted using a glass fiber. The crystal was then flash cooled to 100 K in a nitrogen gas stream and kept at this temperature during the experiment. The crystal structure was measured with a Rigaku XtaLAB Synergy-i with monochromated Cu−Kα (λ 1.54184 Å) radiation. The measured data was processed with the CrysAlisPro[13] software package. Using Olex2[14], the structure was solved with the ShelXT[15] structure solution program and

refined to convergence against F^2 for all independent reflections. All non-hydrogen atoms were refined using anisotropic thermal parameters.

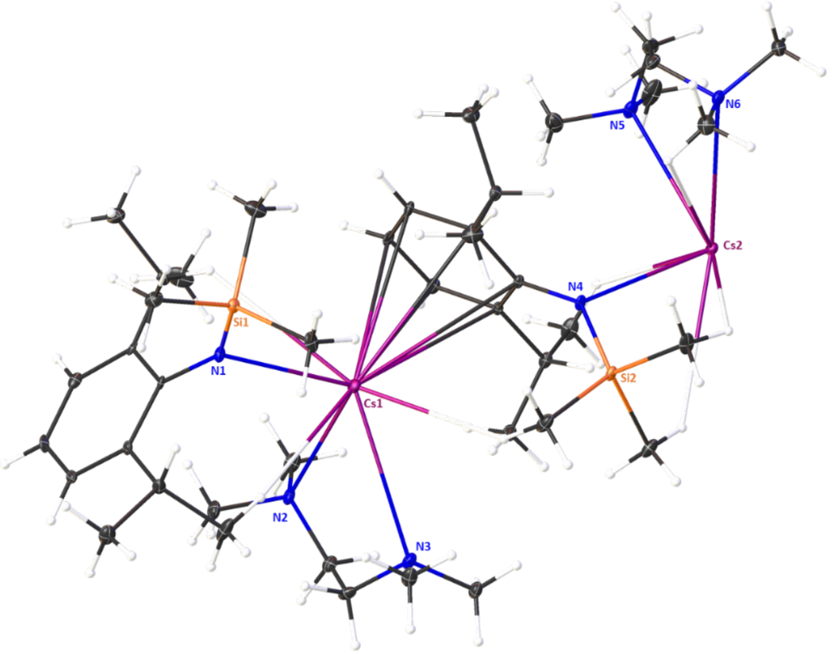

Figure S14. ORTEP plot of Cs{N(SiMe$_3$)Dipp}(TMEDA) (**3-Cs·TMEDA**) (ellipsoids drawn at 30% probability).

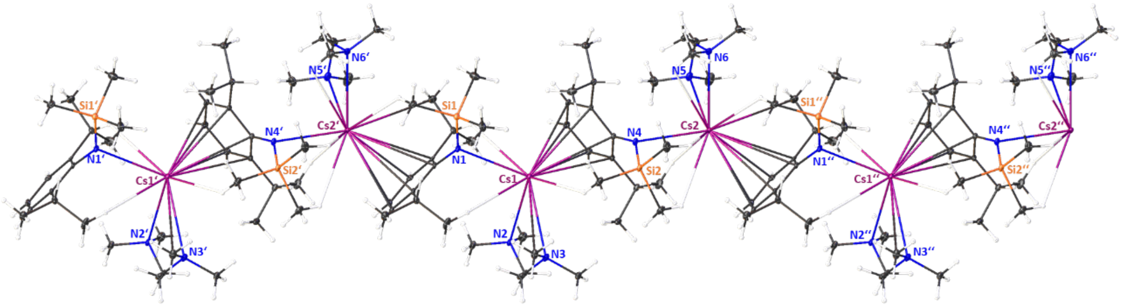

Figure S15. ORTEP plot of cut-out of infinite chain structure of Cs{N(SiMe$_3$)Dipp}(TMEDA) (**3-Cs·TMEDA**) (Hydrogen atoms omitted for clarity, ellipsoids drawn at 30% probability).

**Table S8.** Crystal data and structure refinement for Cs{N(SiMe$_3$)Dipp}(TMEDA) (**3-Cs·TMEDA**)	
Identification code	GB178_TMEDA_Hex
Empirical formula	$C_{42}H_{84}Cs_2N_6Si_2$
Formula weight	995.15
Temperature/K	100(2)
Crystal system	Monoclinic
Space group	$P2_1/c$
a/Å	14.3023(3)
b/Å	18.1906(3)

c/Å	19.5502(3)
α/°	90
β/°	92.413(2)
γ/°	90
Volume/Å^3	5081.82(16)
Z	4
ρ_{calc}g/cm^3	1.301
μ/mm^{-1}	11.870
F(000)	2064.0
Crystal size/mm^3	0.338 × 0.322 × 0.215
Crystal colour	colourless
Radiation	Cu Kα (λ = 1.54184)
2Θ range for data collection/°	6.186 to 145.974
Index ranges	-17 ≤ h ≤ 17, -22 ≤ k ≤ 22, -24 ≤ l ≤ 23
Reflections collected	78840
Independent reflections	10079 [R_{int} = 0.1425, R_{sigma} = 0.0559]
Data/restraints/parameters	10079/0/491
Goodness-of-fit on F^2	1.099
Final R indexes [I>=2σ (I)]	R_1 = 0.0725, wR_2 = 0.1927
Final R indexes [all data]	R_1 = 0.0766, wR_2 = 0.1954
Largest diff. peak/hole / e Å^{-3}	2.80/-2.51

Crystal structure of Cs{N($SiMe_3$)Dipp}(PMDETA) (3-Cs·PMDETA)

A colourless crystal of Cs{N($SiMe_3$)Dipp}(PMDETA) (**3-Cs·PMDETA**) was embedded in inert perfluoropolyalkylether (viscosity 1800 cSt; ABCR GmbH) and mounted using a glass fiber. The crystal was then flash cooled to 100 K in a nitrogen gas stream and kept at this temperature during the experiment. The crystal structure was measured with a Rigaku XtaLAB Synergy-i diffractometer with monochromated Cu−Kα (λ 1.54184 Å) radiation. The measured data was processed with the

CrysAlisPro[13] software package. Using Olex2[14], the structure was solved with the ShelXT[15] structure solution program and refined to convergence against F^2 for all independent reflections. One Me_3Si-group was modelled over two disordered positions, with an approximate ratio of 80(1):20(1). For this group suitable constrains and restraints were applied such that normal geometries and displacement parameters were approximated. All non-hydrogen atoms were refined using anisotropic thermal parameters.

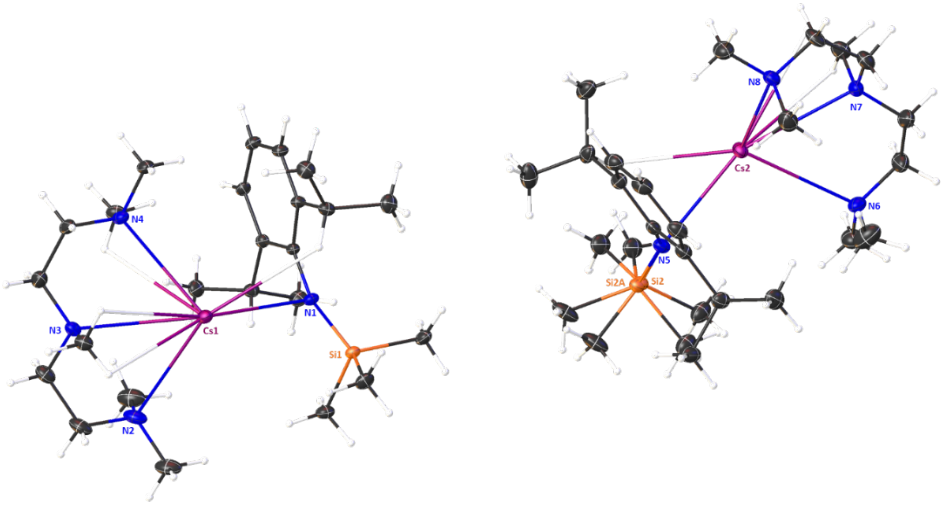

Figure S16. ORTEP plot of Cs{N($SiMe_3$)Dipp}(PMDETA) (**3-Cs·PMDETA**) (ellipsoids drawn at 30% probability).

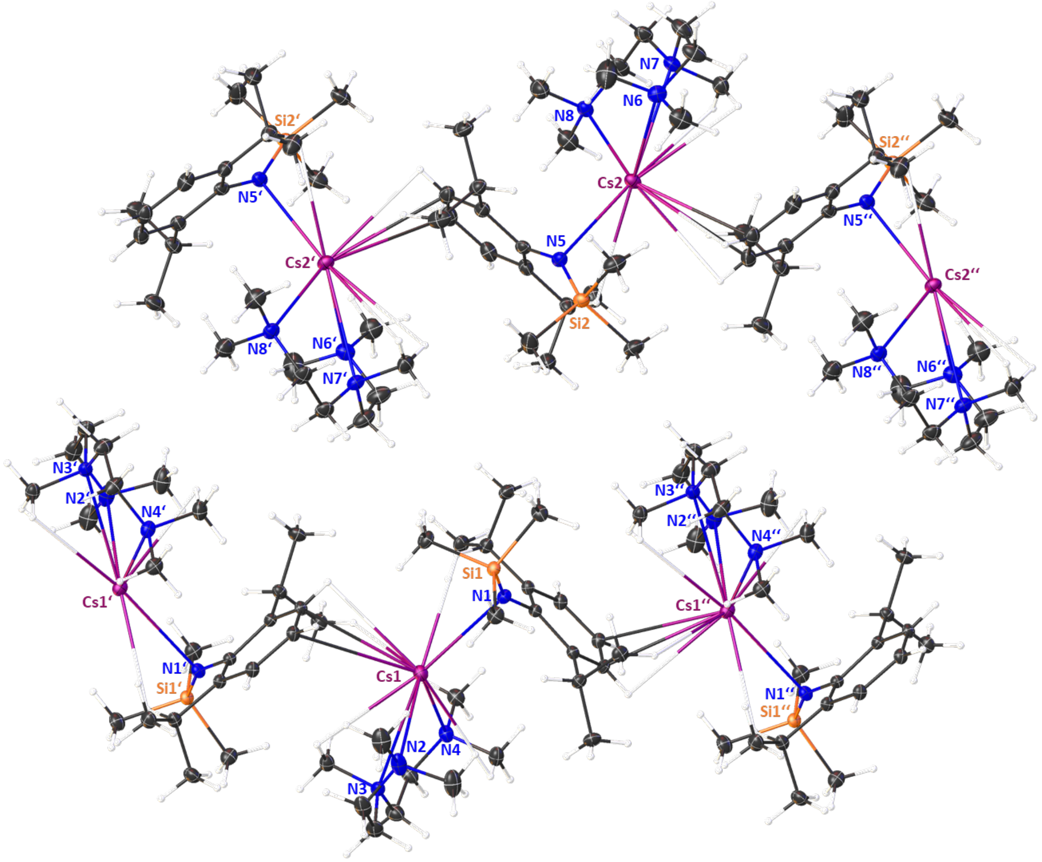

Figure S17. ORTEP plot of cut-out of infinite chain structure of Cs{N(SiMe$_3$)Dipp}(PMDETA) (**3-Cs·PMDETA**) (Hydrogen atoms omitted for clarity, ellipsoids drawn at 30% probability).

**Table S9.** Crystal data and structure refinement for CsMn{N(SiMe$_3$)Dipp}(PMDETA) (**3-Cs·PMDETA**)	
Identification code	GB_CsN_PMDETA
Empirical formula	$CsSiN_4C_{24}H_{49}$
Formula weight	554.67
Temperature/K	100(2)
Crystal system	Monoclinic
Space group	$P2_1/c$
a/Å	21.1596(6)
b/Å	15.4370(3)
c/Å	19.8879(4)
α/°	90
β/°	113.294(3)
γ/°	90
Volume/Å^3	5966.7(3)
Z	8
ρ_{calc}g/cm^3	1.235
μ/mm^{-1}	10.170
F(000)	2320.0
Crystal size/mm^3	0.334 × 0.153 × 0.111
Crystal colour	colourless
Radiation	Cu Kα (λ = 1.54184)
2Θ range for data collection/°	7.314 to 145.8
Index ranges	-26 ≤ h ≤ 25, -18 ≤ k ≤ 13, -24 ≤ l ≤ 24
Reflections collected	61880
Independent reflections	11799 [R_{int} = 0.0649, R_{sigma} = 0.0508]
Data/restraints/parameters	11799/8/581
Goodness-of-fit on F^2	1.105
Final R indexes [I>=2σ (I)]	R_1 = 0.0538, wR_2 = 0.1379
Final R indexes [all data]	R_1 = 0.0733, wR_2 = 0.1479
Largest diff. peak/hole / e Å^{-3}	1.04/-1.81

Crystal structure of [Rb(Me_3SiCH_2)Mn{N($SiMe_3$)Dipp}$_2$] (4-b)

A yellow crystal of [Rb(Me_3SiCH_2)Mn{N($SiMe_3$)Dipp}$_2$] (**4-Rb**) was embedded in inert perfluoropolyalkylether (viscosity 1800 cSt; ABCR GmbH) and mounted using a glass fiber. The crystal was then flash cooled to 100 K in a nitrogen gas stream and kept at this temperature during the experiment. The crystal structure was measured with a Rigaku XtaLAB Synergy-i diffractometer with monochromated Cu−Kα (λ = 1.54184 Å) radiation. The measured data was processed with the CrysAlisPro[13] software package. Using Olex2[14], the structure was solved with the ShelXT[15] structure solution program and refined to convergence against F^2 for all independent reflections. All non-hydrogen atoms were refined using anisotropic thermal parameters. The molecule of co-complexed *n*-hexane was modelled with the help of rigid-bond restraint (DELU) and similar-bond restraints (SIMU).

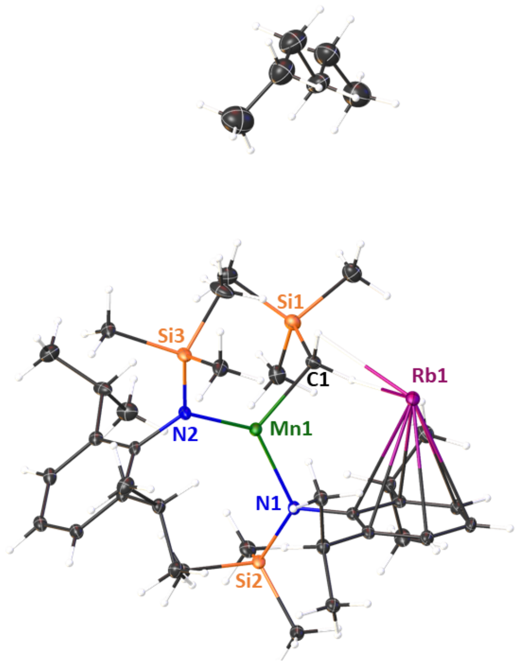

Figure S18. ORTEP plot of asymmetric unit of [Rb(Me_3SiCH_2)Mn{N($SiMe_3$)Dipp}$_2$] (**4-Rb**) (ellipsoids drawn at 30% probability).

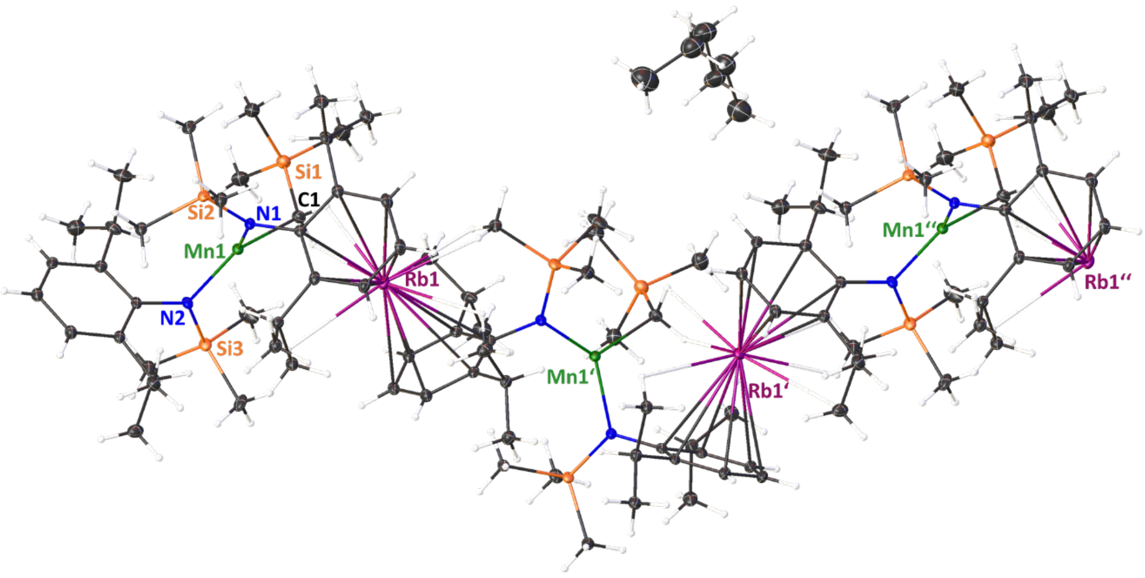

Figure S19. ORTEP plot of cut-out of infinite zig-zag chain structure of [Rb(Me_3SiCH_2)Mn{N($SiMe_3$)Dipp}$_2$] (**4-Rb**) (Elipsoids drawn at 30% probability).

**Table S10.** Crystal data and structure refinement for [Rb(Me_3SiCH_2)Mn{N($SiMe_3$)Dipp}$_2$] (**4-Rb**)	
Identification code	GB175_RbMnRN2_Tol_Hex
Empirical formula	$MnRbSi_3N_2C_{40}H_{77}$
Formula weight	810.71
Temperature/K	100(2)
Crystal system	Monoclinic
Space group	$P2_1/c$
a/Å	21.9305(8)
b/Å	11.8480(3)
c/Å	18.5066(6)
α/°	90
β/°	108.448(4)
γ/°	90
Volume/Å^3	4561.5(3)
Z	4
ρ_{calc}g/cm^3	1.181
μ/mm^{-1}	4.586
F(000)	1740.0
Crystal size/mm^3	0.273 × 0.186 × 0.077
Crystal colour	Yellow
Radiation	CuKα (λ = 1.54184)
2Θ range for data collection/°	8.5 to 139.926
Index ranges	-26 ≤ h ≤ 26, -13 ≤ k ≤ 14, -22 ≤ l ≤ 22
Reflections collected	50651
Independent reflections	8650 [R_{int} = 0.0821, R_{sigma} = 0.0495]
Data/restraints/parameters	8650/44/443
Goodness-of-fit on F^2	1.024
Final R indexes [I>=2σ (I)]	R_1 = 0.0851, wR_2 = 0.2254
Final R indexes [all data]	R_1 = 0.1004, wR_2 = 0.2393
Largest diff. peak/hole / e Å^{-3}	2.98/-0.89

Crystal structure of $[Cs(Me_3SiCH_2)Mn\{N(SiMe_3)Dipp\}_2]$ (4-Cs)

A yellow crystal of $[Cs(Me_3SiCH_2)Mn\{N(SiMe_3)Dipp\}_2]$ (**4-Cs**) was embedded in inert perfluoropolyalkylether (viscosity 1800 cSt; ABCR GmbH) and mounted using a glass fiber. The crystal was then flash cooled to 100 K in a nitrogen gas stream and kept at this temperature during the experiment. The crystal structure was measured with a Rigaku XtaLAB Synergy-i diffractometer with monochromated Cu−Kα (λ = 1.54184 Å) radiation. The measured data was processed with the CrysAlisPro[13] software package. Using Olex2[14], the structure was solved with the ShelXT[15] structure solution program and refined to convergence against F^2 for all independent reflections. All non-hydrogen atoms were refined using anisotropic thermal parameters.

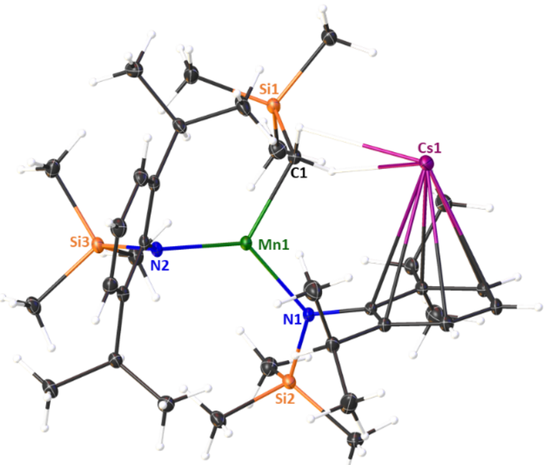

Figure S20. ORTEP plot of asymmetric unit of $[Cs(Me_3SiCH_2)Mn\{N(SiMe_3)Dipp\}_2]$ (**4-Cs**) (ellipsoids drawn at 30% probability).

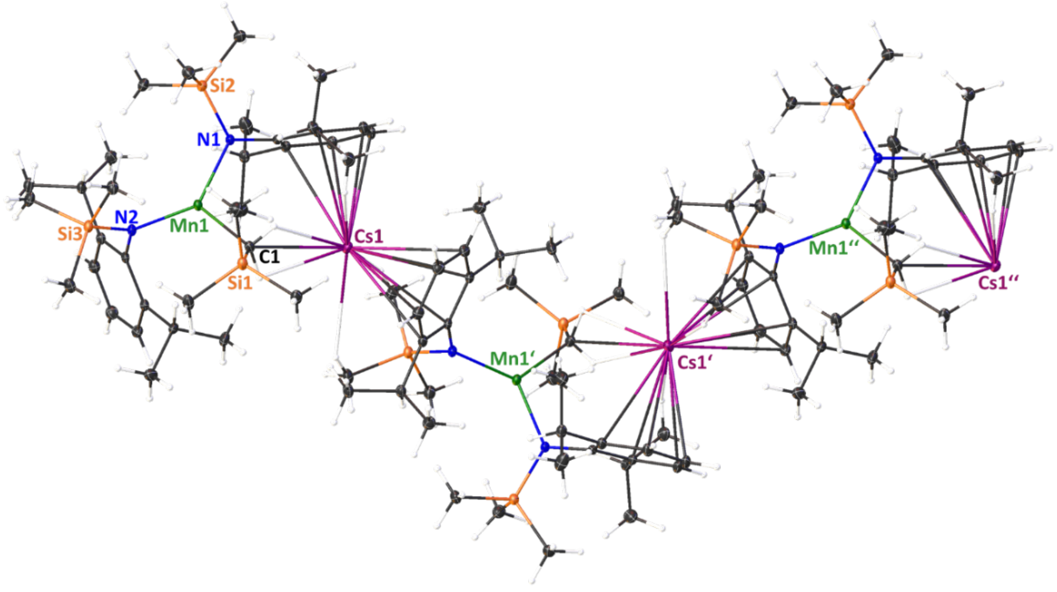

Figure S21. ORTEP plot of cut-out of infinite zig-zag chain structure of $[Cs(Me_3SiCH_2)Mn\{N(SiMe_3)Dipp\}_2]$ (**4-Cs**) (Ellipsoids drawn at 30% probability).

**Table S11.** Crystal data and structure refinement for [Cs(Me_3SiCH_2)Mn{N($SiMe_3$)Dipp}$_2$] (**4-Cs**)	
Identification code	GB178_Tol_Hex
Empirical formula	$MnCsSi_3N_2C_{34}H_{63}$
Formula weight	771.98
Temperature/K	100(2)
Crystal system	Monoclinic
Space group	$P2_1/n$
a/Å	11.45798(10)
b/Å	18.09796(10)
c/Å	19.96565(19)
α/°	90
β/°	102.2574(9)
γ/°	90
Volume/Å^3	4561.5(3)
Z	4
ρ_{calc}g/cm^3	1.267
μ/mm^{-1}	10.574
F(000)	1612.0
Crystal size/mm^3	0.524 × 0.325 × 0.253
Crystal colour	Yellow
Radiation	CuKα (λ = 1.54184)
2Θ range for data collection/°	6.662 to 145.918
Index ranges	-14 ≤ h ≤ 14, -18 ≤ k ≤ 22, -24 ≤ l ≤ 24
Reflections collected	84174
Independent reflections	8023 [R_{int} = 0.0858, R_{sigma} = 0.0298]
Data/restraints/parameters	8023/0/395
Goodness-of-fit on F^2	1.029
Final R indexes [I>=2σ (I)]	R_1 = 0.0495, wR_2 = 0.1343
Final R indexes [all data]	R_1 = 0.0507, wR_2 = 0.1355
Largest diff. peak/hole / e Å^{-3}	2.17/-0.99

3) Selected ^{1}H, ^{13}C, DEPT-135, DEPT-90, $^{1}H^{1}H$-COSY, $^{1}H^{13}C$-HSQC, $^{1}H^{13}C$-HMBC, ^{29}Si and paramagnetic NMR spectra

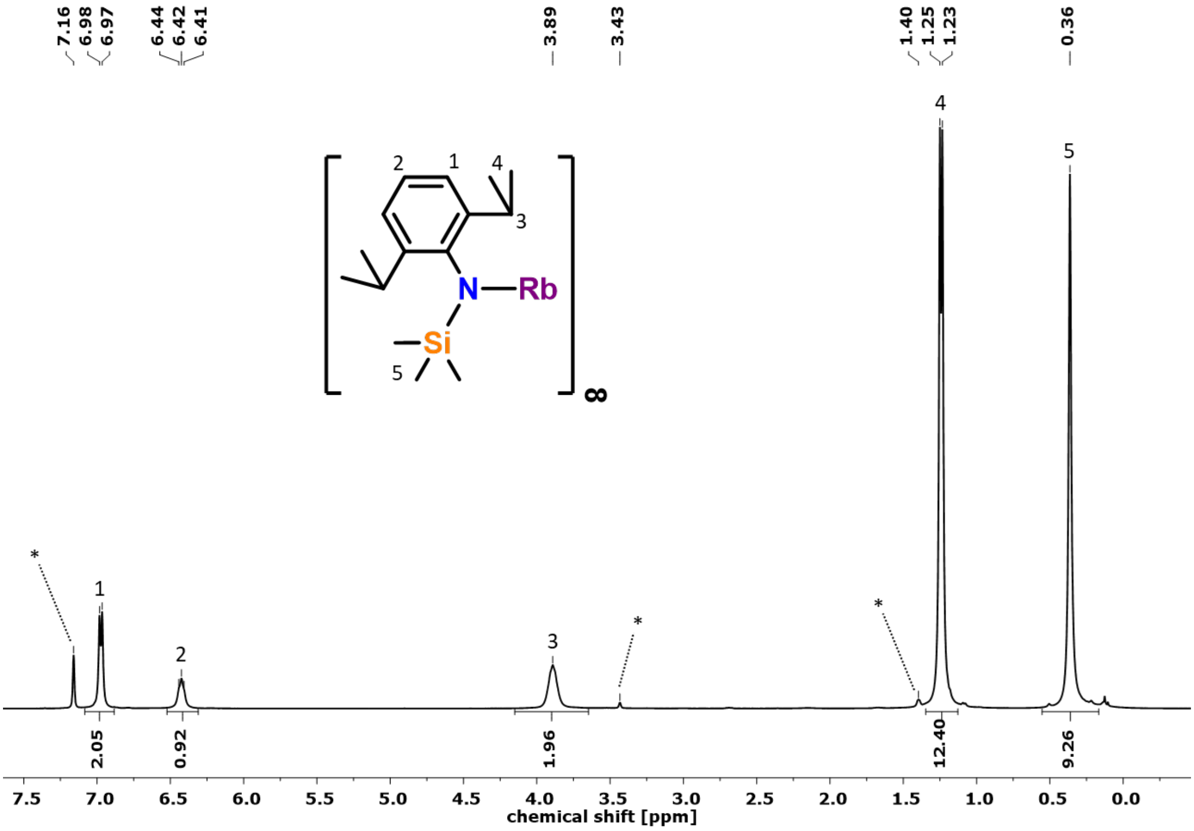

Figure S22. ^{1}H NMR spectrum of [{Rb(N$^{`Ar}$)}$_n$] in a mixture of benzene-d_6:THF-$d8$ (10:1). Deuterated solvents marked with *.

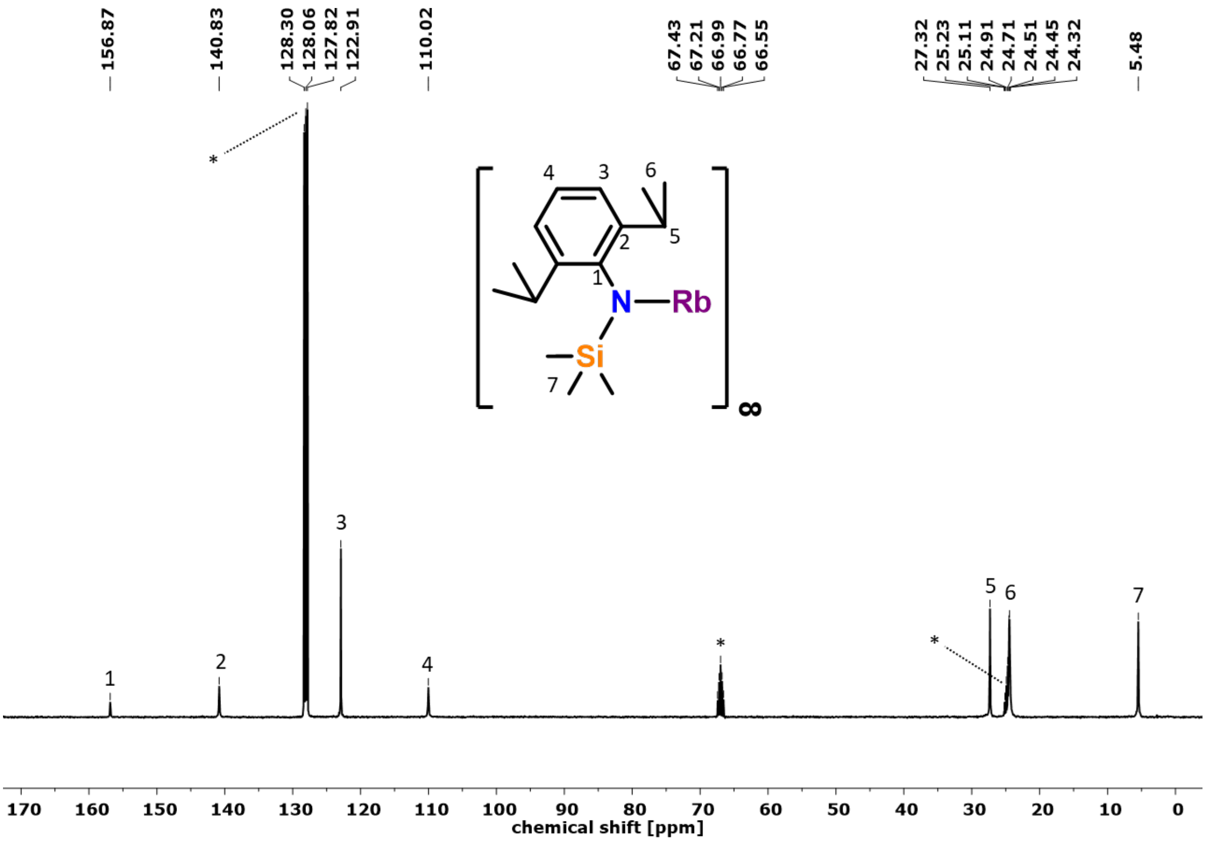

Figure S23. ^{13}C NMR spectrum of [{Rb(N$^{`Ar}$)}$_n$] in a mixture of benzene-d_6:THF-*d8* (10:1). Deuterated solvents marked with *.

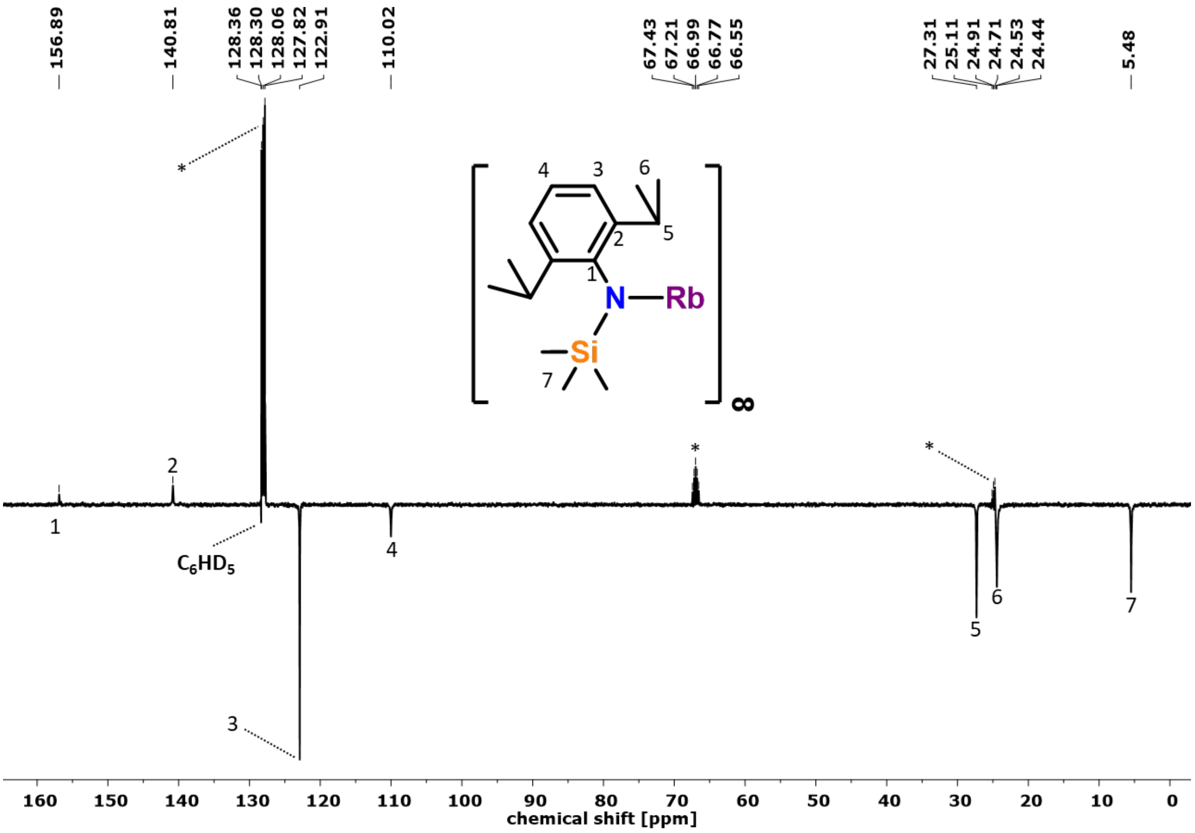

Figure S24. ^{13}C-DEPT-135 NMR spectrum of [{Rb(N$^{`Ar}$)}$_n$] in a mixture of benzene-d_6:THF-*d8* (10:1). Deuterated solvents marked with *.

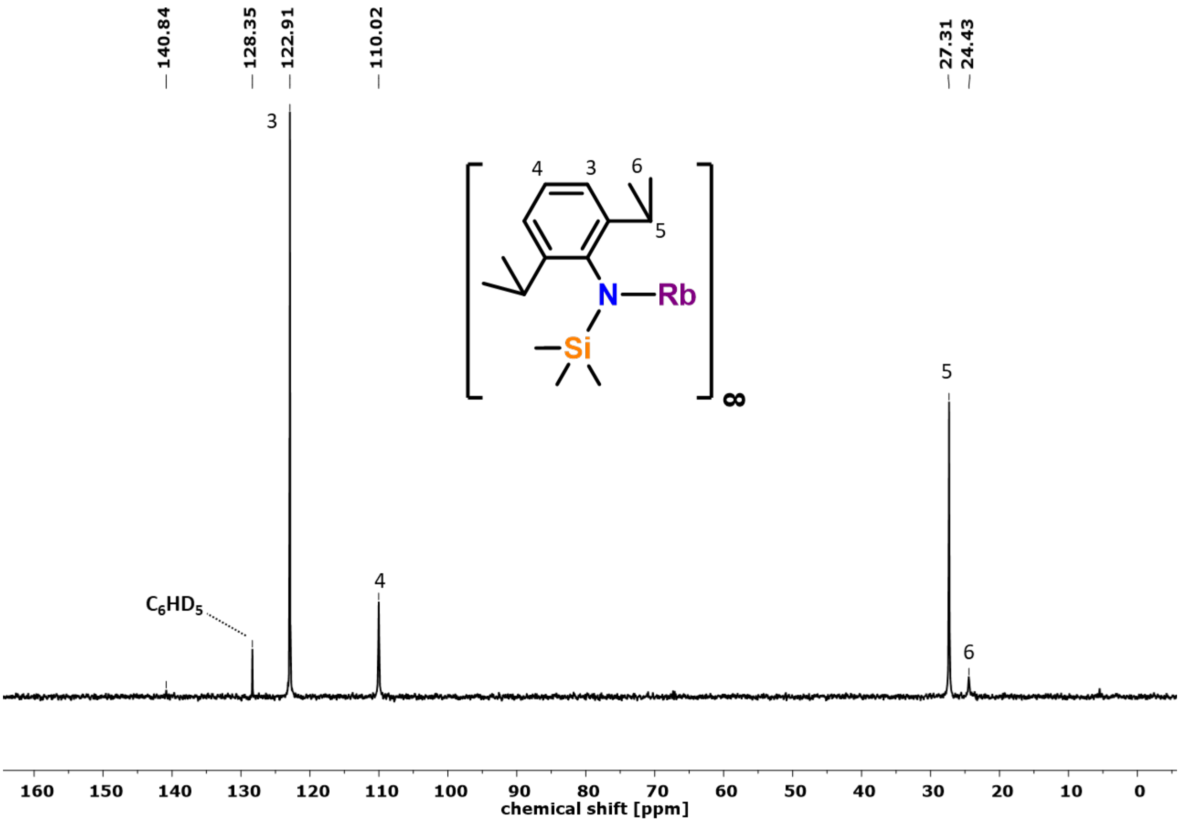

Figure S25. ^{13}C-DEPT-90 NMR spectrum of [{Rb(N$^{`Ar}$)}$_n$] in a mixture of benzene-d_6:THF-*d8* (10:1).

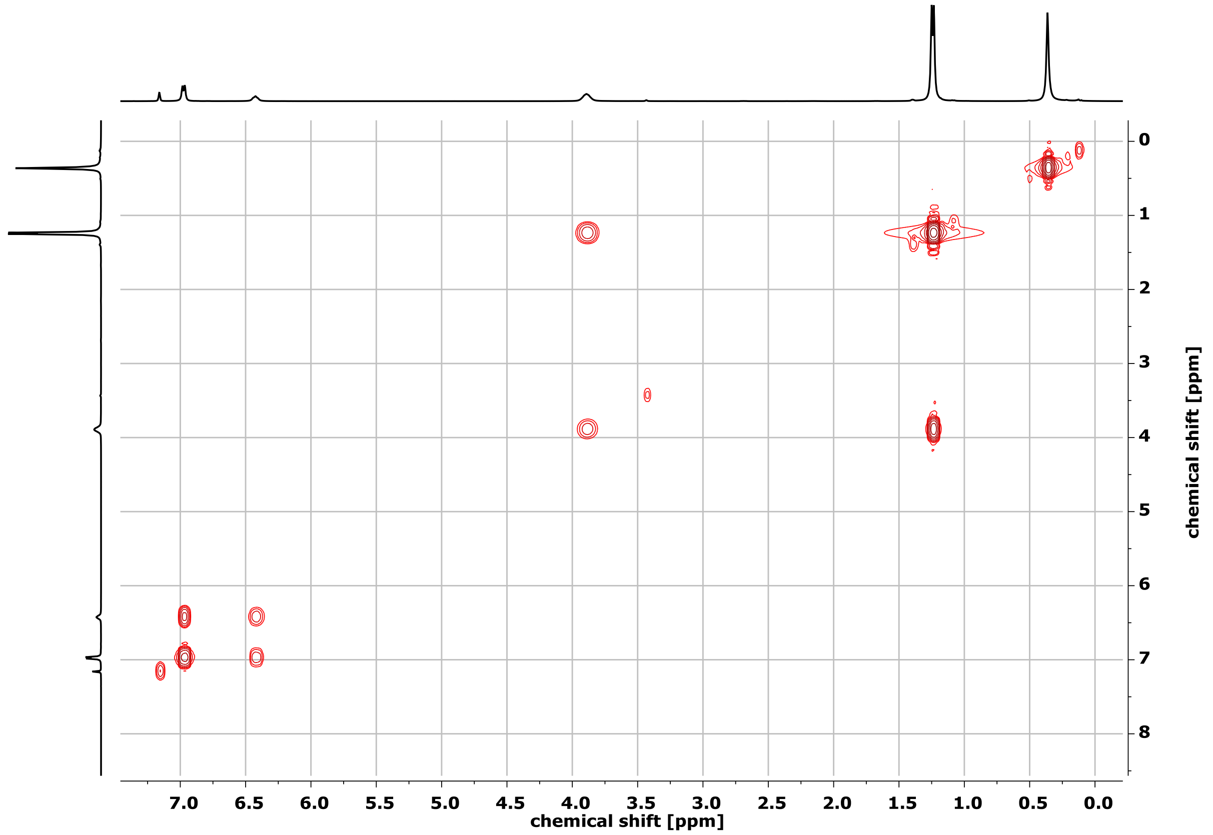

Figure S26. ^{1}H^{1}H-COSY NMR spectrum of [{Rb(N$^{`Ar}$)}$_n$] in a mixture of benzene-d_6:THF-*d8* (10:1).

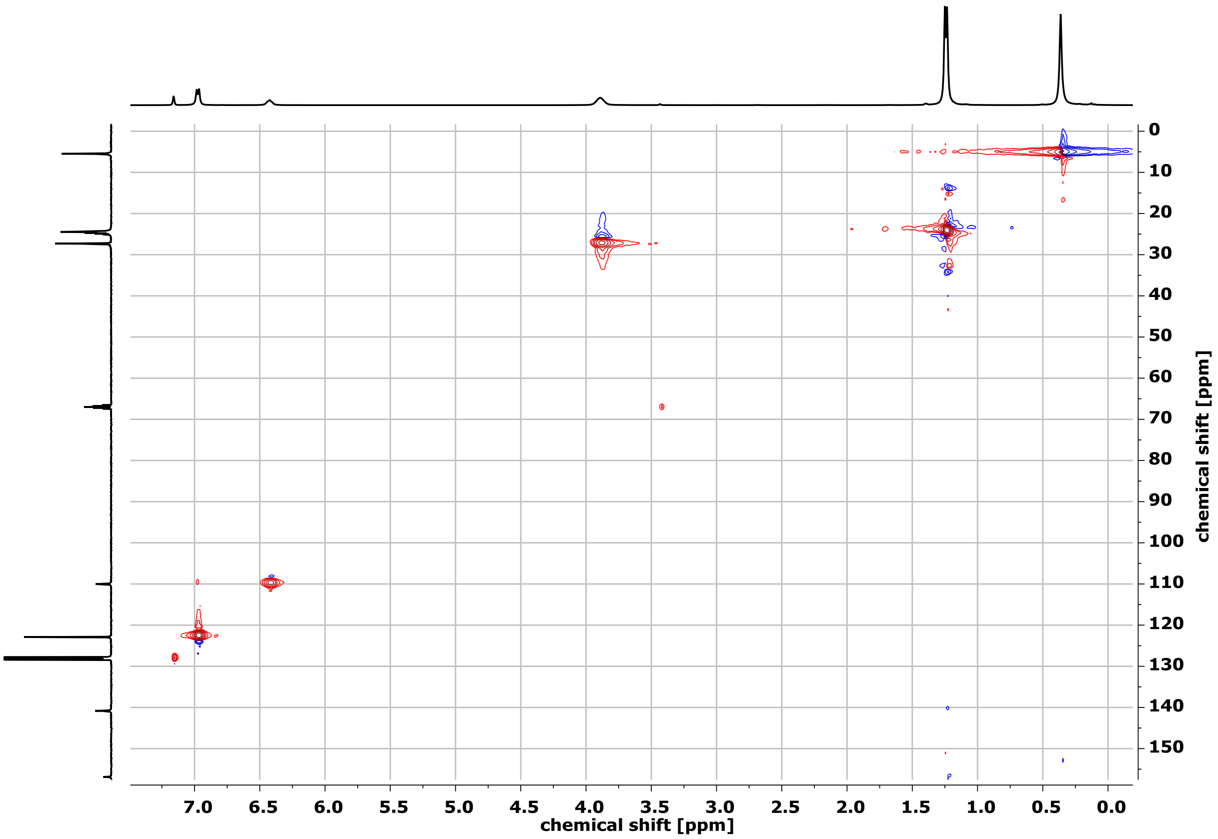

Figure S27. $^{1}H^{13}C$-HSQC NMR spectrum of [{Rb(N$^{`Ar}$)}$_n$] in a mixture of benzene-d_6:THF-$d8$ (10:1).

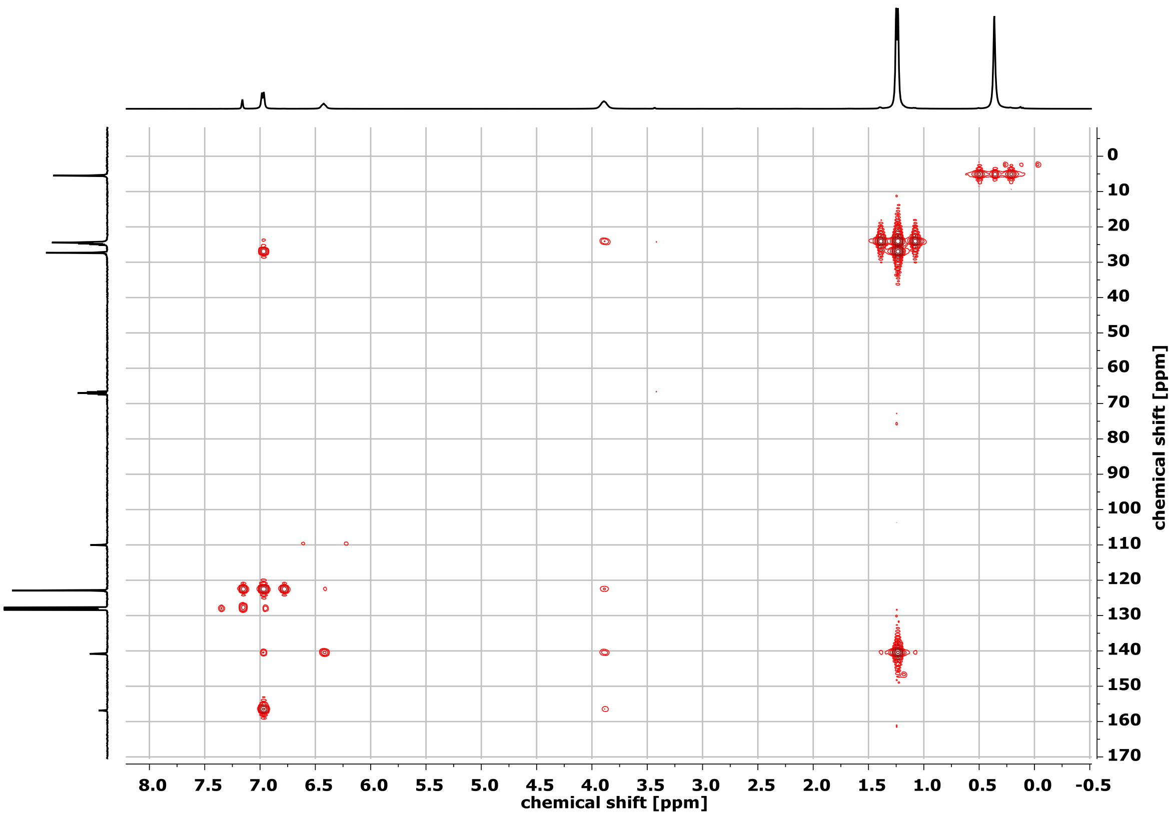

Figure S28. $^{1}H^{13}C$-HMBC NMR spectrum of [{Rb(N$^{`Ar}$)}$_n$] in a mixture of benzene-d_6:THF-$d8$ (10:1).

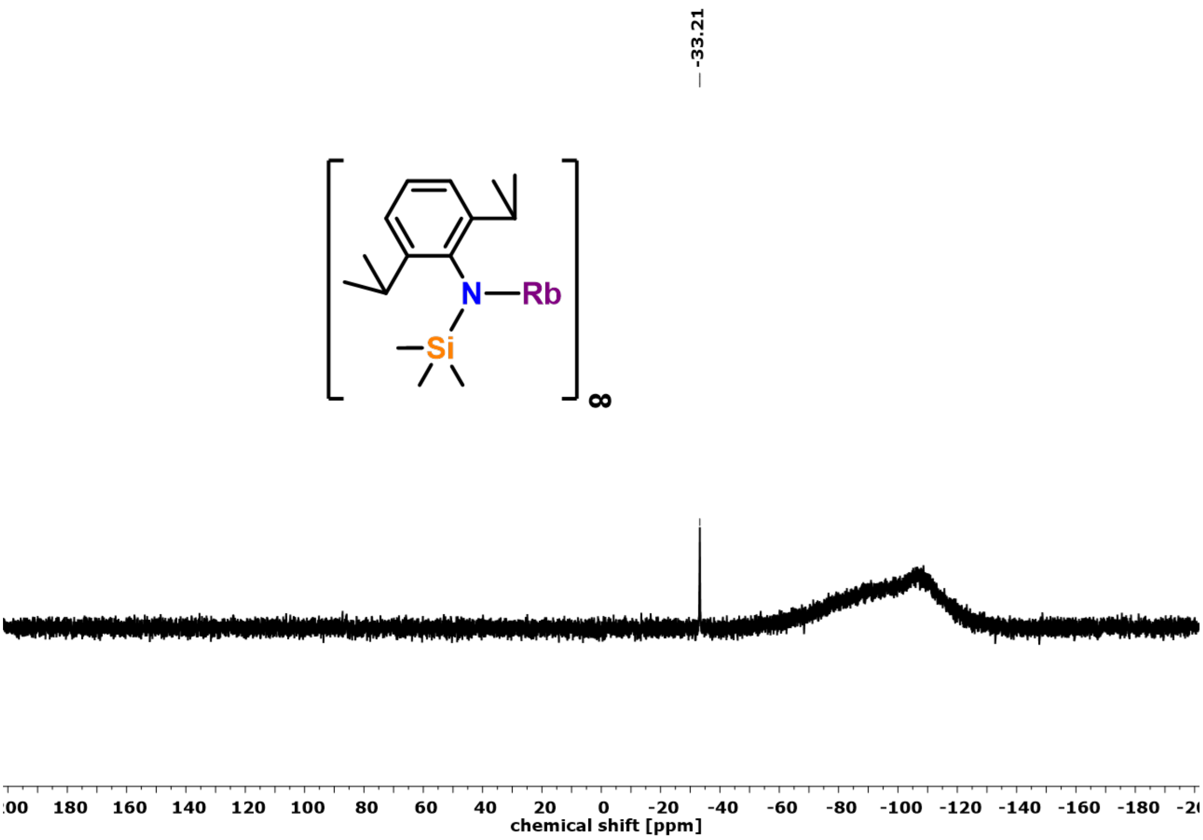

Figure S29. ^{29}Si NMR spectrum of [{Rb(N`^{Ar})}$_n$] in a mixture of benzene-d_6:THF-$d8$ (10:1).

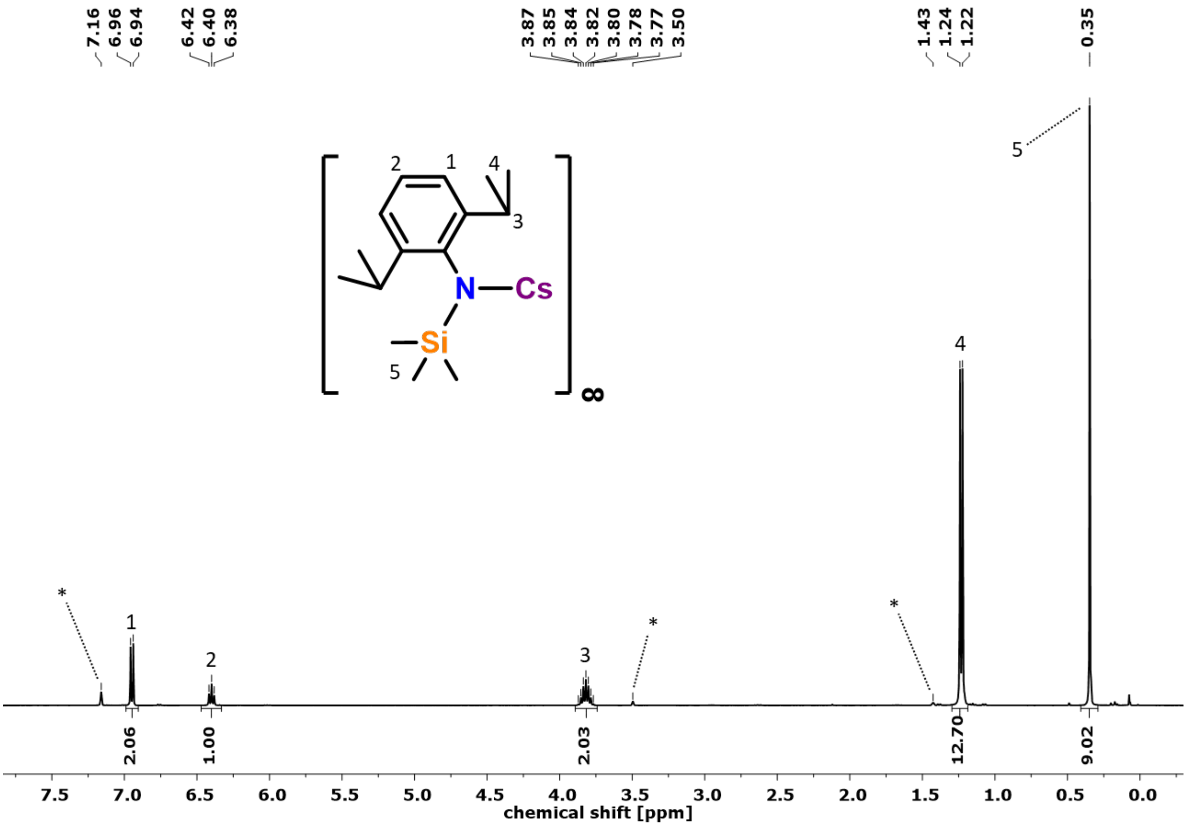

Figure S30. ^{1}H NMR spectrum of [{Cs(N`^{Ar})}$_n$] in a mixture of benzene-d_6:THF-$d8$ (10:1). Deuterated solvents marked with *.

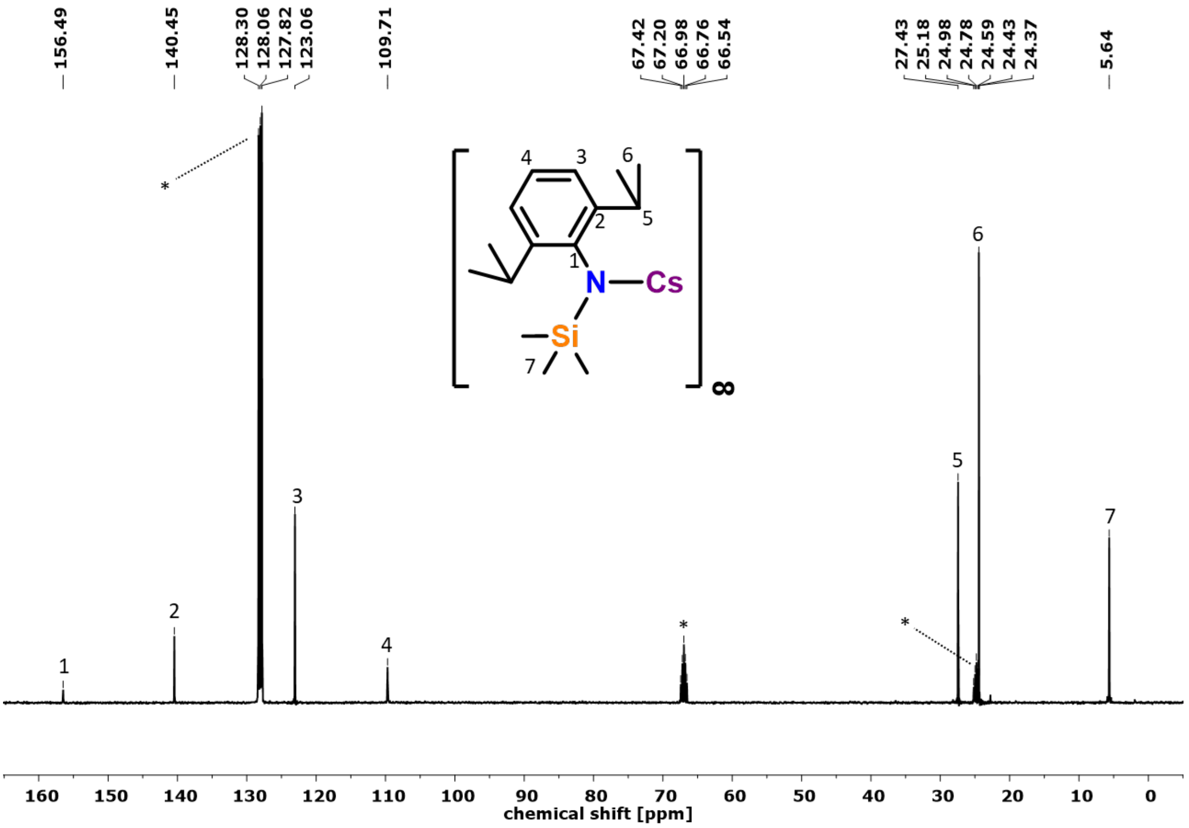

Figure S31. ^{13}C NMR spectrum of [{Cs(N$^{`Ar}$)}$_n$] in a mixture of benzene-d_6:THF-*d8* (10:1). Deuterated solvents marked with *.

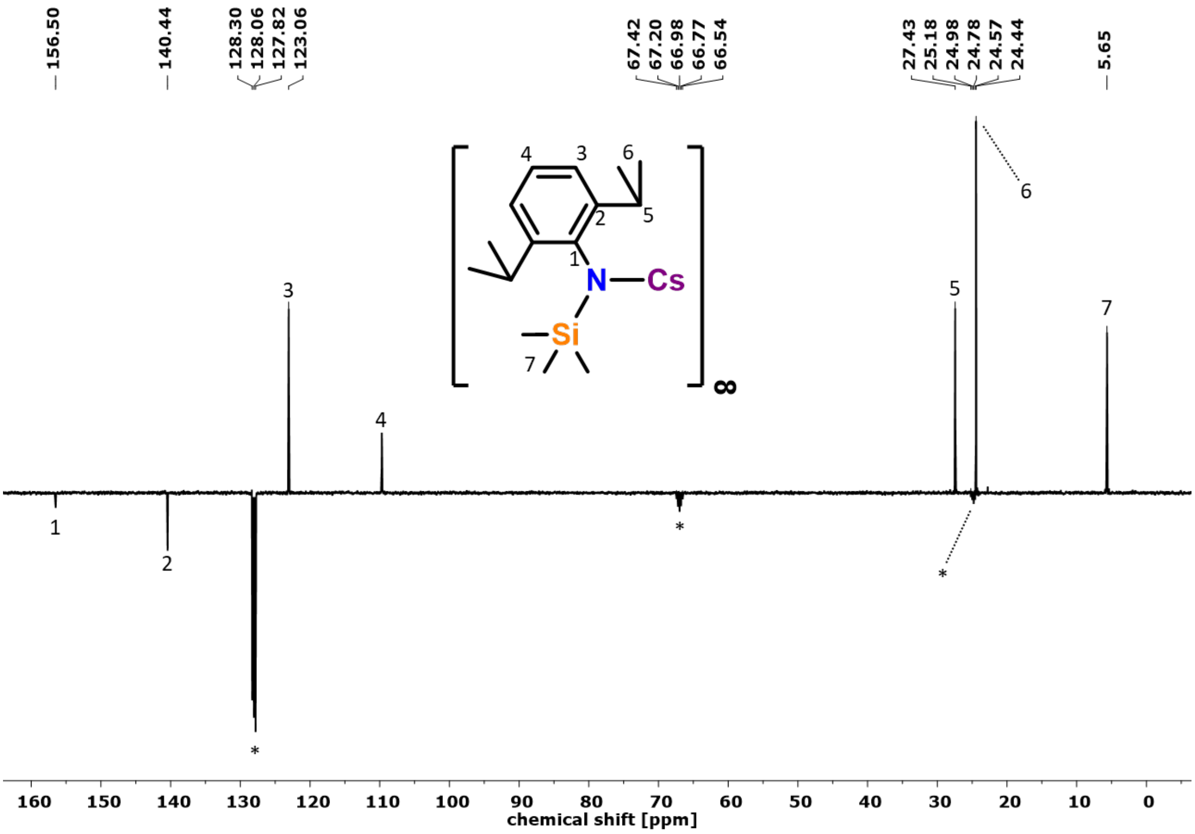

Figure S32. ^{13}C-DEPT-135 NMR spectrum of [{Cs(N$^{`Ar}$)}$_n$] in a mixture of benzene-d_6:THF-*d8* (10:1). Deuterated solvents marked with *.

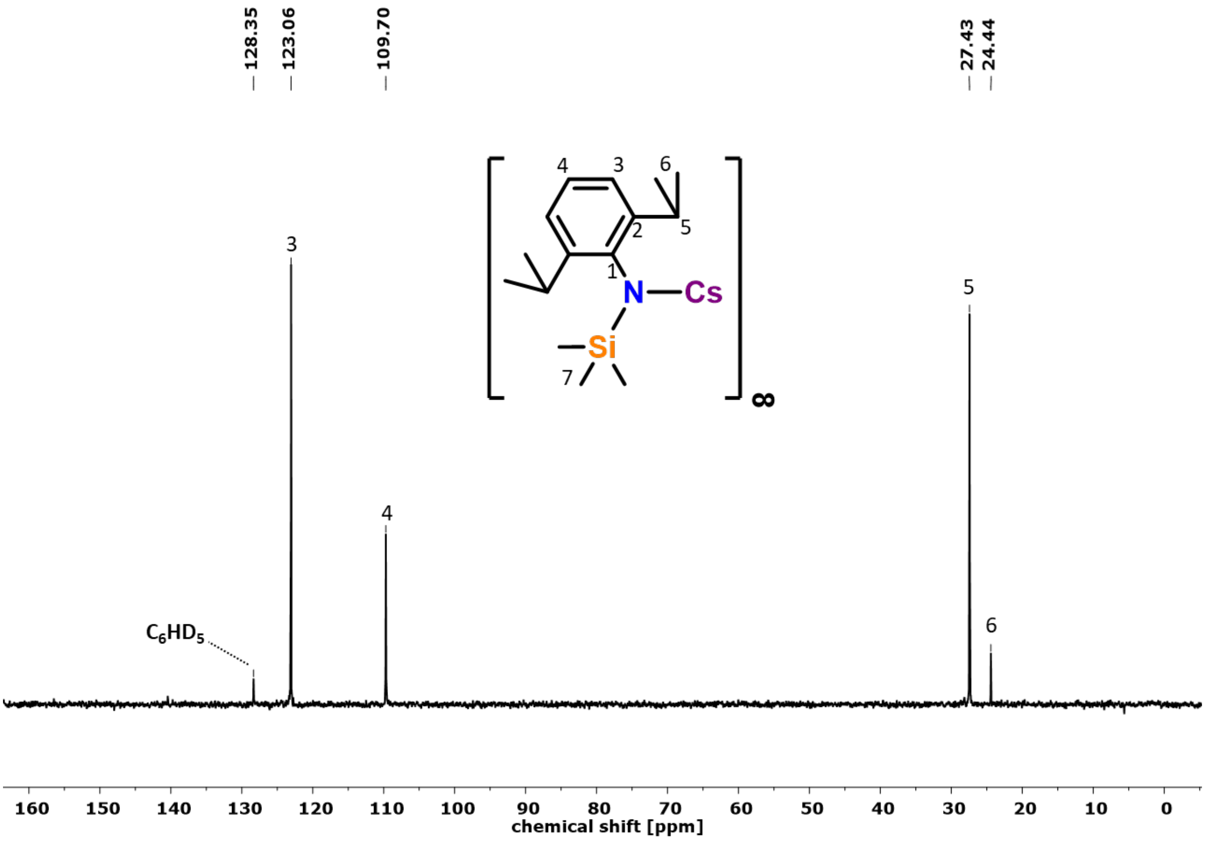

Figure S33. ^{13}C-DEPT-90 NMR spectrum of [{Cs(N$^{`Ar}$)}$_n$] in a mixture of benzene-d_6:THF-*d8* (10:1).

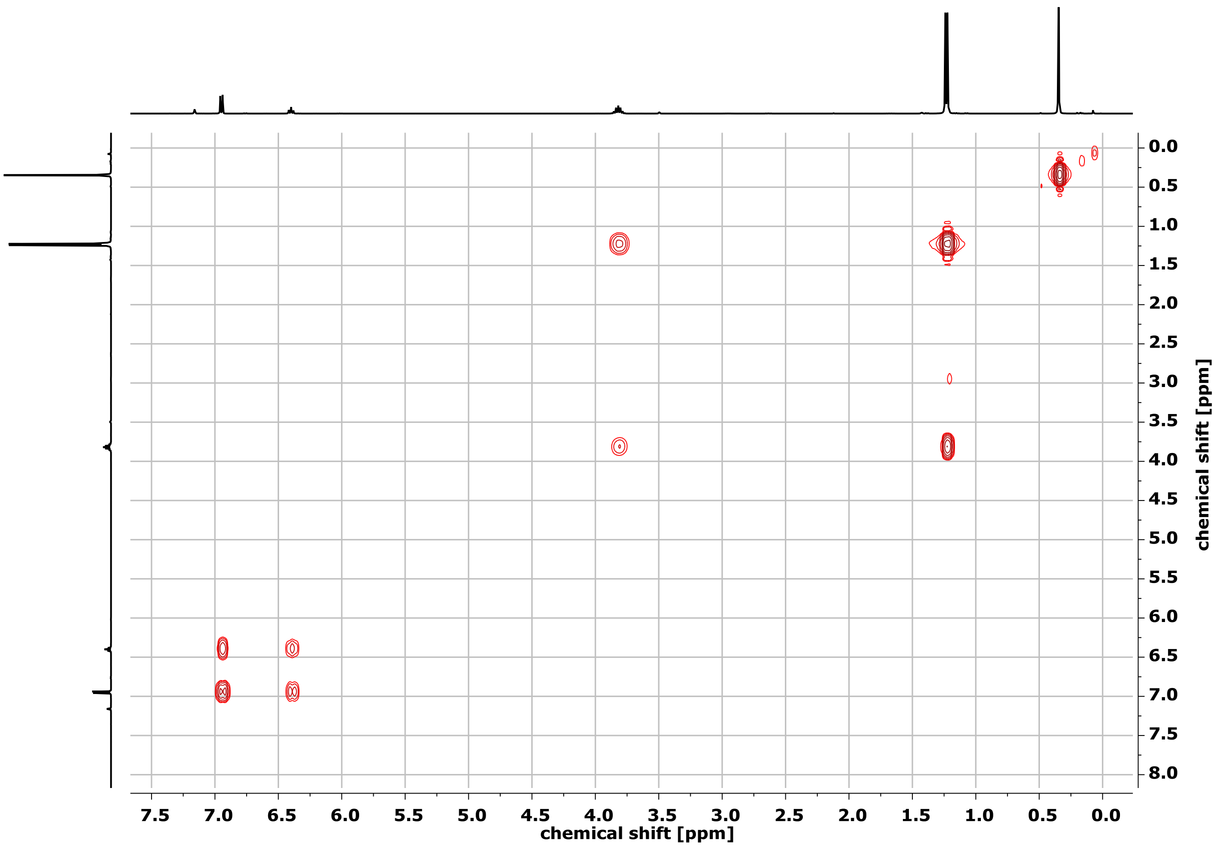

Figure S34. ^{1}H^{1}H-COSY NMR spectrum of [{Cs(N$^{`Ar}$)}$_n$] in a mixture of benzene-d_6:THF-*d8* (10:1).

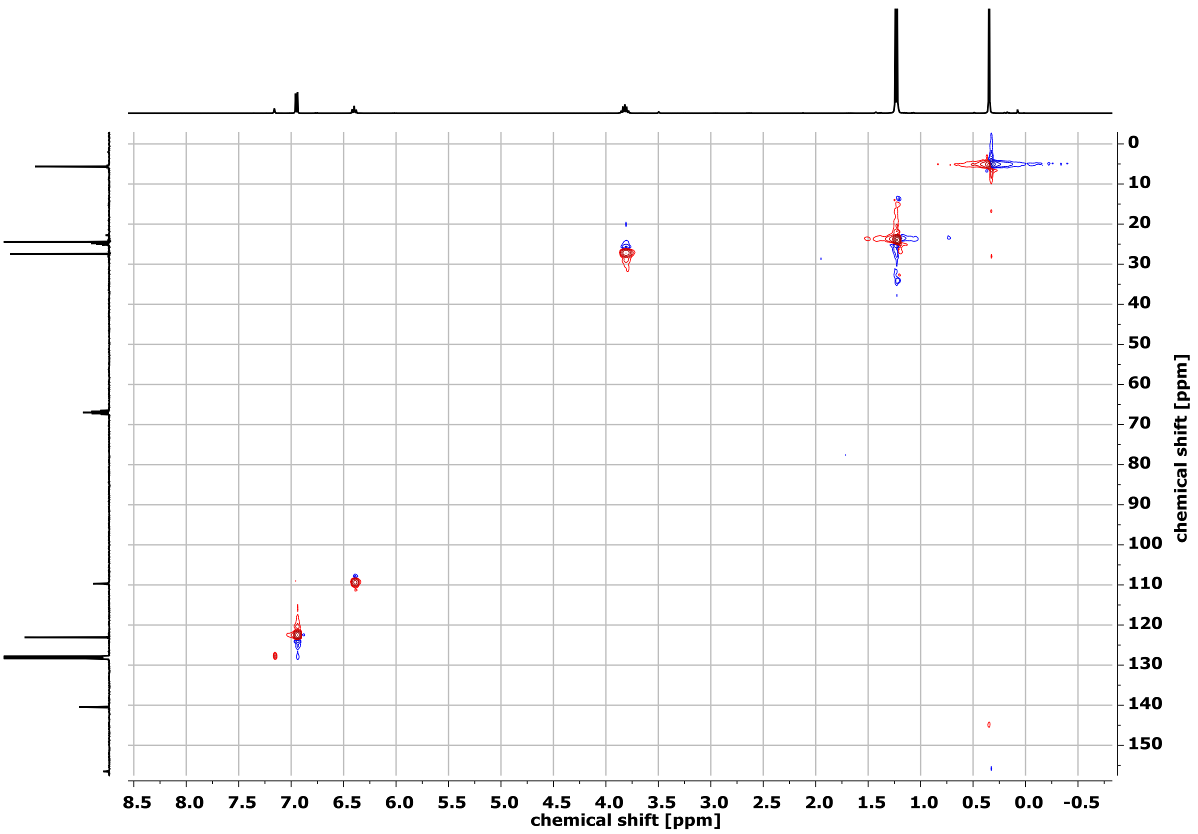

Figure S35. $^{1}H^{13}C$-HSQC NMR spectrum of [{Cs(N$^{`Ar}$)}$_n$] in a mixture of benzene-d_6:THF-*d8* (10:1).

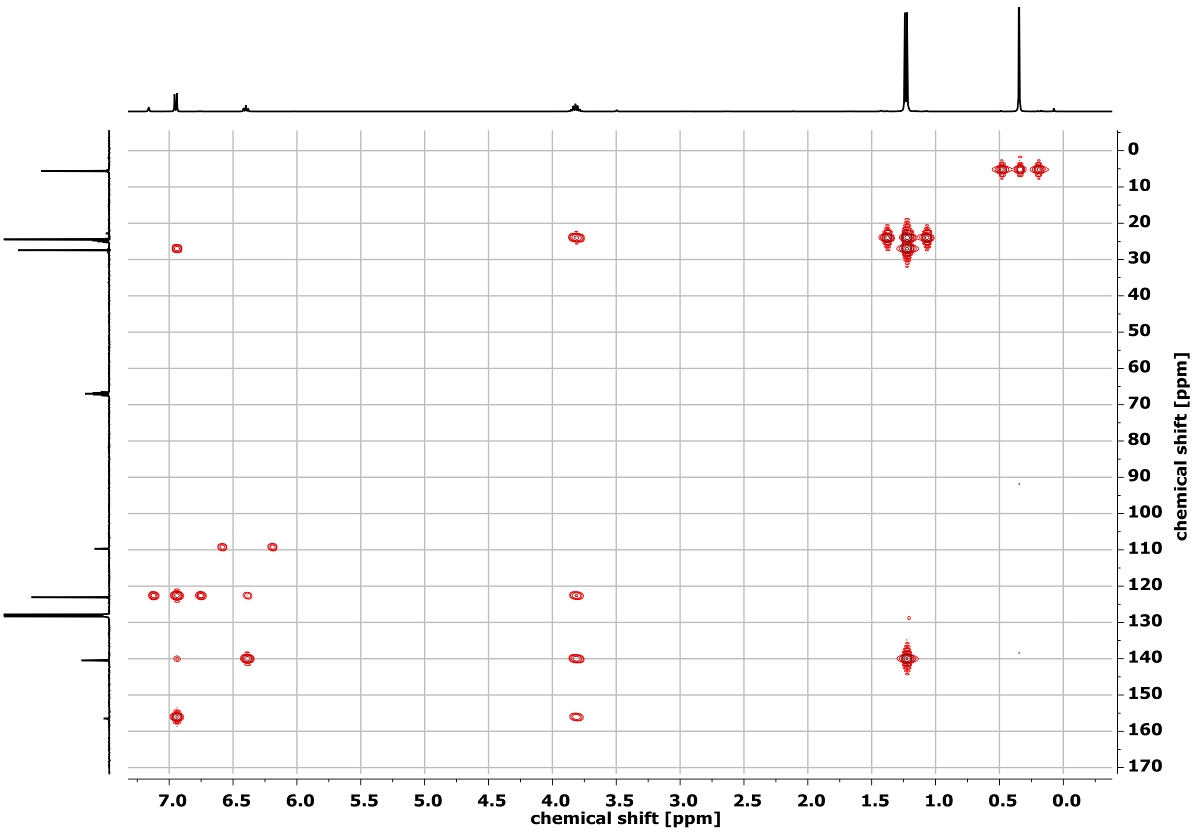

Figure S36. $^{1}H^{13}C$-HMBC NMR spectrum of [{Cs(N$^{`Ar}$)}$_n$] in a mixture of benzene-d_6:THF-*d8* (10:1).

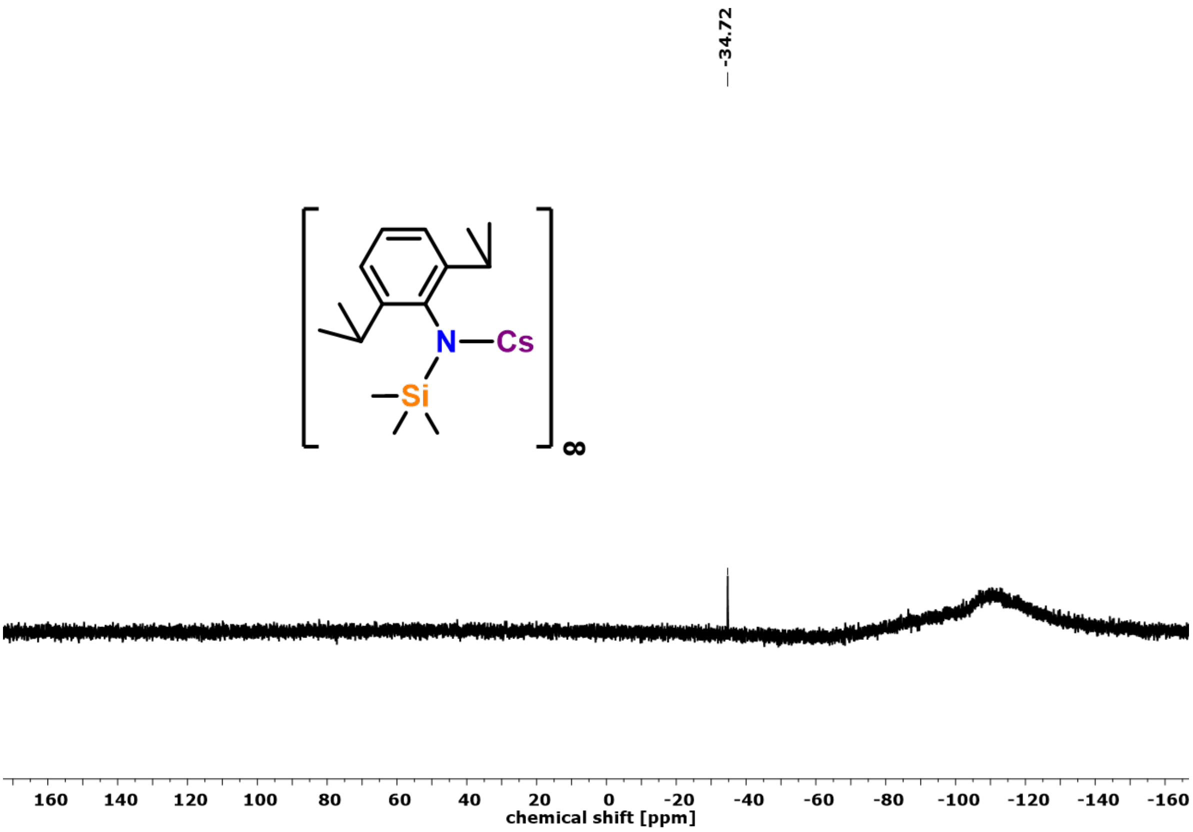

Figure S37. ^{29}Si NMR spectrum of [{Cs(N$^{`Ar}$)}$_n$] in a mixture of benzene-d_6:THF-$d8$ (10:1).

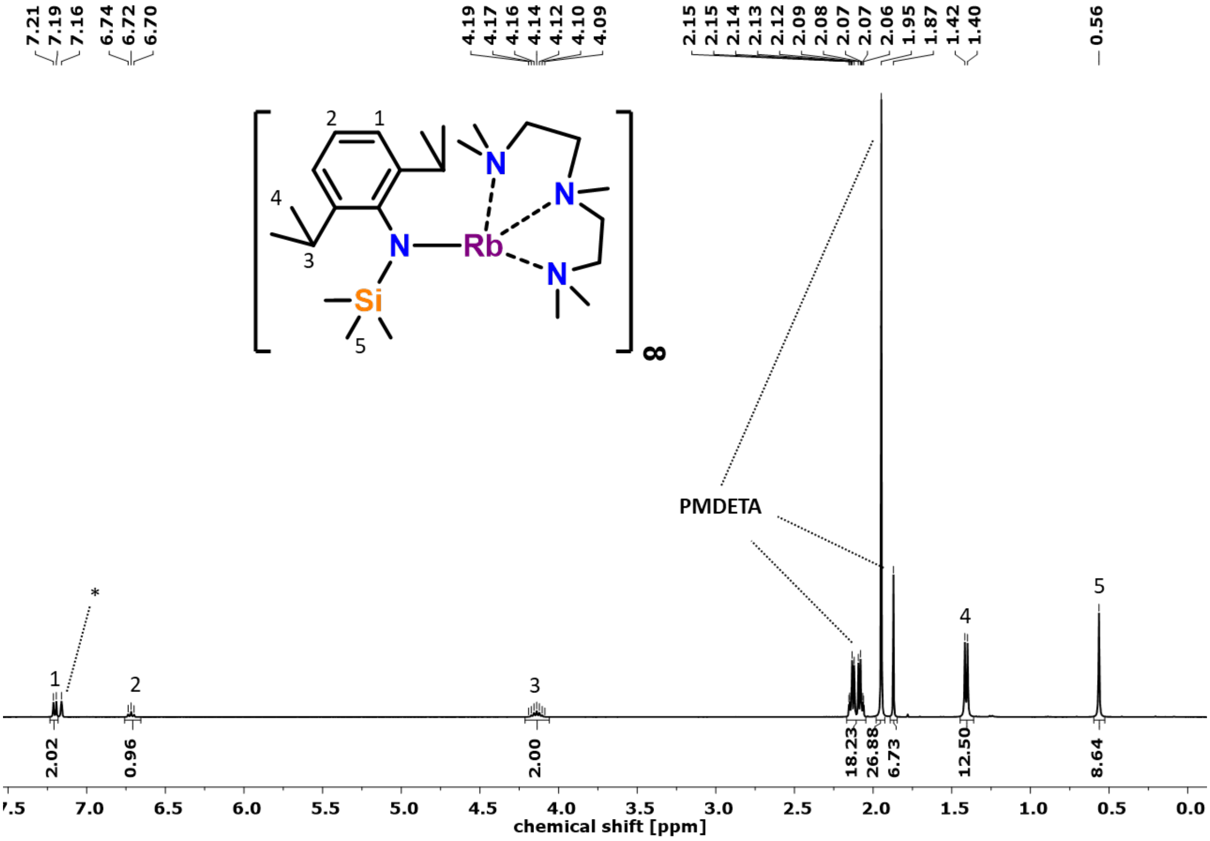

Figure S38. ^{1}H NMR spectrum of Rb{N(SiMe$_3$)Dipp}(PMDETA) in benzene-d_6 (*).

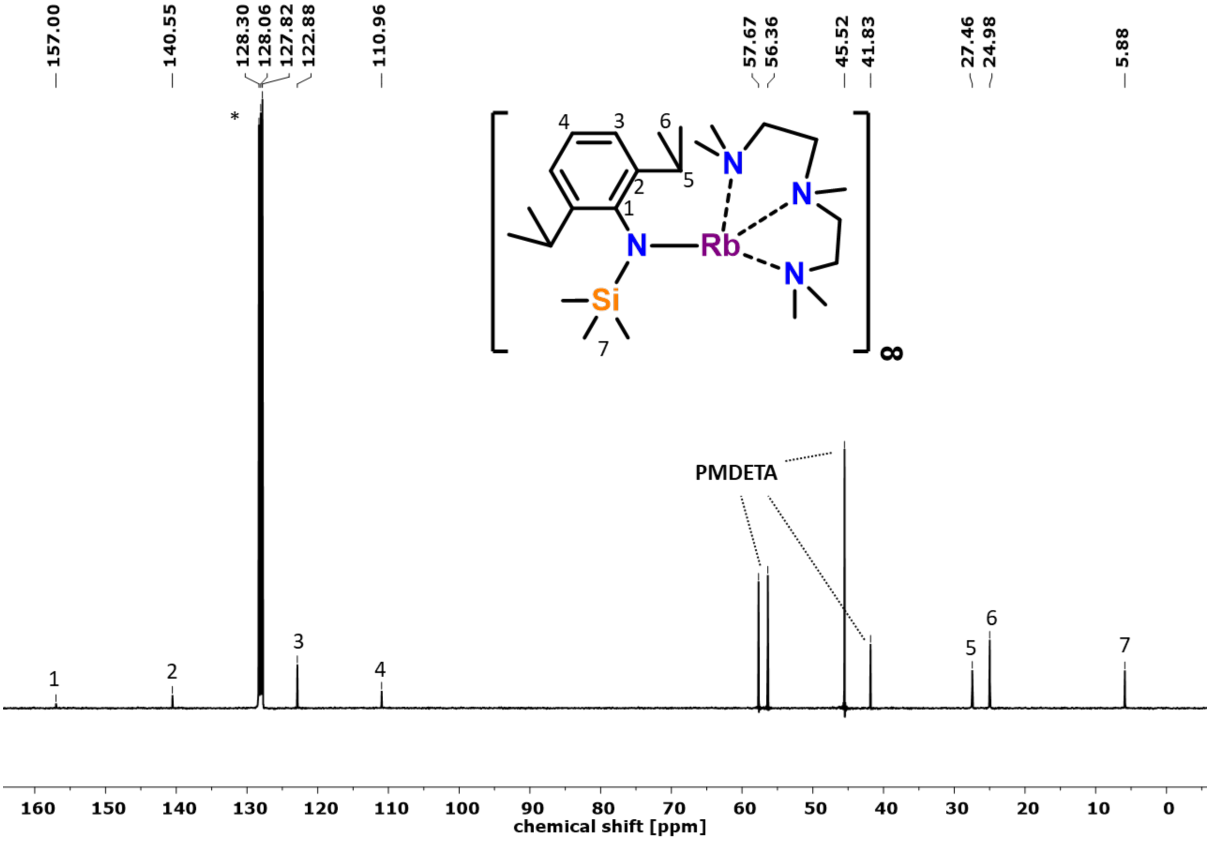

Figure S39. ^{13}C NMR spectrum of Rb{N(SiMe$_3$)Dipp}(PMDETA) in benzene-d_6 (*).

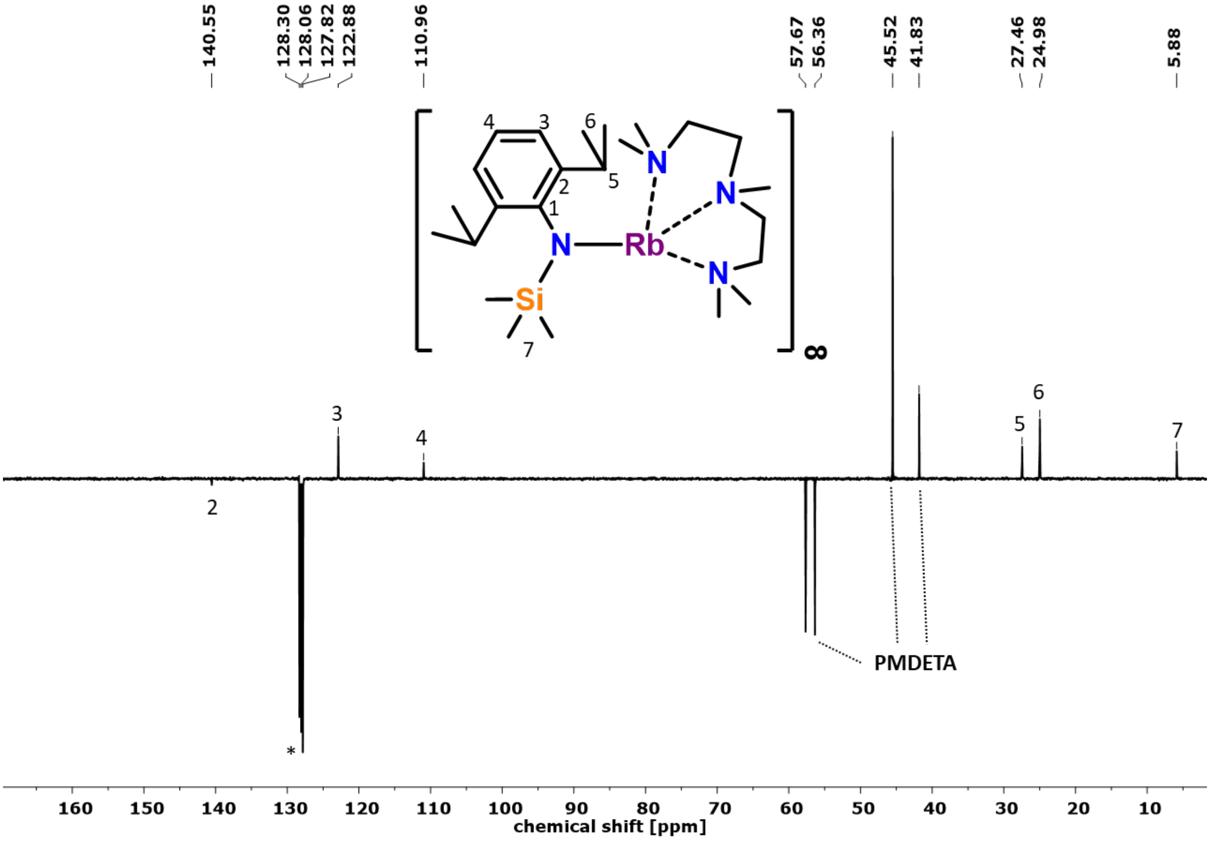

Figure S40. ^{13}C-DEPT-135 NMR spectrum of Rb{N(SiMe$_3$)Dipp}(PMDETA) in benzene-d_6 (*).

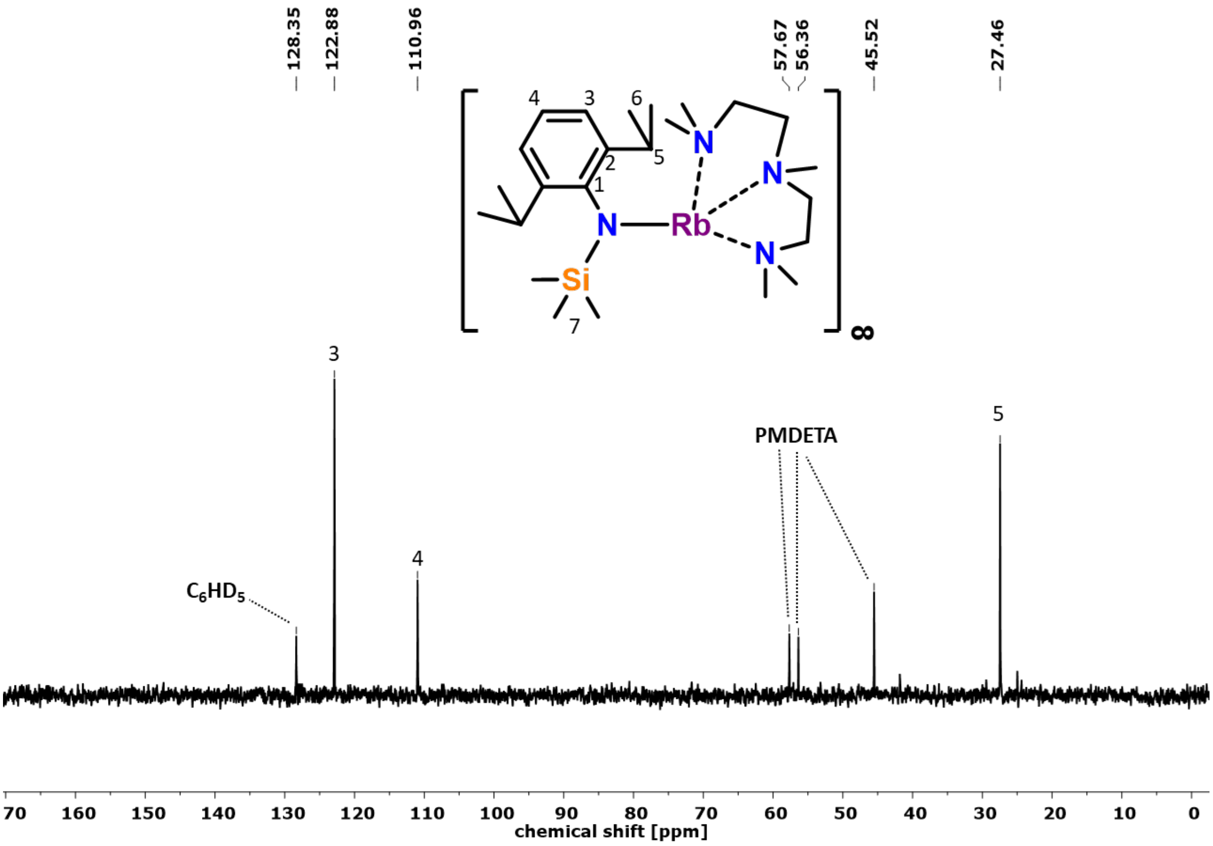

Figure S41. ^{13}C-DEPT-90 NMR spectrum of Rb{N(SiMe$_3$)Dipp}(PMDETA) in benzene-*d$_6$*.

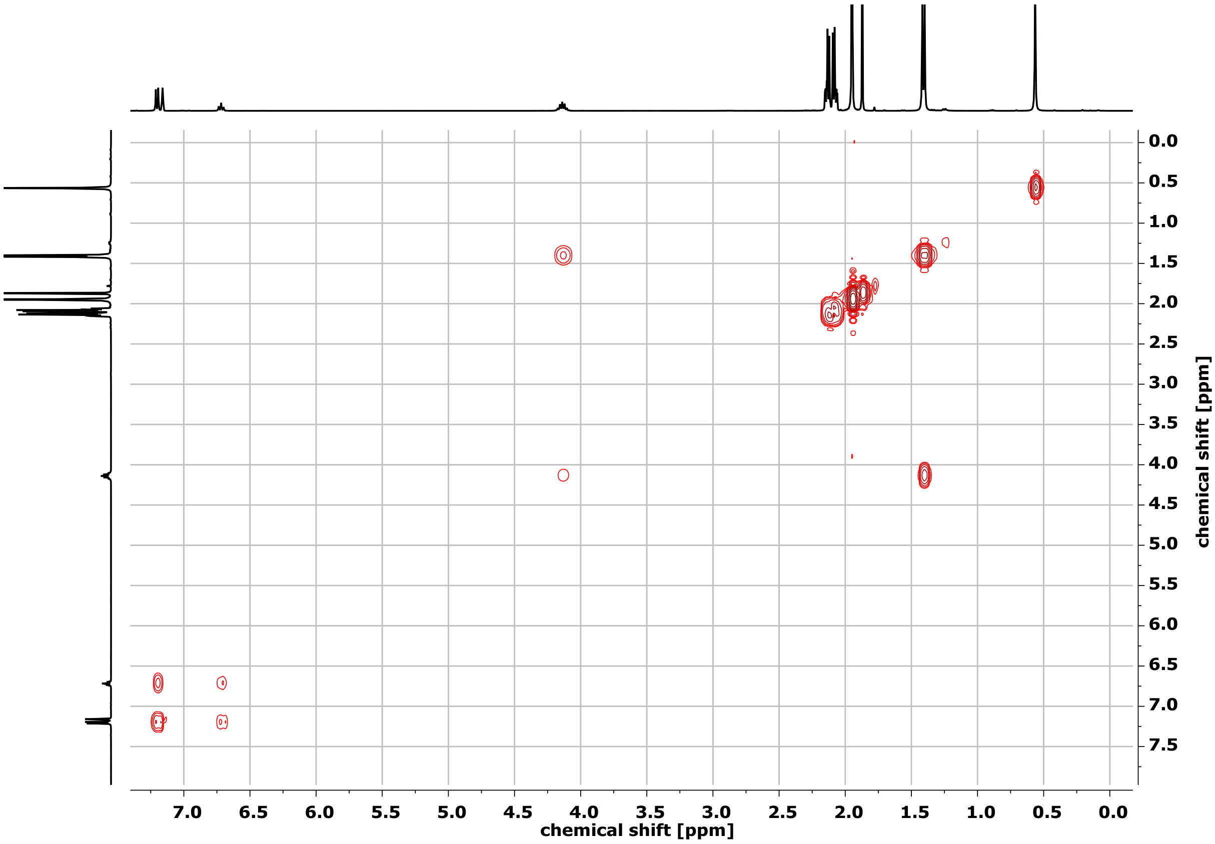

Figure S42. ^{1}H^{1}H-COSY NMR spectrum of Rb{N(SiMe$_3$)Dipp}(PMDETA) in benzene-*d$_6$*.

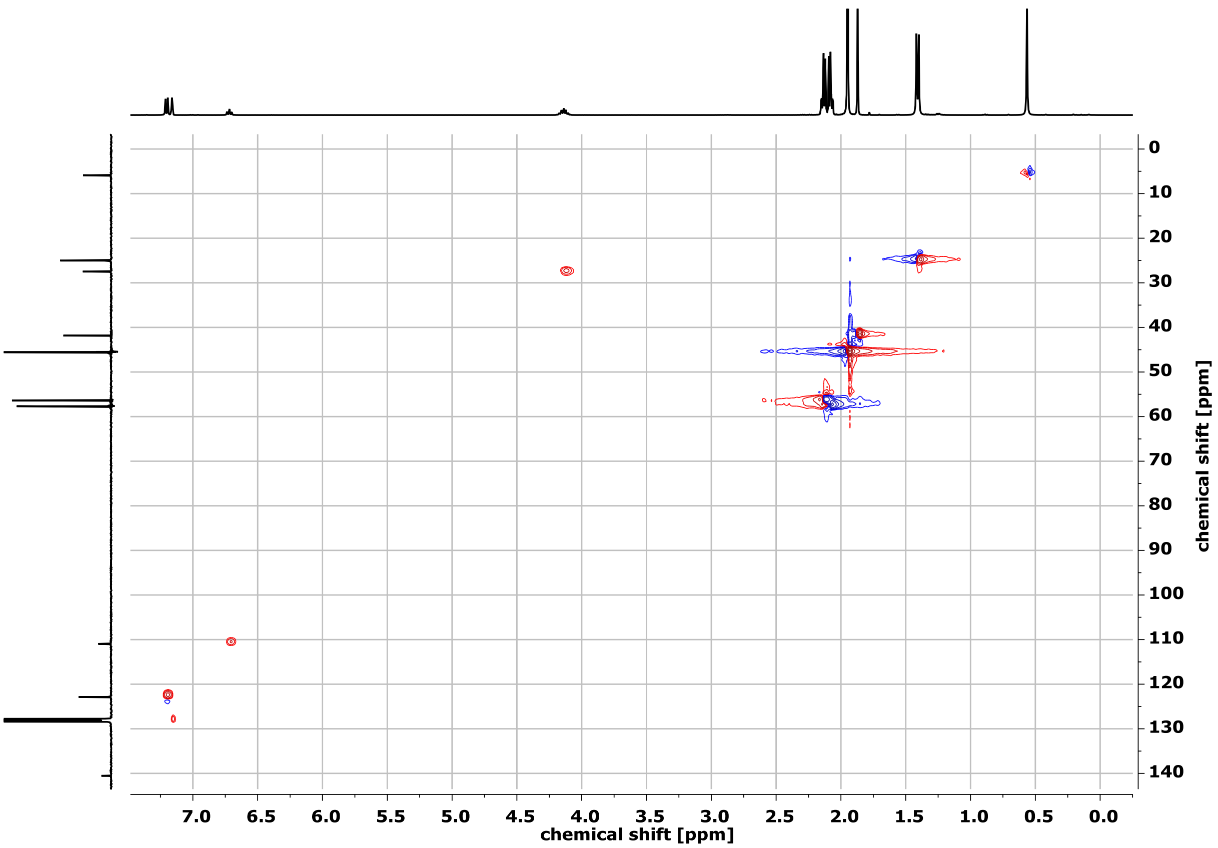

Figure S43. $^{1}H^{13}C$-HSQC NMR spectrum of Rb{N(SiMe$_3$)Dipp}(PMDETA) in benzene-d_6.

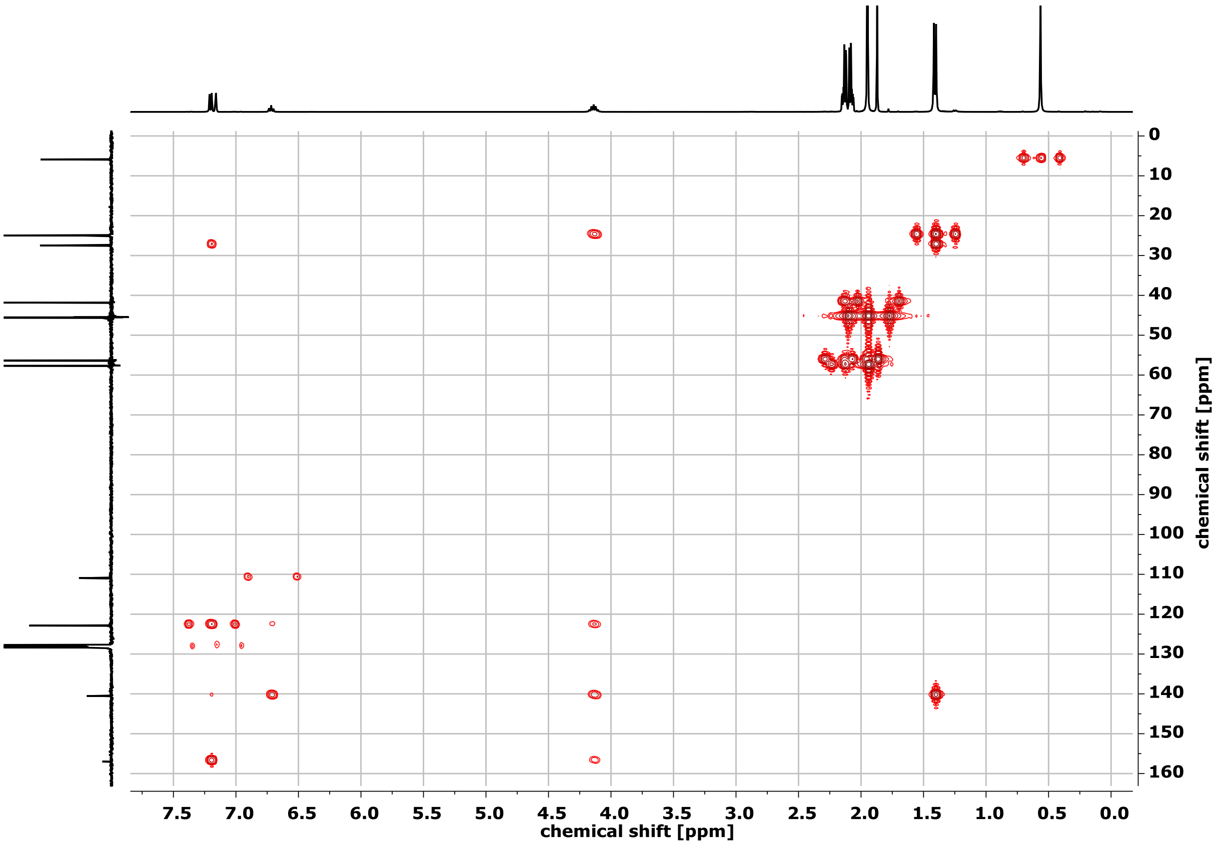

Figure S44. $^{1}H^{13}C$-HMBC NMR spectrum of Rb{N(SiMe$_3$)Dipp}(PMDETA) in benzene-d_6.

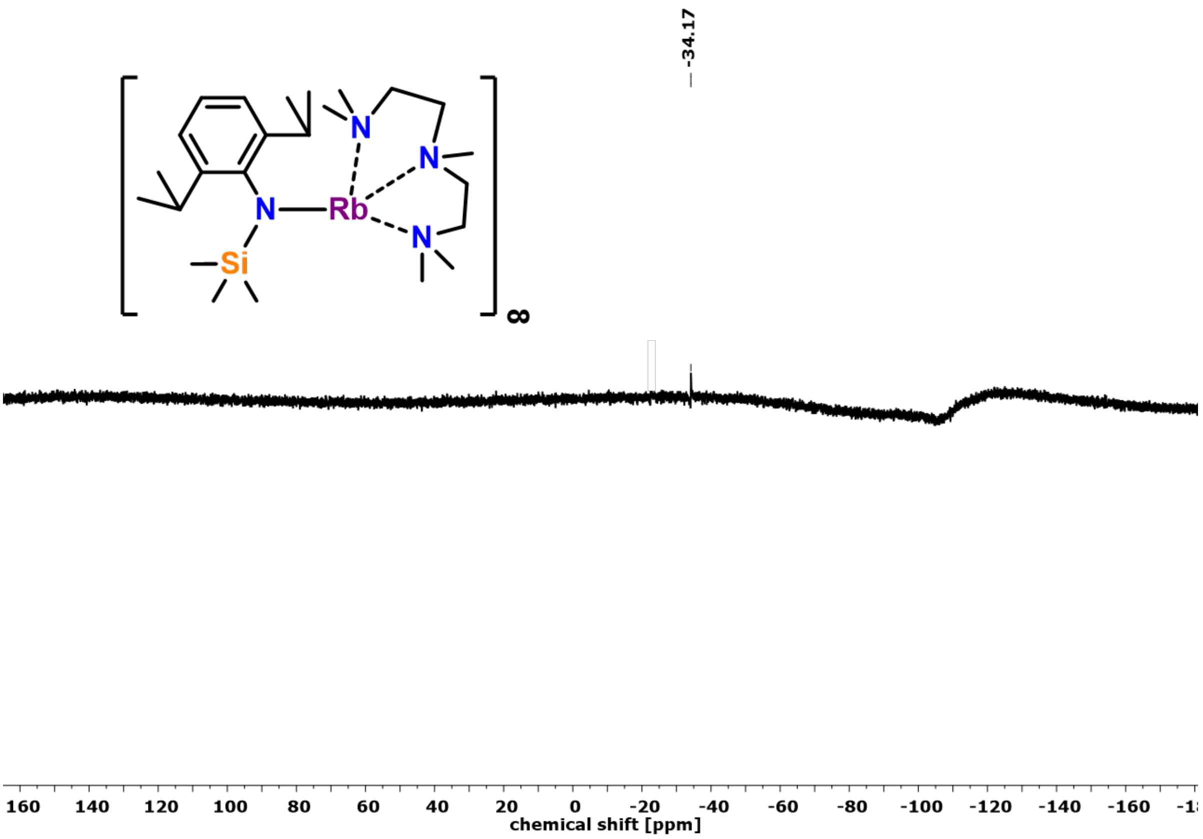

Figure S45. ^{29}Si NMR spectrum of Rb{N(SiMe$_3$)Dipp}(PMDETA) in benzene-d_6.

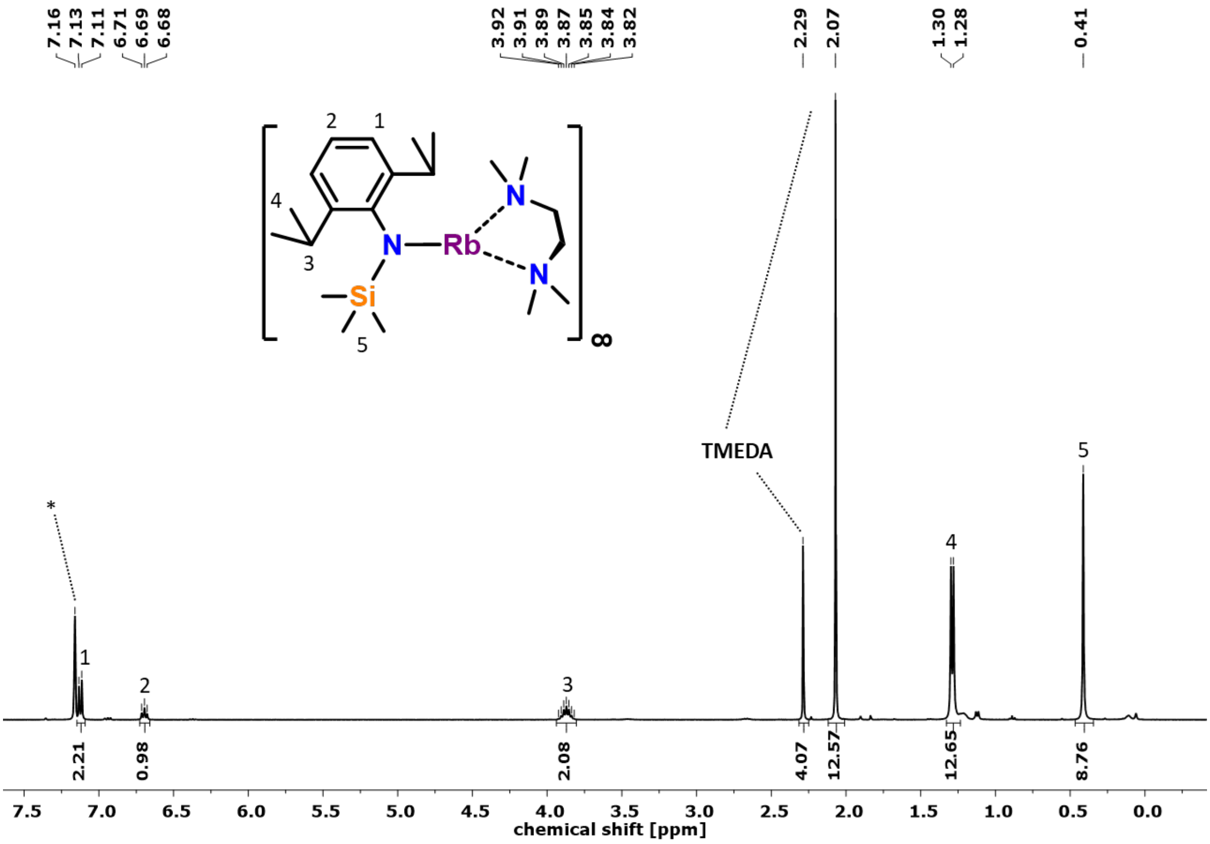

Figure S46. ^{1}H NMR spectrum of Rb{N(SiMe$_3$)Dipp}(TMEDA) in benzene-d_6 (*).

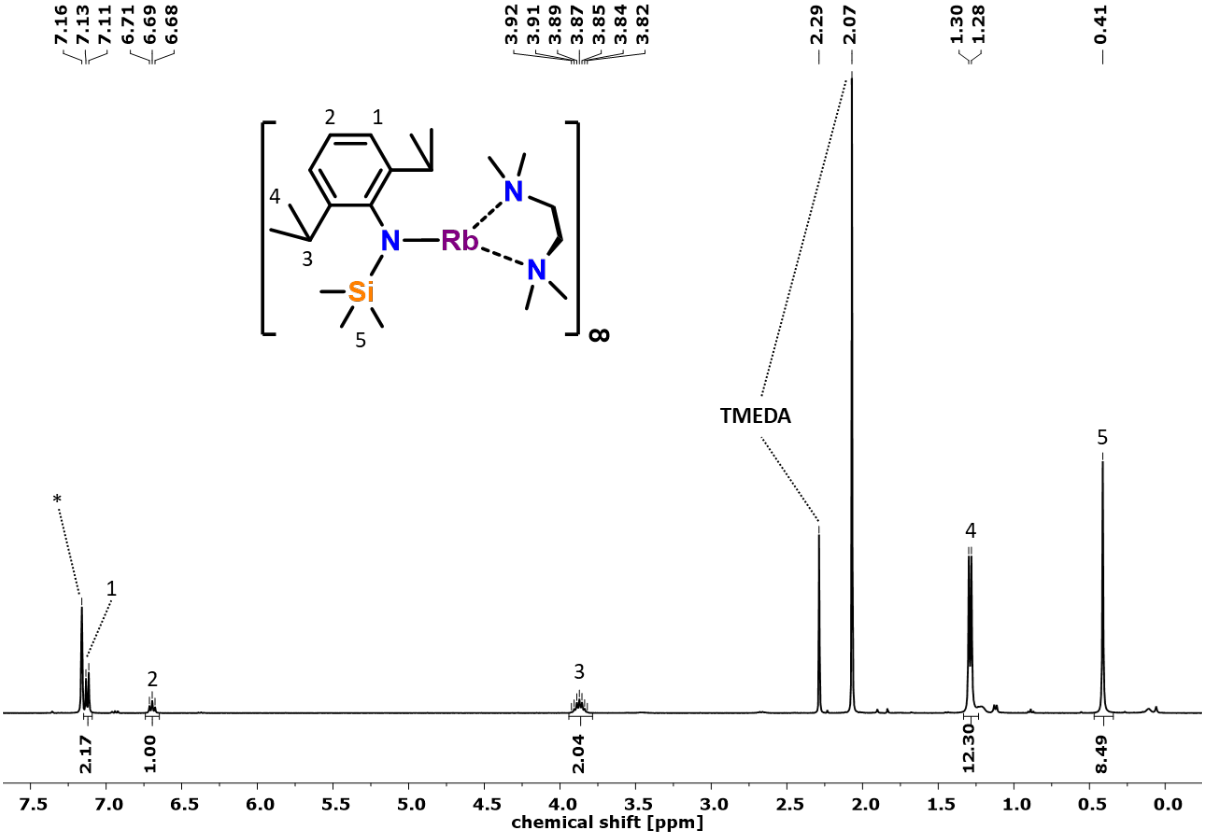

Figure S47. ^{13}C NMR spectrum of Rb{N(SiMe$_3$)Dipp}(TMEDA) in benzene-*d$_6$* (*).

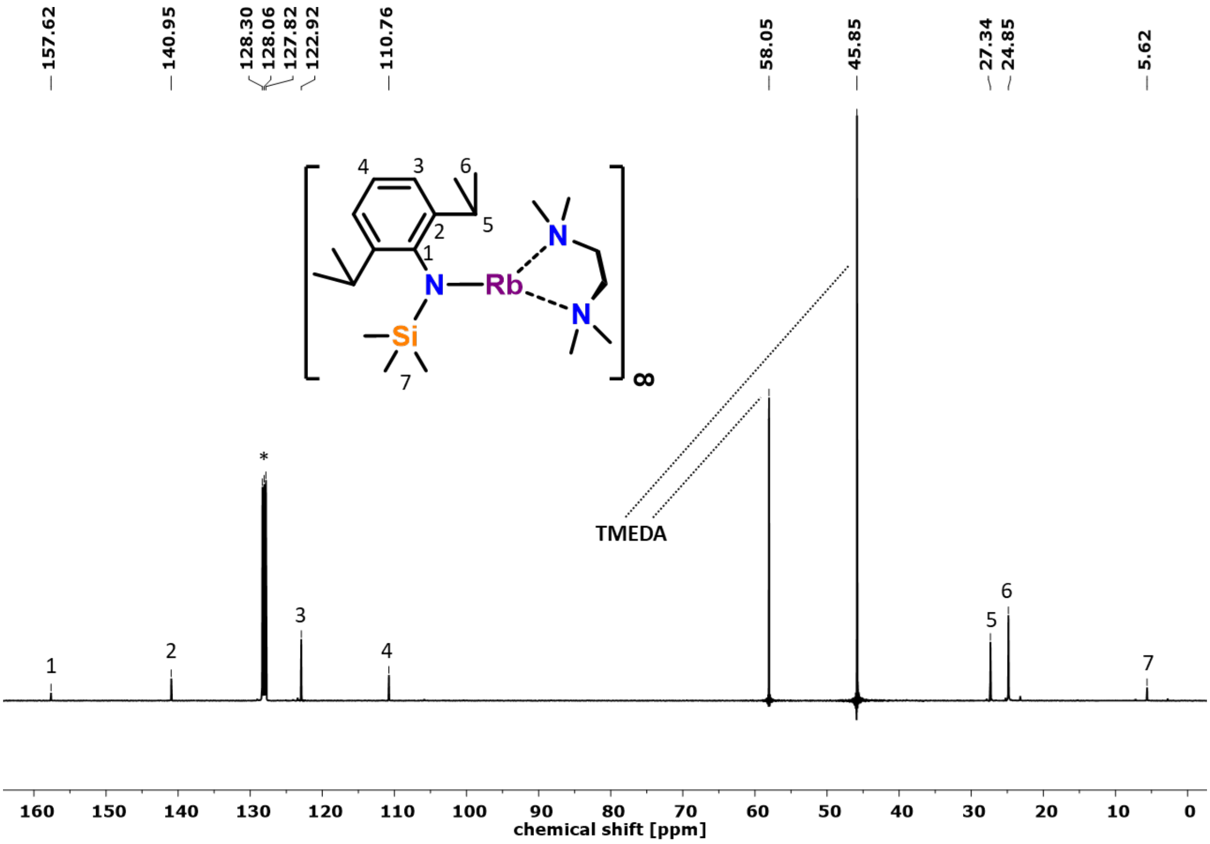

Figure S48. ^{13}C-DEPT-135 NMR spectrum of Rb{N(SiMe$_3$)Dipp}(TMEDA) in benzene-*d$_6$* (*).

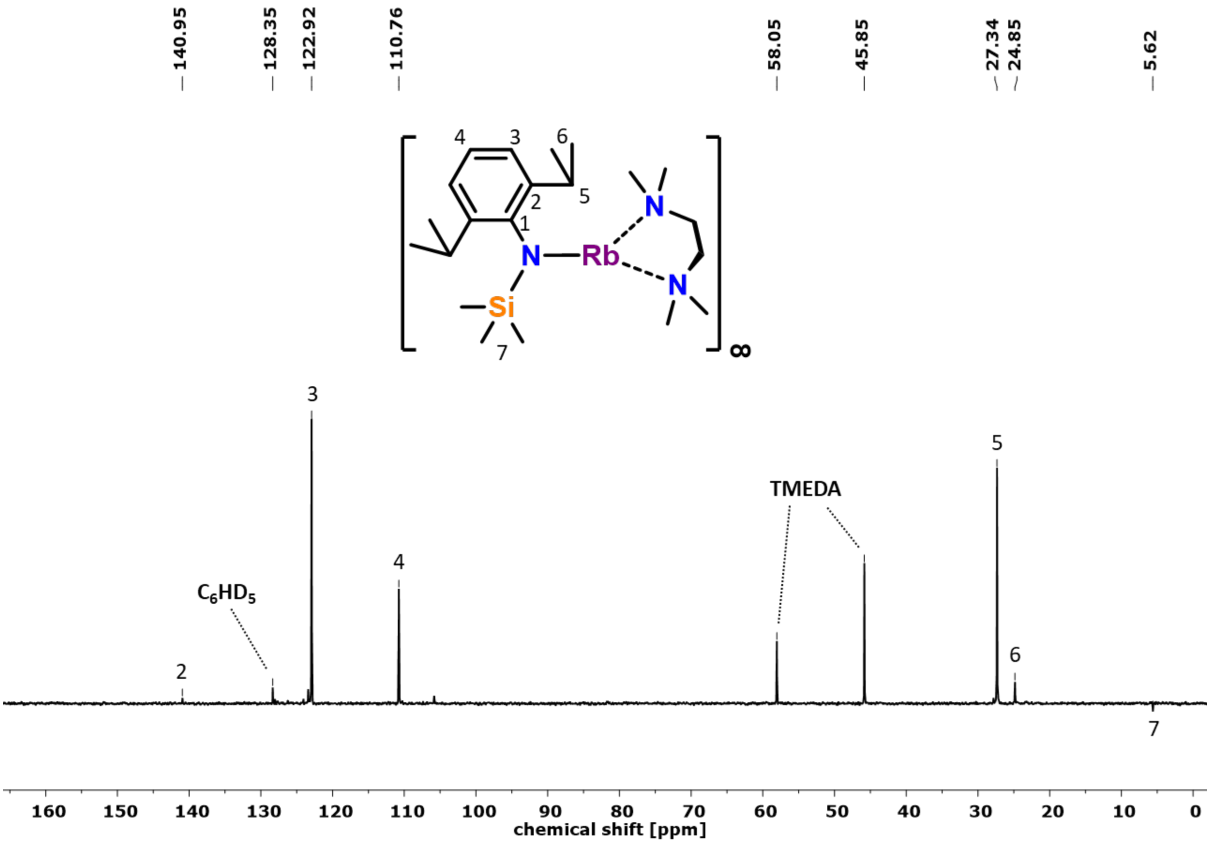

Figure 49. ^{13}C-DEPT-90 NMR spectrum of Rb{N(SiMe$_3$)Dipp}(TMEDA) in benzene-d_6.

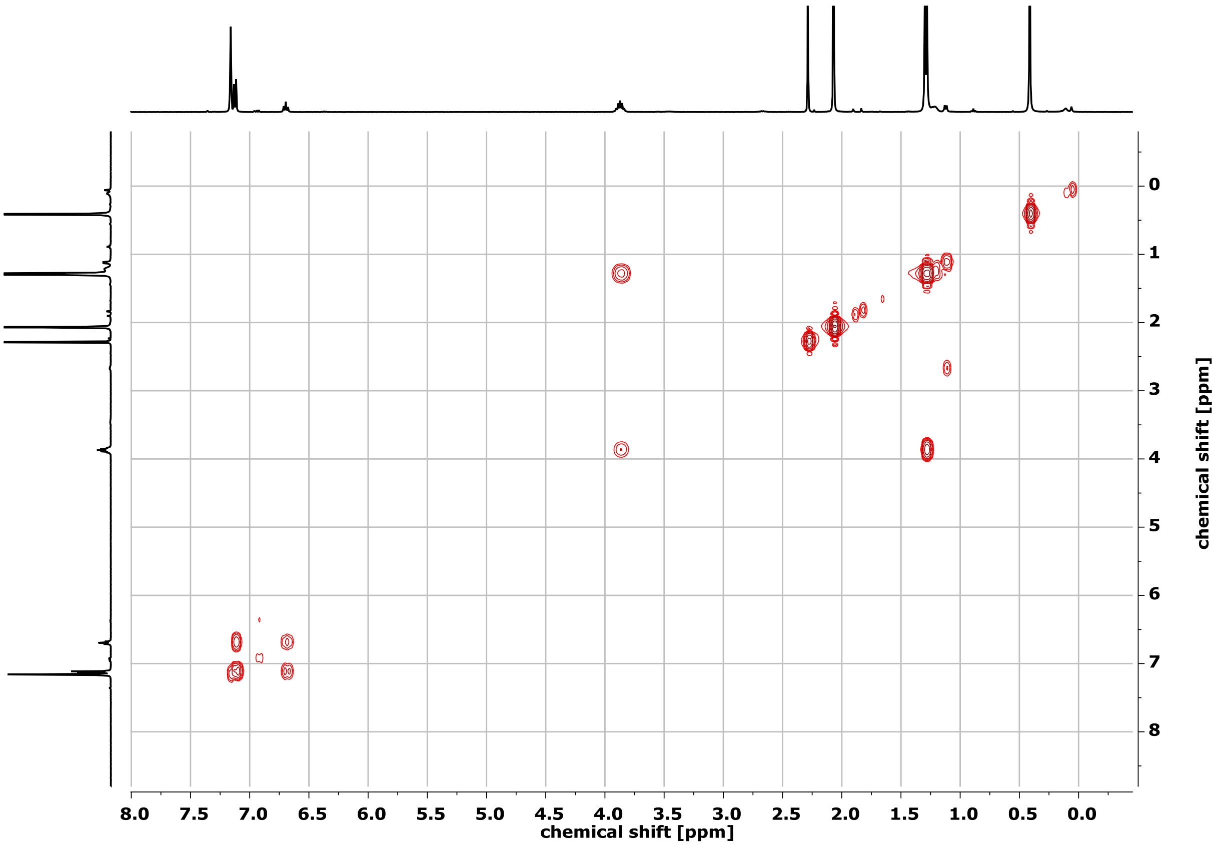

Figure S50. ^{1}H^{1}H-COSY NMR spectrum of Rb{N(SiMe$_3$)Dipp}(TMEDA) in benzene-d_6.

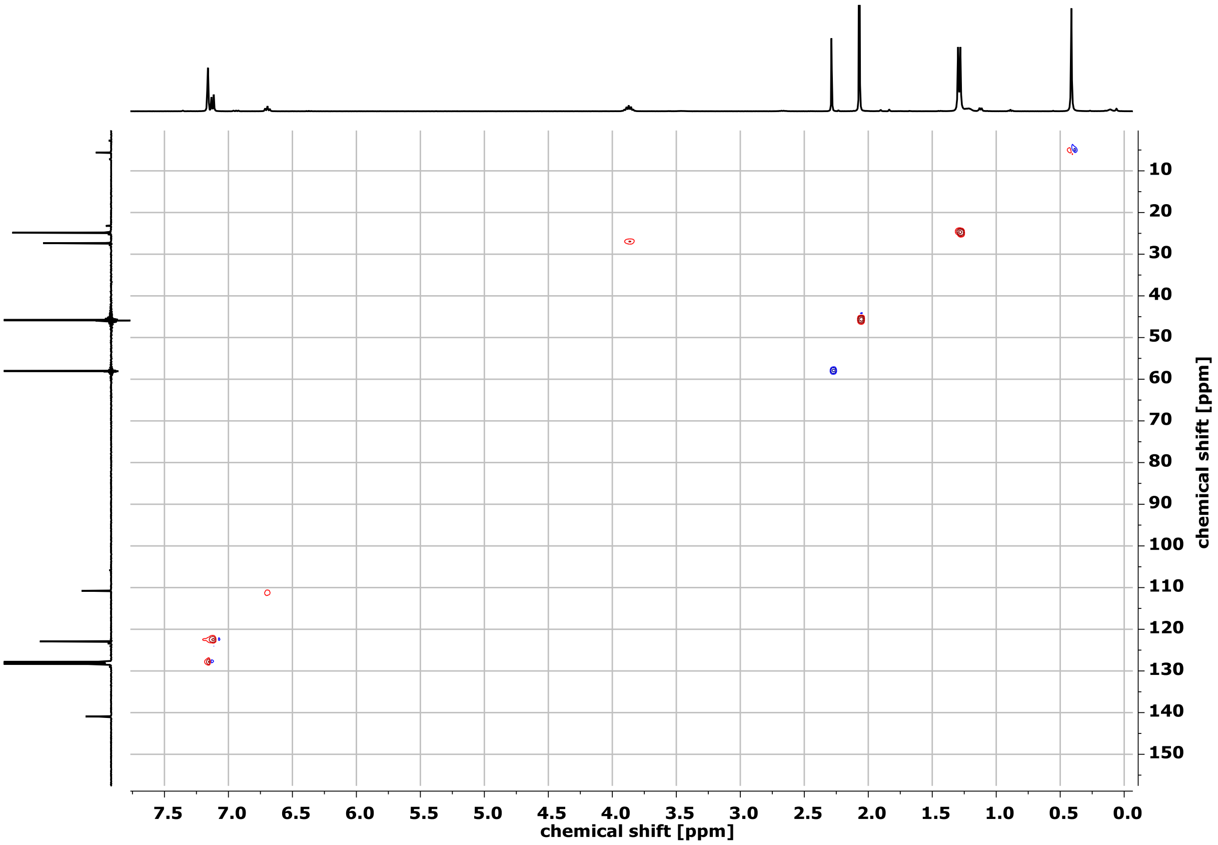

Figure S51. $^{1}H^{13}C$-HSQC NMR spectrum of Rb{N(SiMe$_3$)Dipp}(TMEDA) in benzene-d_6.

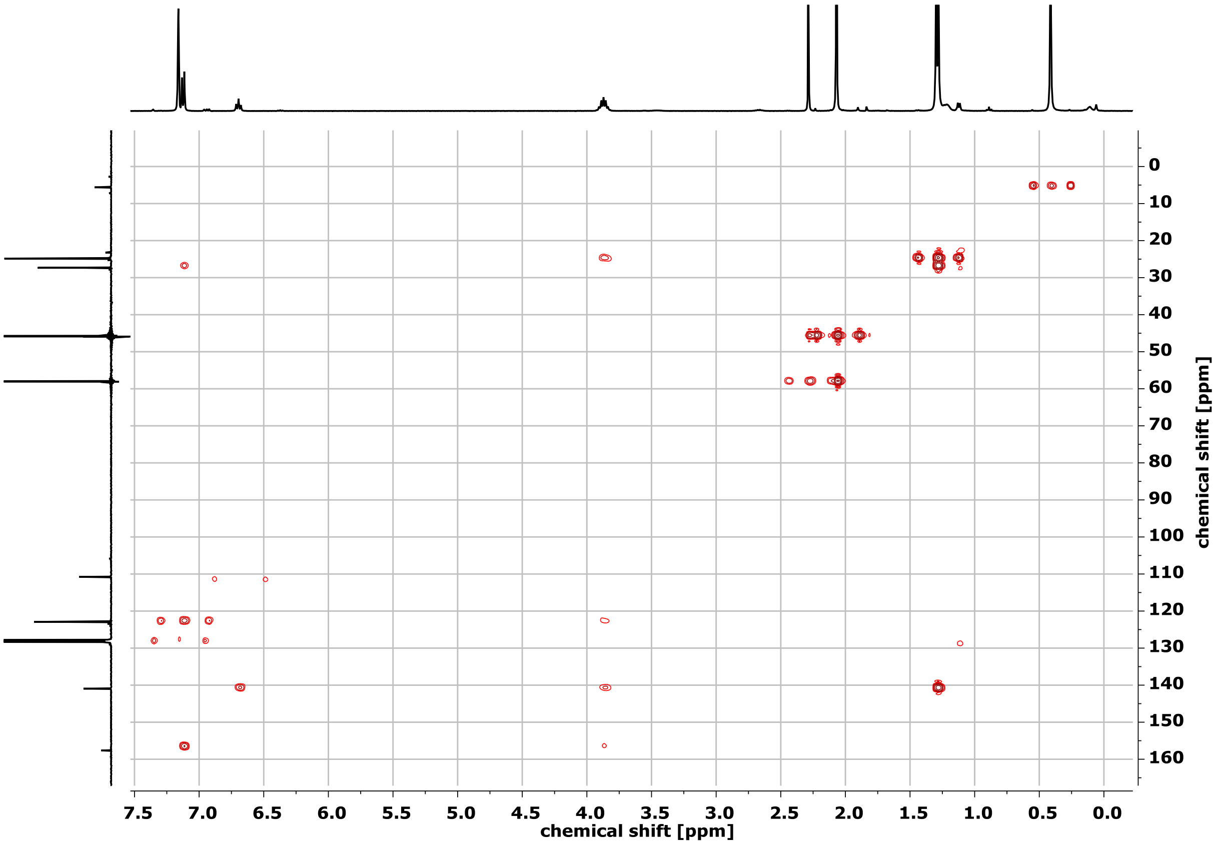

Figure S52. $^{1}H^{13}C$-HMBC NMR spectrum of Rb{N(SiMe$_3$)Dipp}(TMEDA) in benzene-d_6.

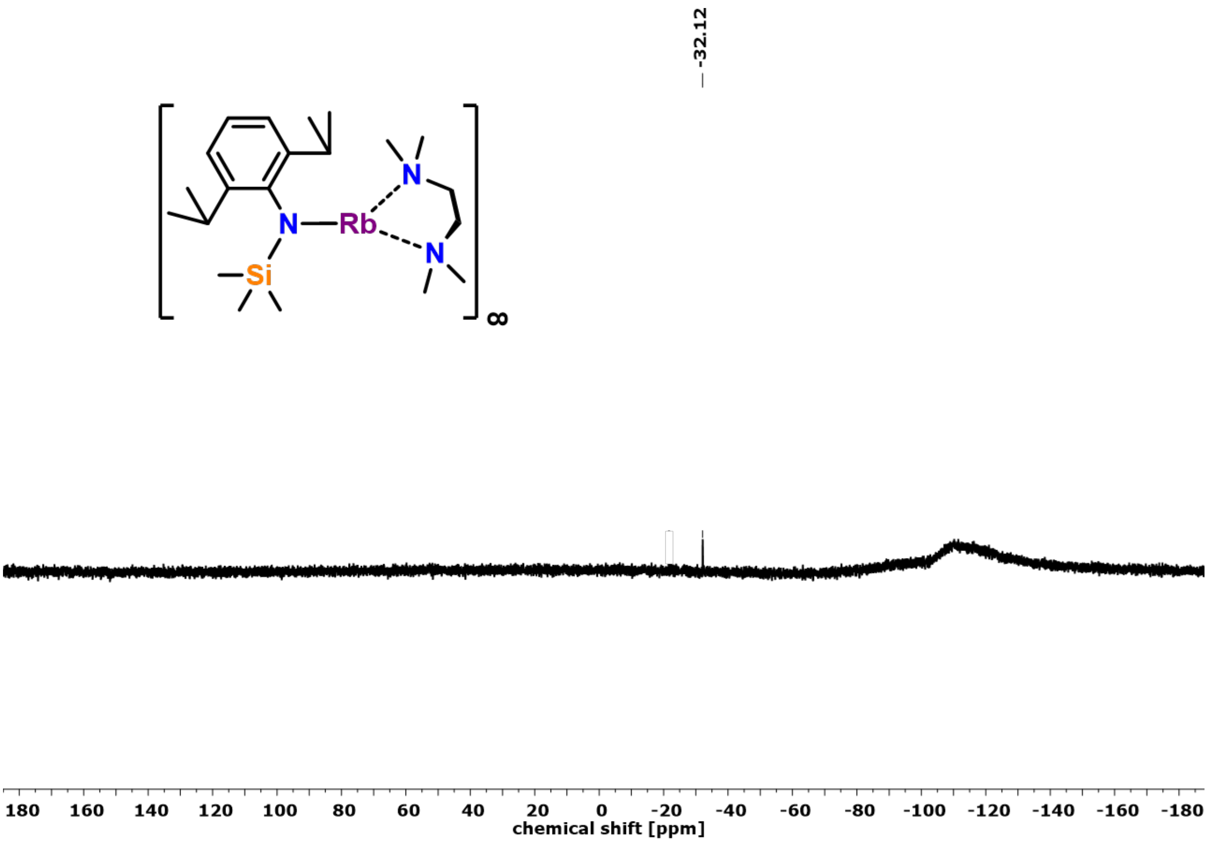

Figure S53. ^{29}Si NMR spectrum of Rb{N(SiMe$_3$)Dipp}(TMEDA) in benzene-d_6.

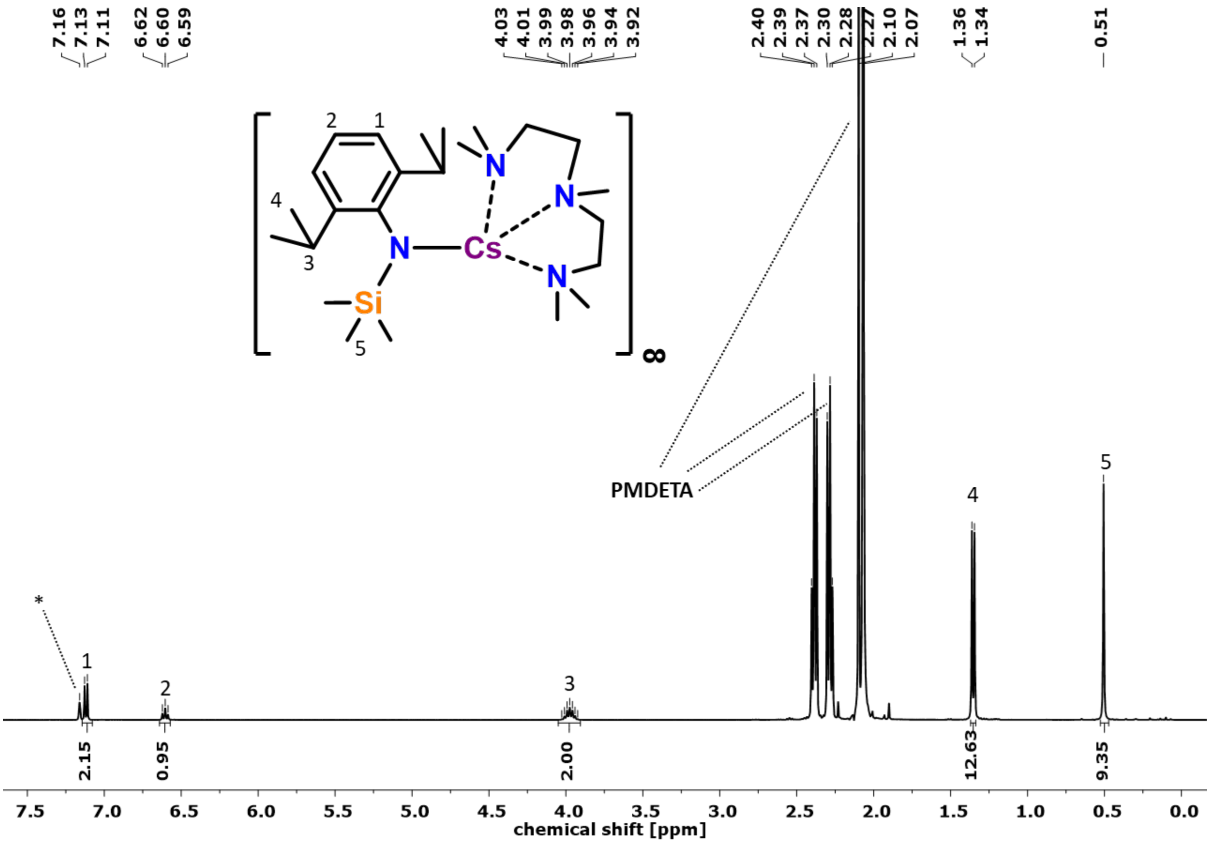

Figure S54. ^{1}H NMR spectrum of Cs{N(SiMe$_3$)Dipp}(PMDETA) in benzene-d_6 (*).

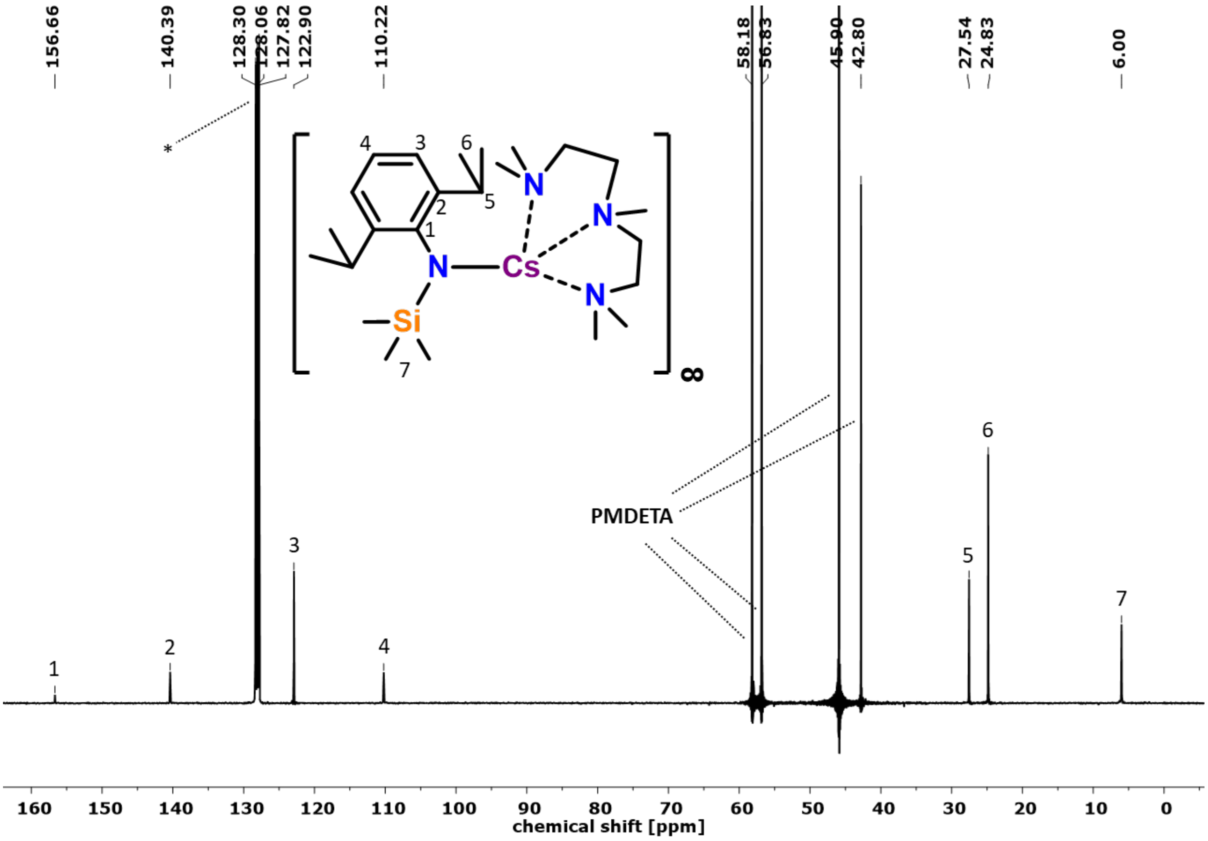

Figure S55. ^{13}C NMR spectrum of Cs{N(SiMe$_3$)Dipp}(PMDETA) in benzene-*d$_6$* (*).

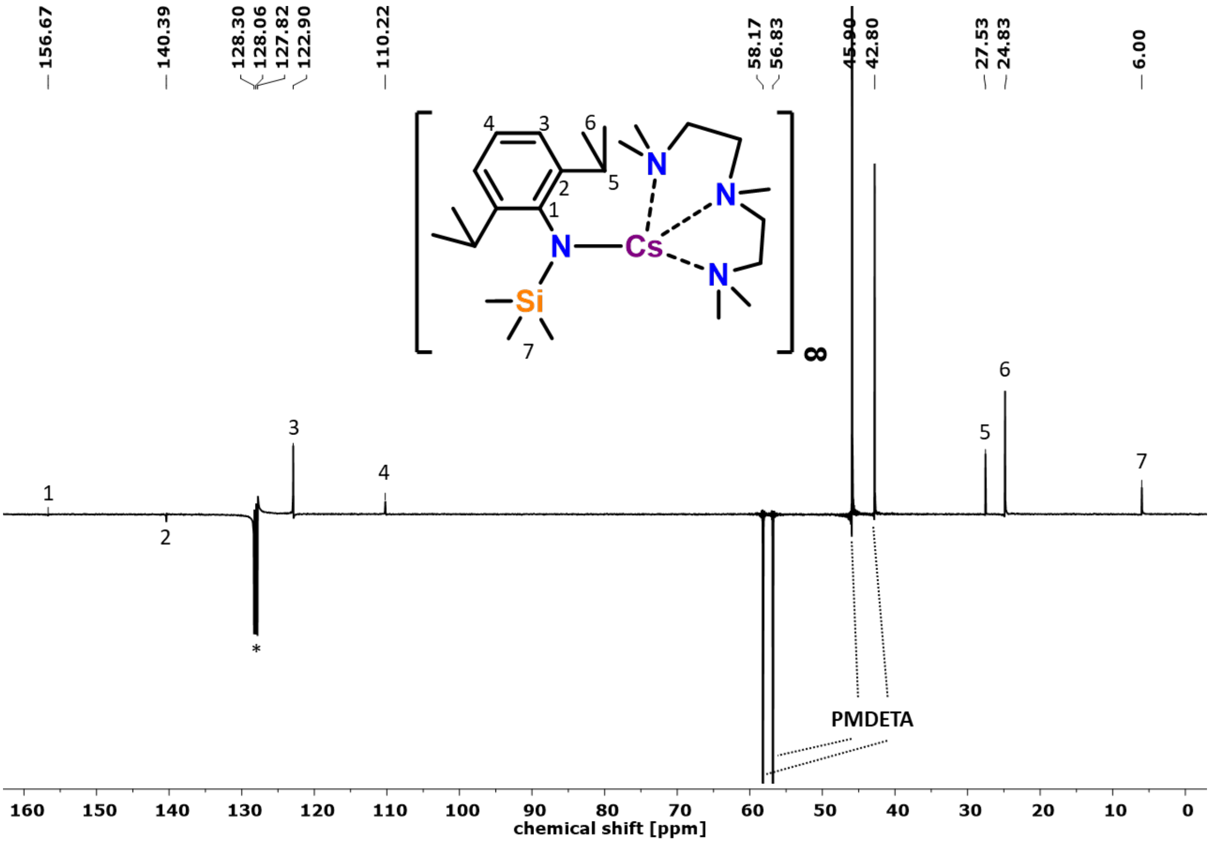

Figure S56. ^{13}C-DEPT-135 NMR spectrum of Cs{N(SiMe$_3$)Dipp}(PMDETA) in benzene-*d$_6$* (*).

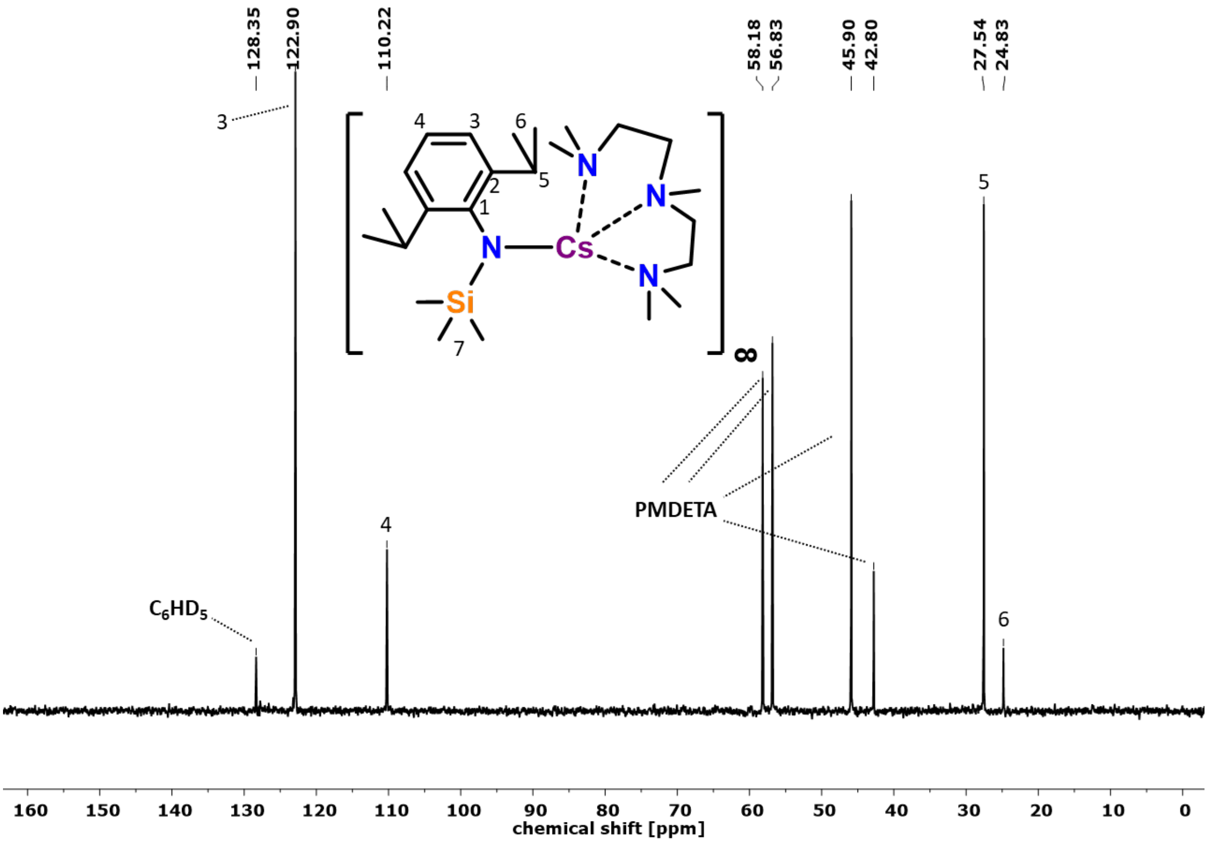

Figure S57. ^{13}C-DEPT-90 NMR spectrum of Cs{N(SiMe$_3$)Dipp}(PMDETA) in benzene-d_6.

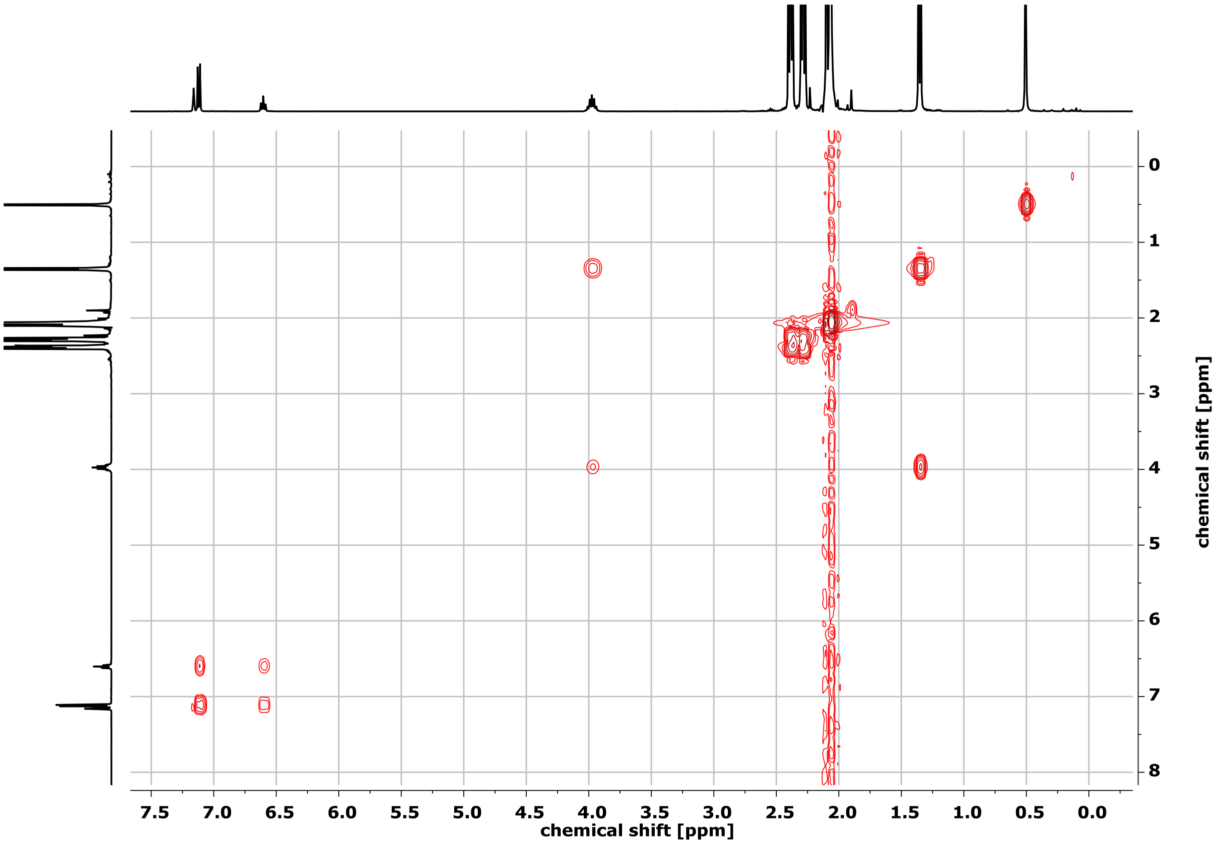

Figure S58. ^{1}H^{1}H-COSY NMR spectrum of Cs{N(SiMe$_3$)Dipp}(PMDETA) in benzene-d_6.

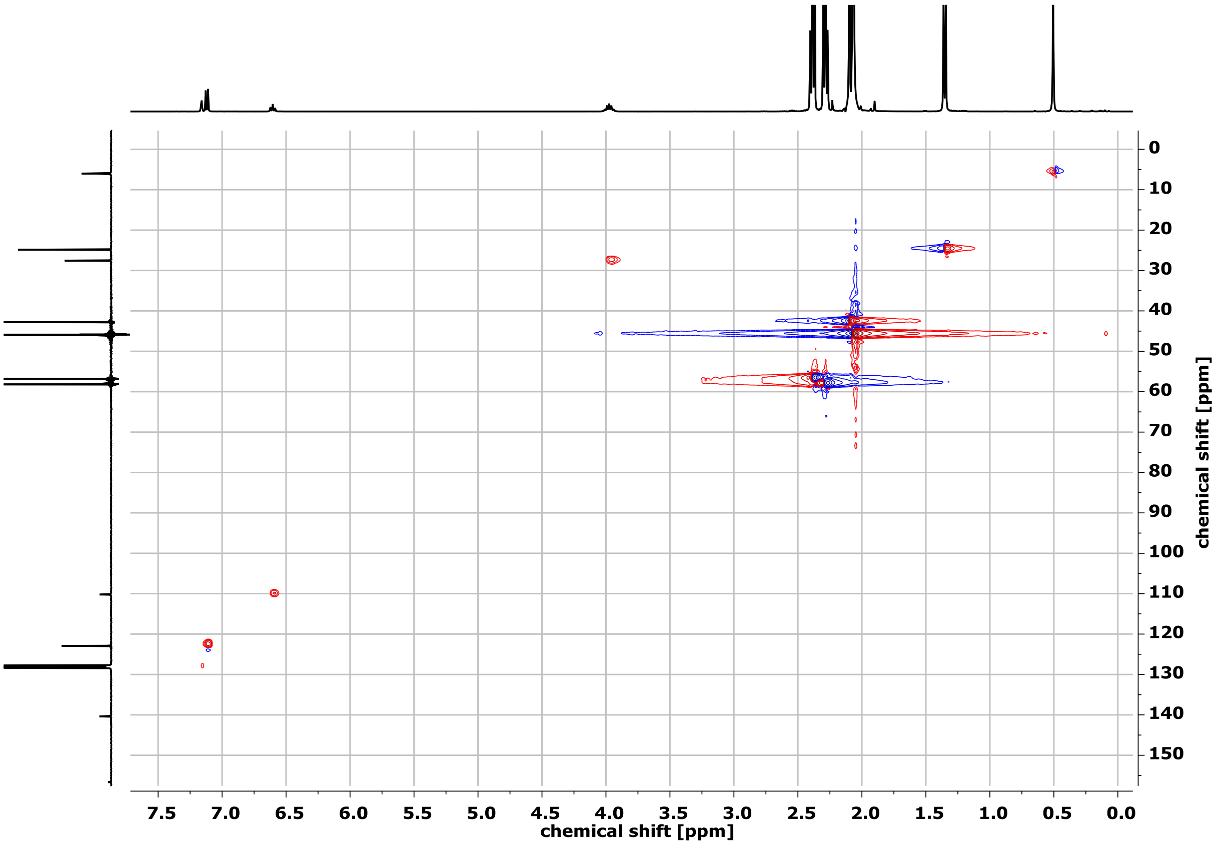

Figure S59. $^{1}H^{13}C$-HSQC NMR spectrum of Cs{N(SiMe$_3$)Dipp}(PMDETA) in benzene-*d$_6$*.

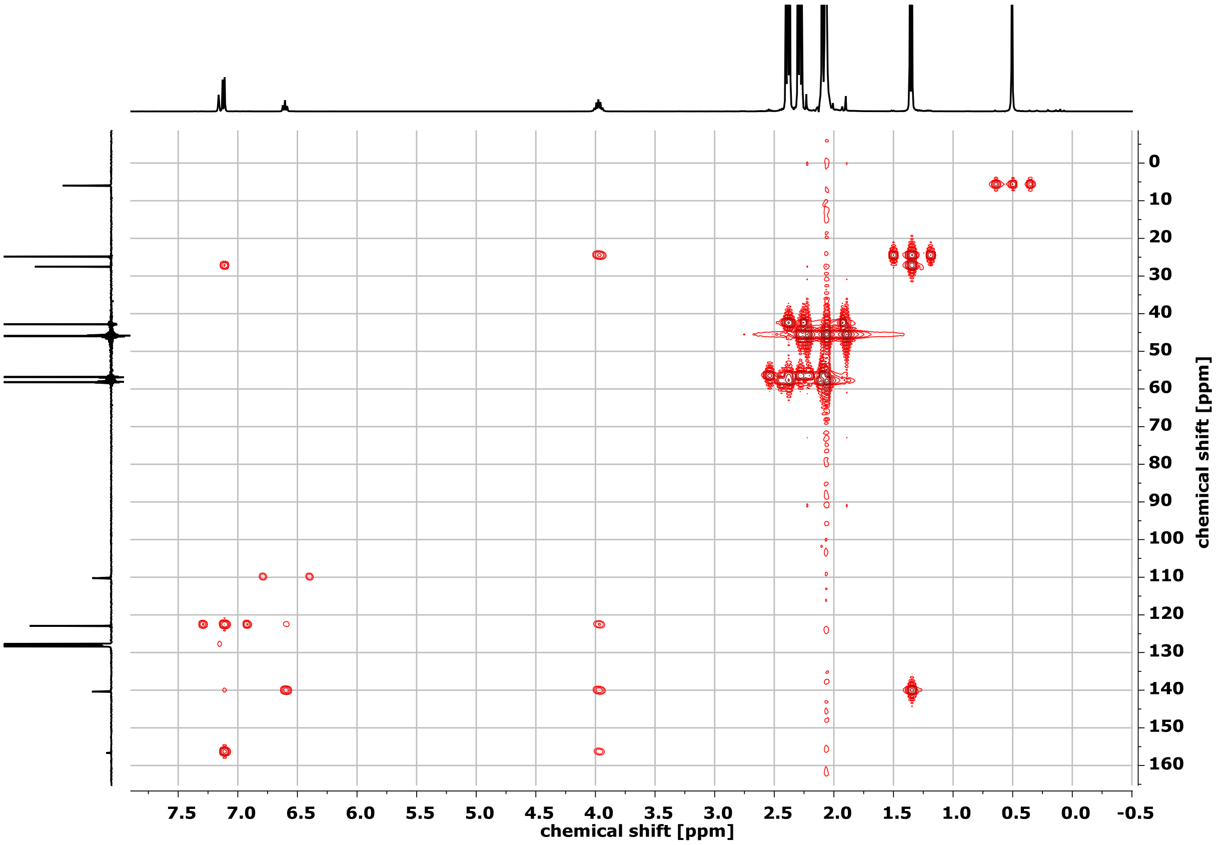

Figure S60. $^{1}H^{13}C$-HMBC NMR spectrum of Cs{N(SiMe$_3$)Dipp}(PMDETA) in benzene-*d$_6$*.

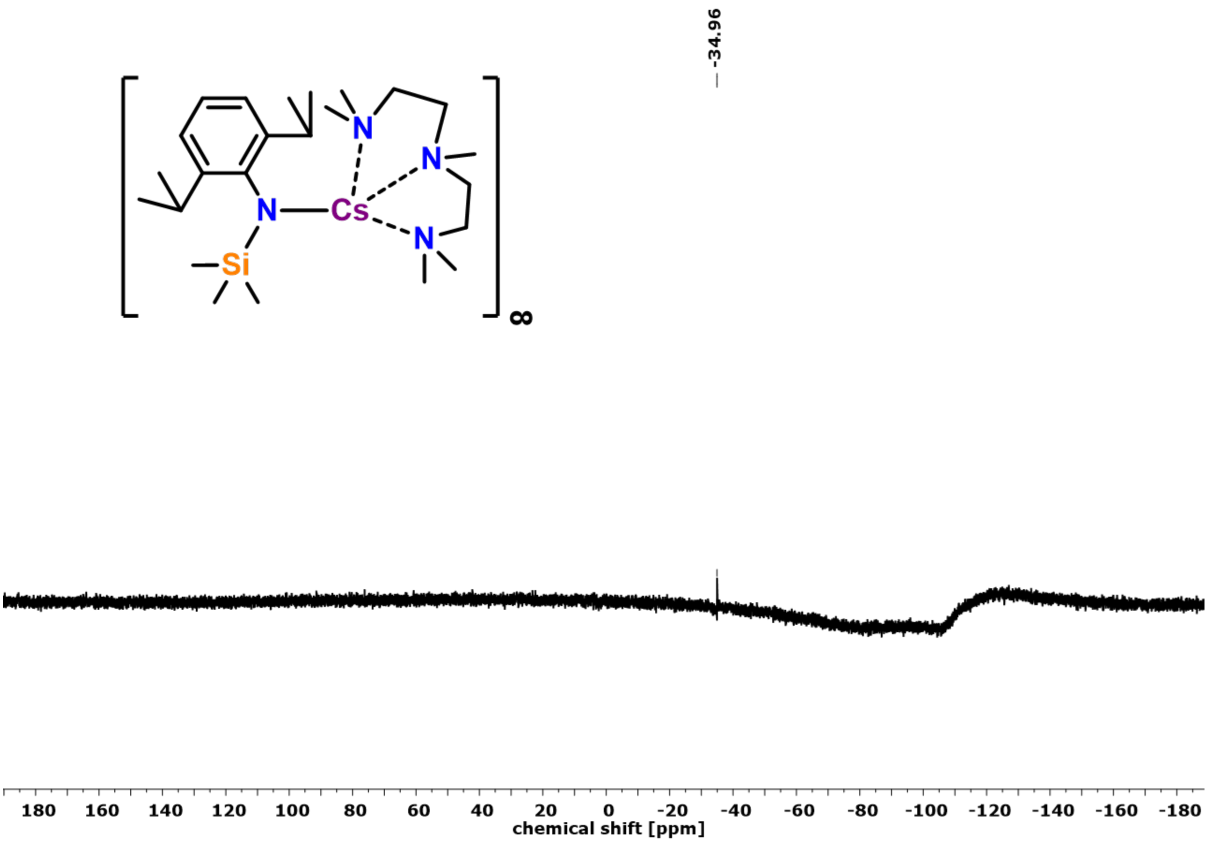

Figure S61. ^{29}Si NMR spectrum of Cs{N(SiMe$_3$)Dipp}(PMDETA) in benzene-d_6.

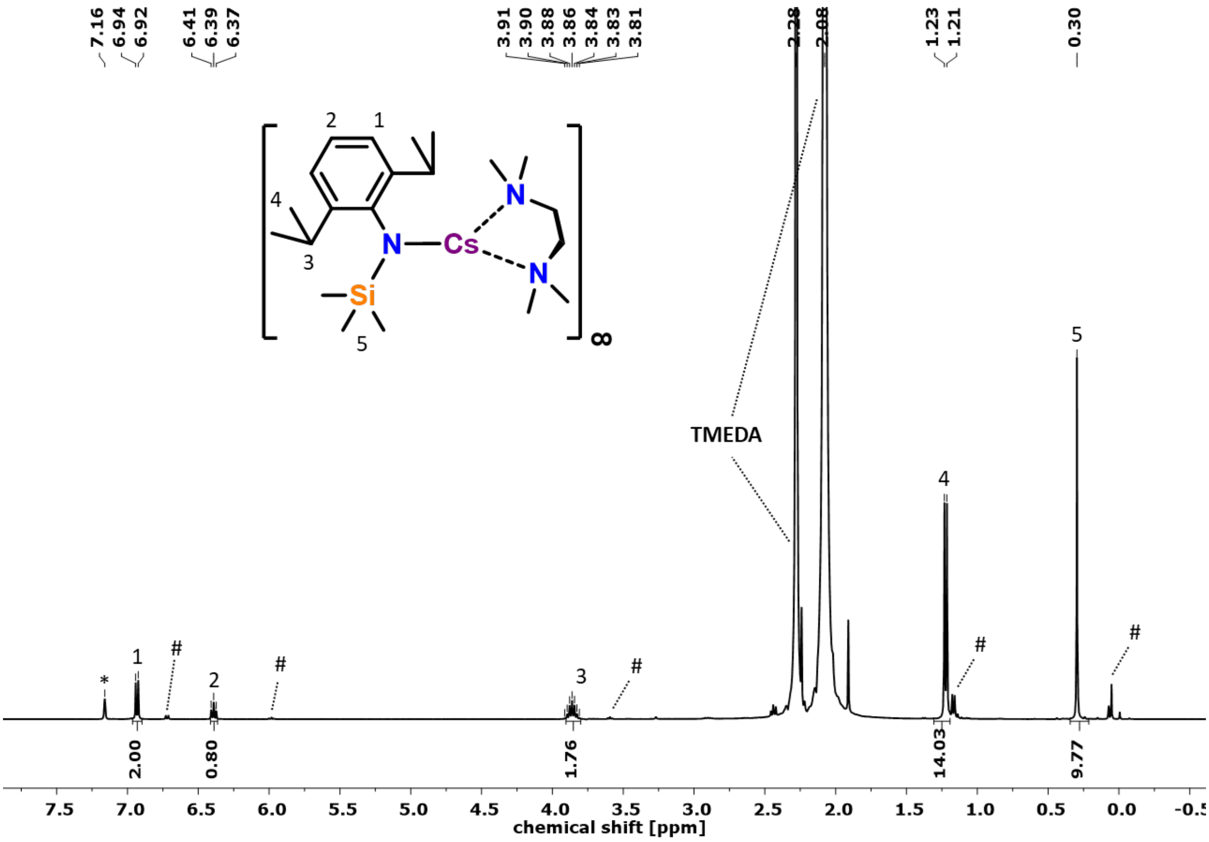

Figure S62. ^{1}H NMR spectrum of Cs{N(SiMe$_3$)Dipp}(TMEDA) in benzene-d_6 (*). An excess of TMEDA was used to fully dissolve sample and an unknown amide impurity (#) is observed.

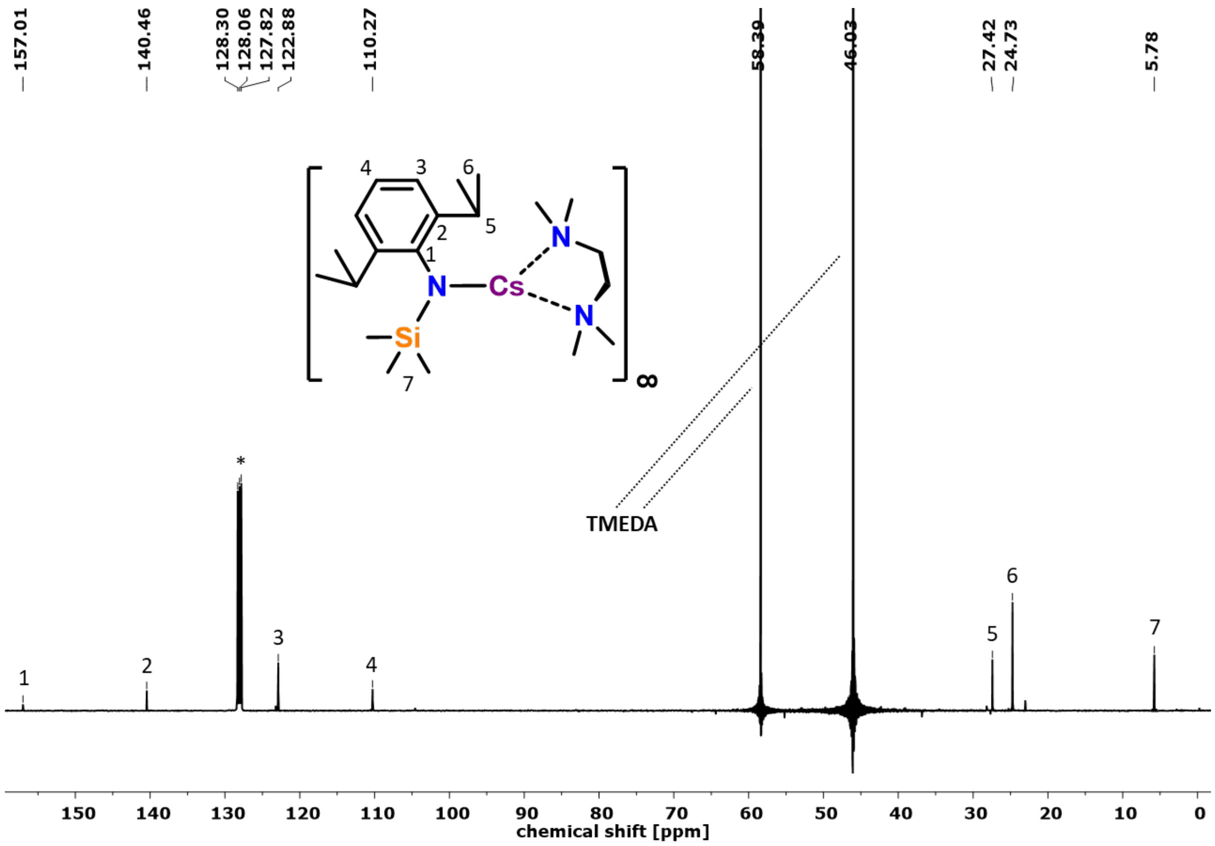

Figure S63. ^{13}C NMR spectrum of Cs{N($SiMe_3$)Dipp}(TMEDA) in benzene-d_6 (*). An excess of TMEDA was used to fully dissolve sample.

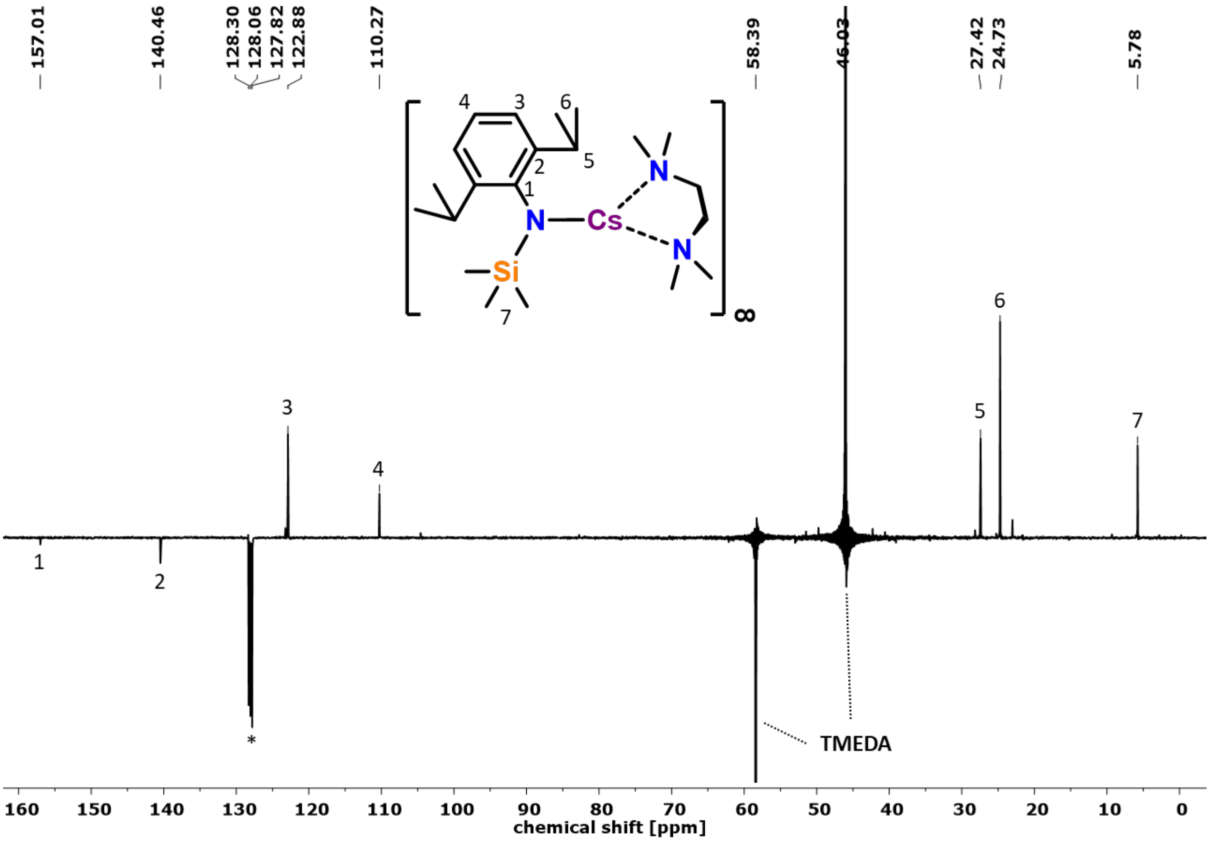

Figure S64. ^{13}C-DEPT-135 NMR spectrum of Cs{N($SiMe_3$)Dipp}(TMEDA) in benzene-d_6 (*). An excess of TMEDA was used to fully dissolve sample.

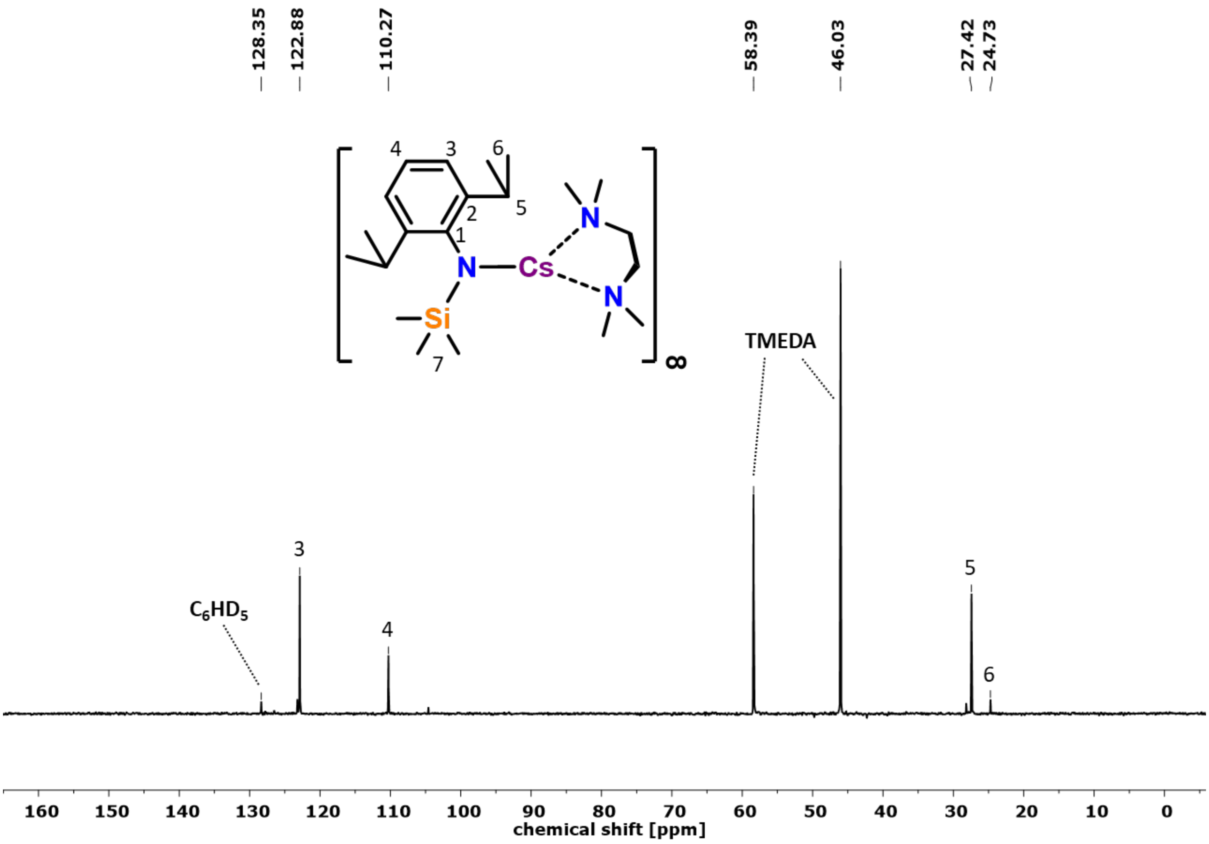

Figure S65. ^{13}C-DEPT-90 NMR spectrum of Cs{N(SiMe$_3$)Dipp}(TMEDA) in benzene-d_6. An excess of TMEDA was used to fully dissolve sample.

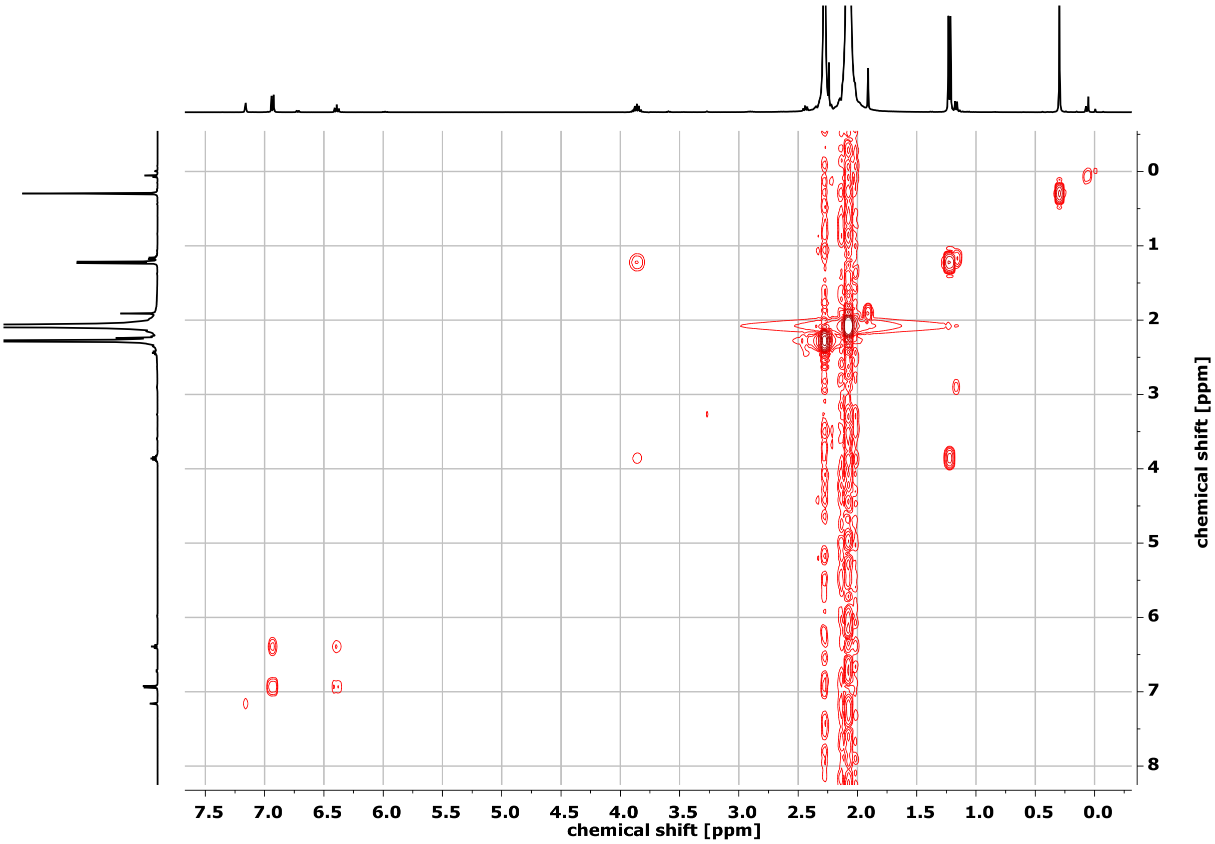

Figure S66. ^{1}H^{1}H-COSY NMR spectrum of Cs{N(SiMe$_3$)Dipp}(TMEDA) in benzene-d_6. An excess of TMEDA was used to fully dissolve sample.

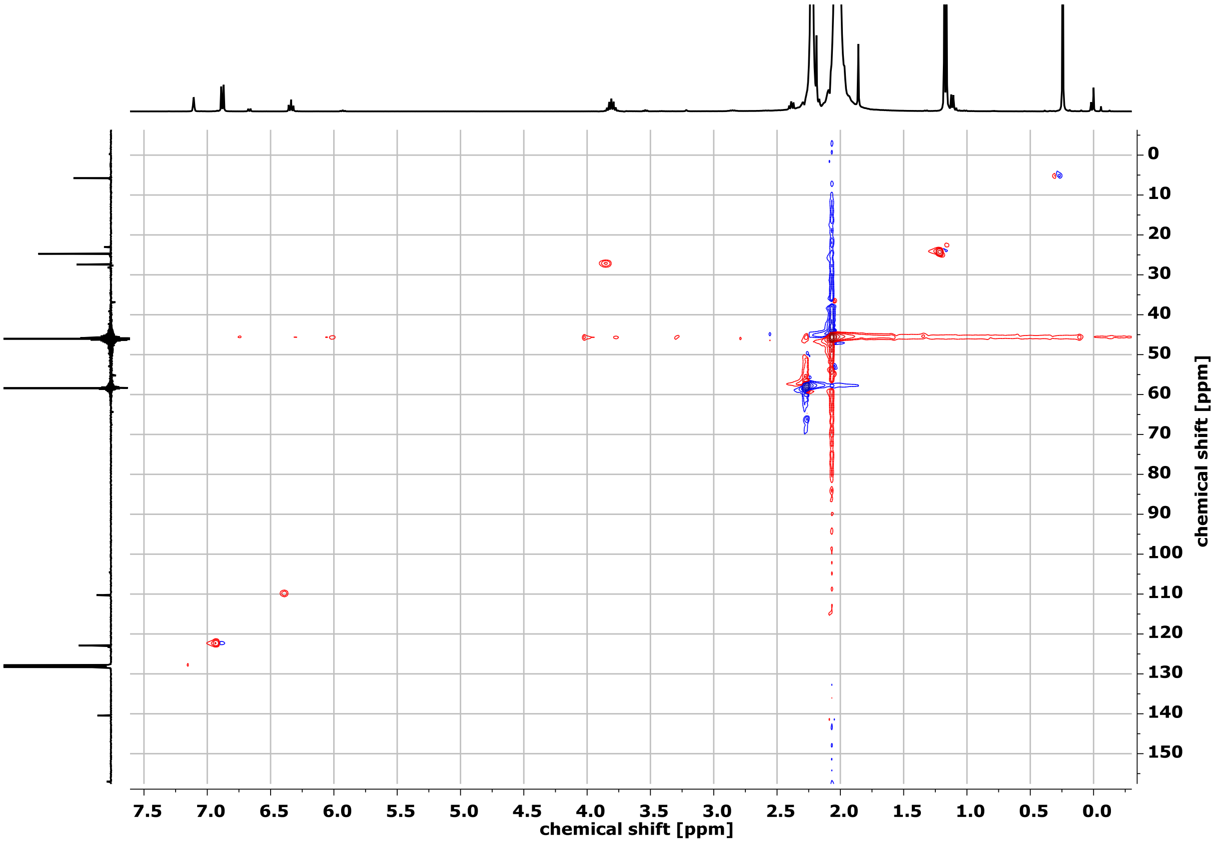

Figure S67. $^{1}H^{13}C$-HSQC NMR spectrum of Cs{N(SiMe$_3$)Dipp}(TMEDA) in benzene-*d*$_6$. An excess of TMEDA was used to fully dissolve sample.

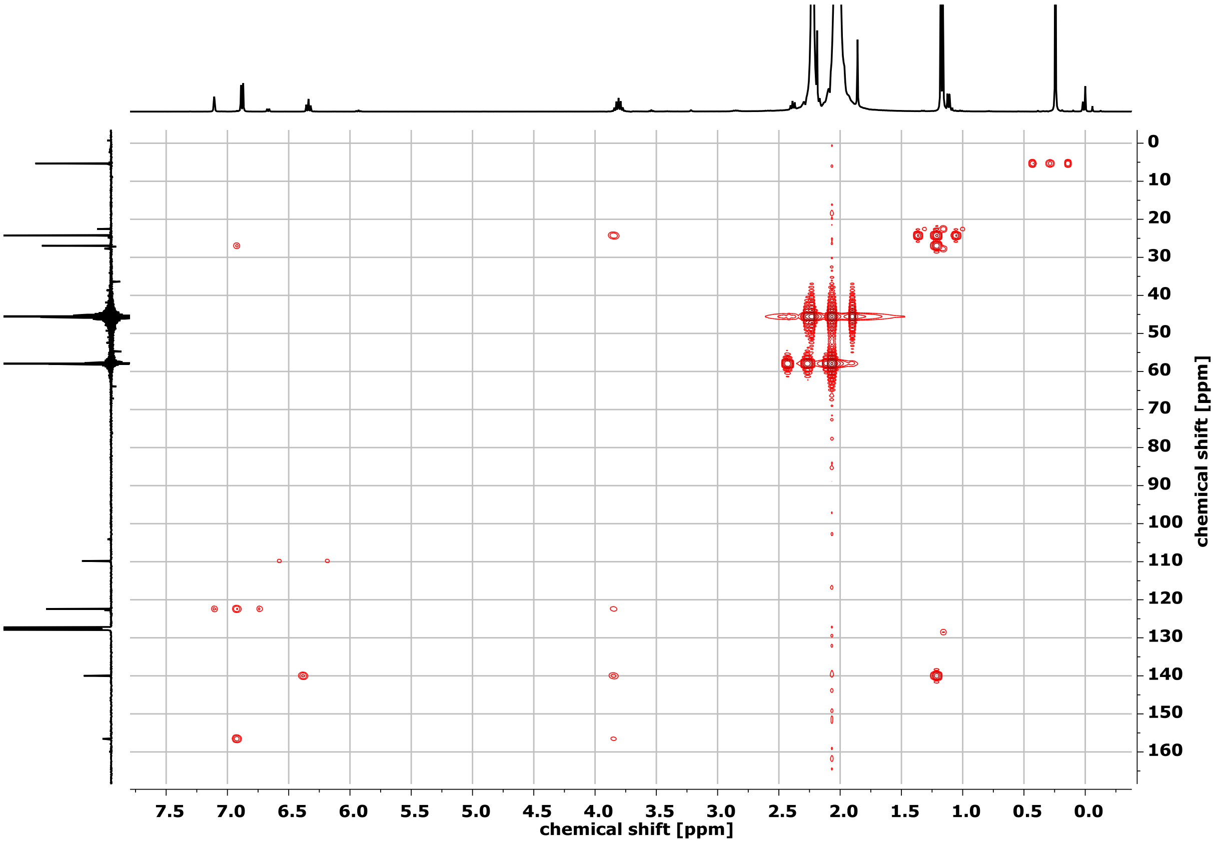

Figure S68. $^{1}H^{13}C$-HMBC NMR spectrum of Cs{N(SiMe$_3$)Dipp}(TMEDA) in benzene-*d*$_6$. An excess of TMEDA was used to fully dissolve sample.

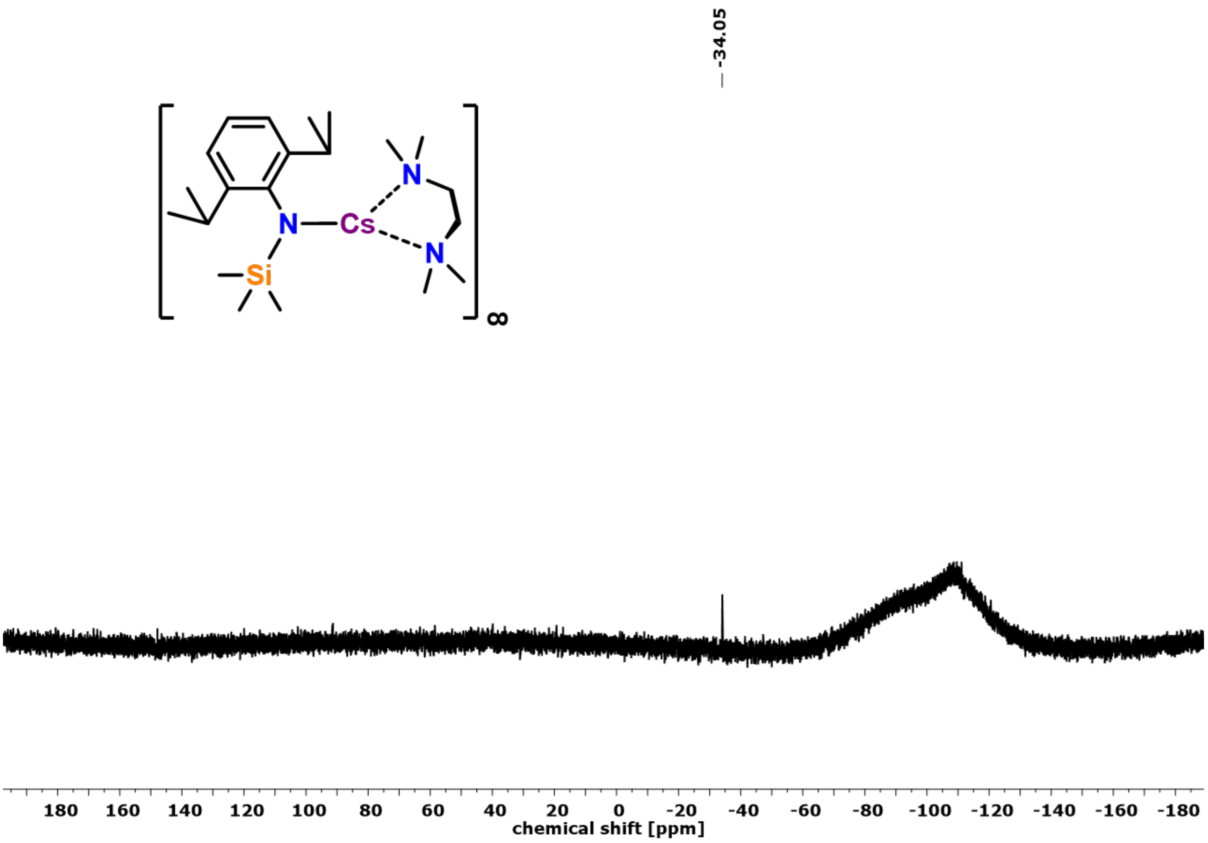

Figure S69. ^{29}Si NMR spectrum of Cs{N(SiMe$_3$)Dipp}(TMEDA) in benzene-d_6. An excess of TMEDA was used to fully dissolve sample.

Evans Method

Solution magnetic susceptibilities were determined by the Evans method at 300 K. The effective magnetic moment μ_{eff} is determined by following equations:

$$\mu_{eff} = \sqrt{4S(S+1)} = \sqrt{n(n+2)} = \sqrt{8(\chi'_M T)}$$

With $\mu_{eff} = \sqrt{8(\chi'_M \mathrm{T})}$ and $\chi'_M = \chi_M - \chi_D$ and $\chi_g = \dfrac{3\Delta f}{4\pi F c}$

While $\chi_M = \chi_g \times MW$ and $\chi_D \approx -\dfrac{MW}{2} \times 10^{-6}$

Δf	Frequency shift [Hz]
F	Spectrometer frequency [Hz]
c	Mass concentration [g cm^{-3}]
X_g	Mass susceptibility [cm^3 g^{-1}]
MW	Molar mass/molecular weight [g mol^{-1}]
X_M	Molar susceptibility [cm^3 mol^{-1}]
X_D	Diamagnetic correction [cm^3]
X'_M	Corrected molar susceptibility [cm^3 mol^{-1}]
T	Measurement temperature [K]
μ_{eff}	Effective magnetic moment [μ_B]

Determination of frequency shift in ^{1}H-NMR spectrum (recorded in THF-*d8*):

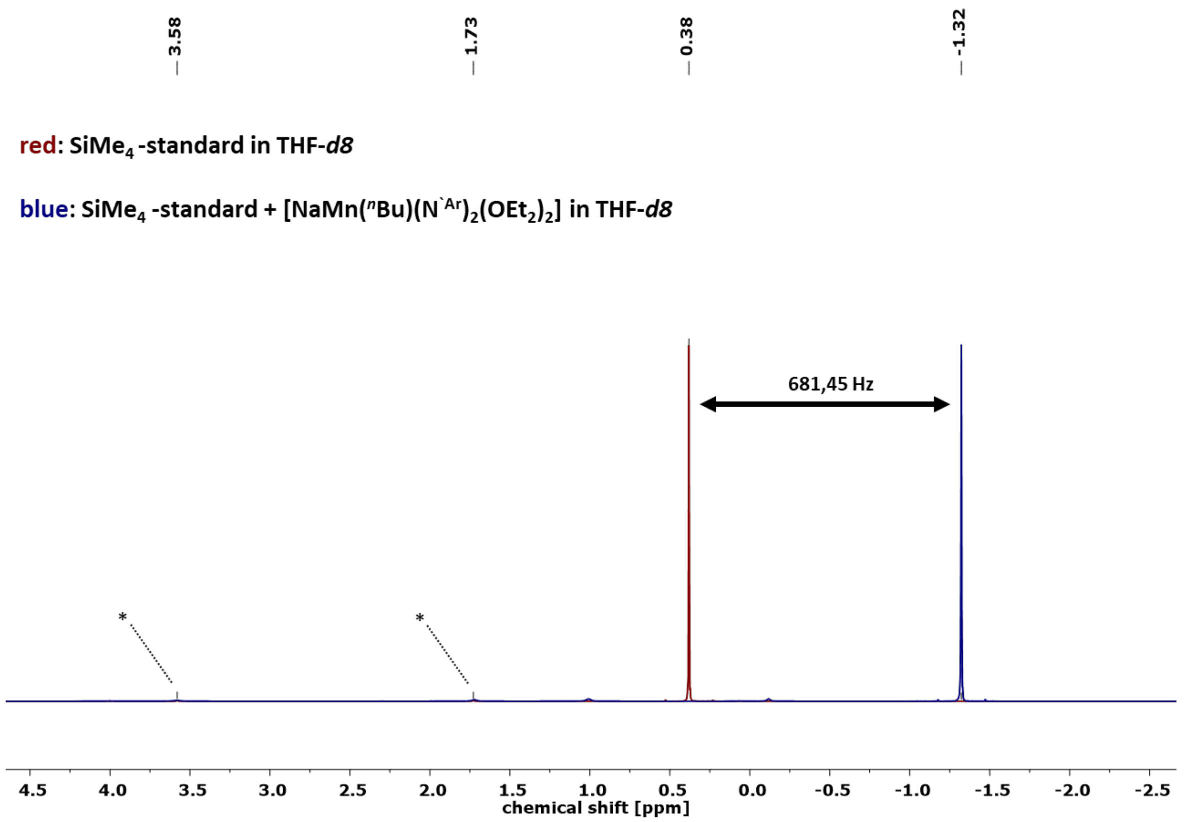

Figure S70. Stacked ^{1}H NMR spectra of $SiMe_4$ standard (red) and shifted standard signal due to paramagnetic compound [NaMn(^{n}Bu)($N^{`Ar}$)$_2$(OEt_2)$_2$] in THF-d_8 (blue).

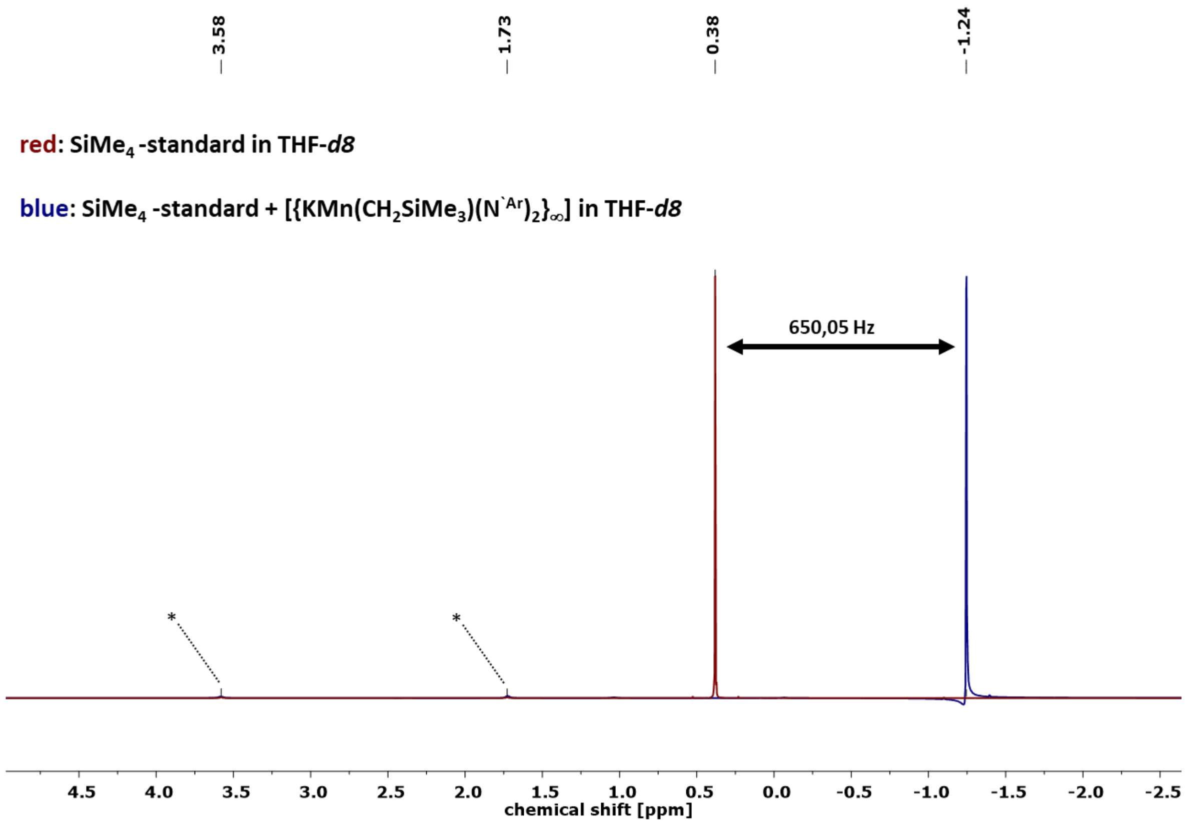

Figure S71. Stacked ^{1}H NMR spectra of $SiMe_4$ standard (red) and shifted standard signal due to paramagnetic compound [{KMn(CH_2SiMe_3)($N^{`Ar}$)$_2$}$_\infty$] in THF-d_8 (blue).

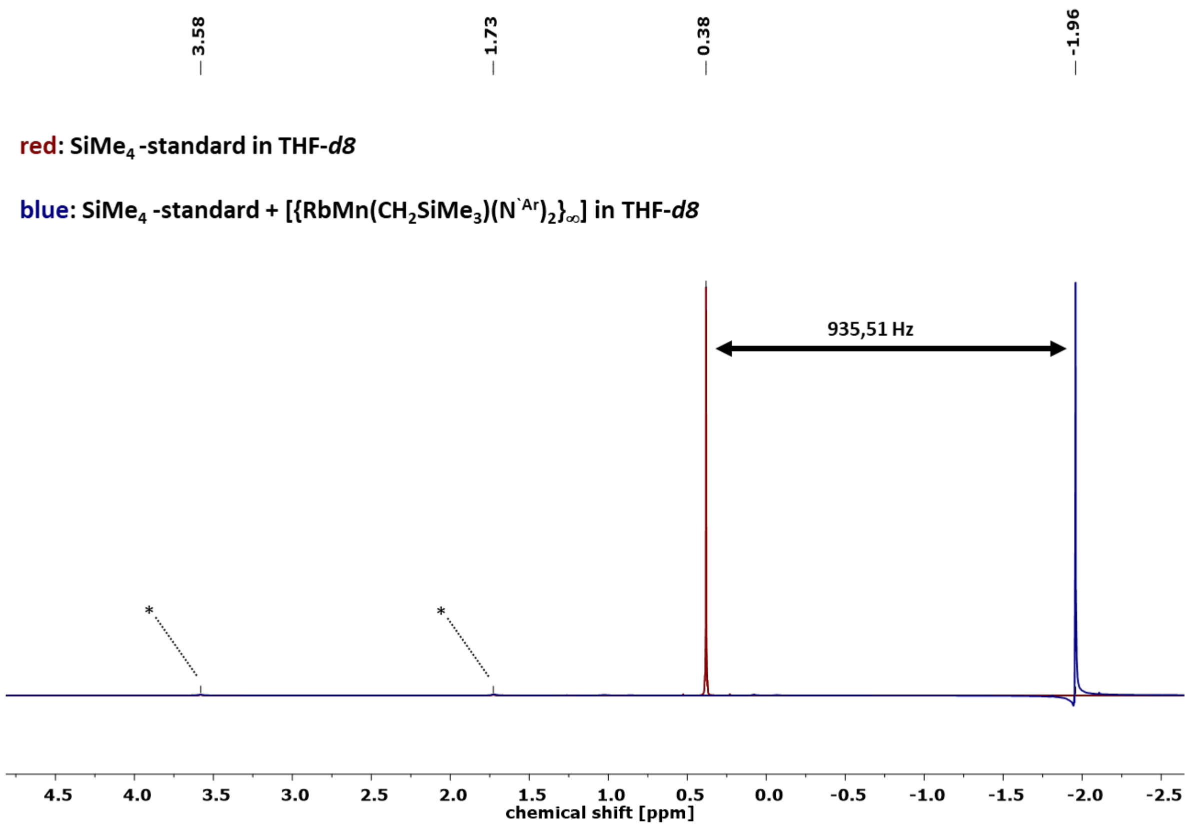

Figure S72. Stacked ^{1}H NMR spectra of $SiMe_4$ standard (red) and shifted standard signal due to paramagnetic compound [{$RbMn(CH_2SiMe_3)(N^{`Ar})_2$}$_\infty$] in THF-$d_8$ (blue).

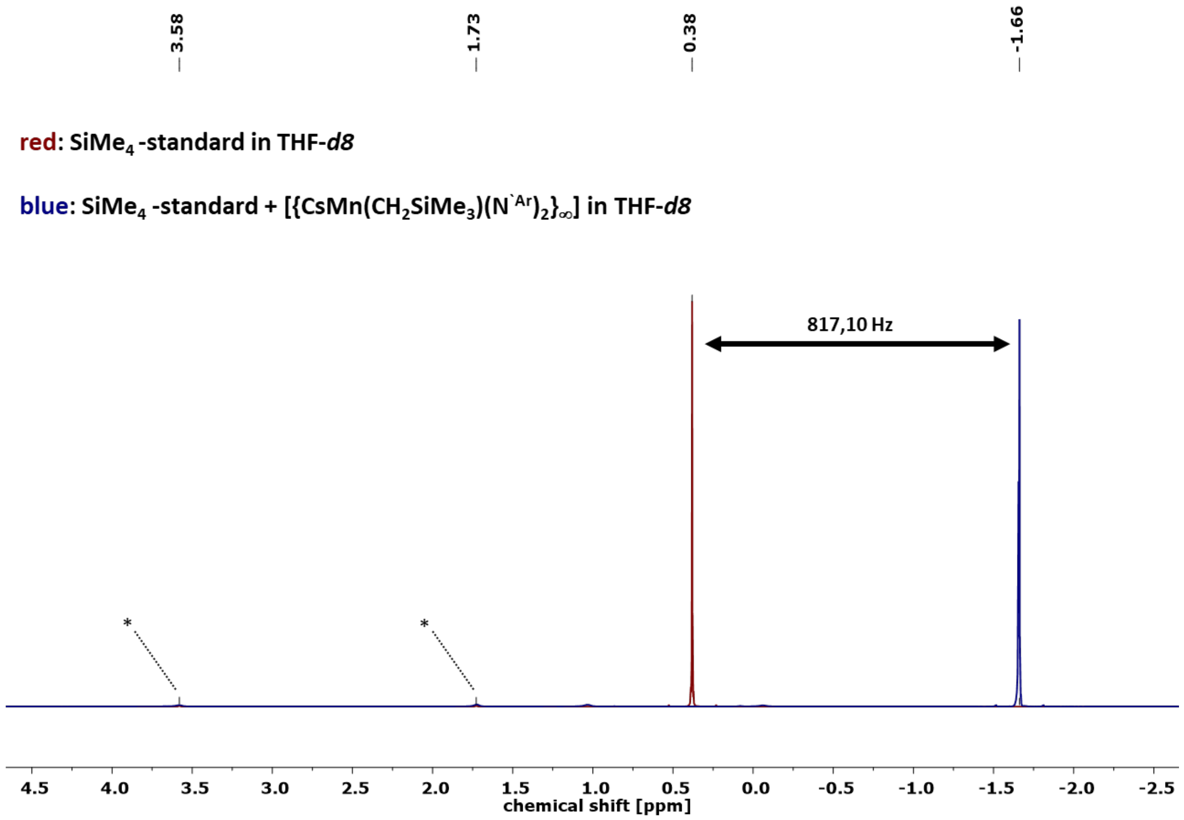

Figure S73. Stacked ^{1}H NMR spectra of $SiMe_4$ standard (red) and shifted standard signal due to paramagnetic compound [{$CsMn(CH_2SiMe_3)(N^{`Ar})_2$}$_\infty$] in THF-$d_8$ (blue).

Table S12. Determination of effective magnetic moment μ_B via Evans Method.

compound	c<br>mass concentration of sample	χ_g<br>Measured mass susceptibility	M_r<br>Molar mass of sample	χ_m<br>Measured molar susceptibility	χ_D<br>Diamagnetic correction	χ'_m<br>Corrected molar susceptibility	μ_{eff}<br>Effective magnetic moment
	**g cm^{-3}**	**cm^3 g^{-1}**	**g mol^{-1}**	**cm^3 mol^{-1}**	**cm^3 mol^{-1}**	**cm^3 mol^{-1}**	**μ_B**
**[NaMn(^{n}Bu)(N$^{`Ar}$)$_2$(OEt$_2$)$_2$**	2,0782E-02	1,9569E-05	736,1	1,4405E-02	-3,68E-04	1,4773E-02	**5,94**
**KMn(CH$_2$SiMe$_3$)(N$^{`Ar}$)$_2$**	1,9015E-02	2,0402E-05	678,18	1,3836E-02	-3,39E-04	1,4175E-02	**5,81**
**RbMn(CH$_2$SiMe$_3$)(N$^{`Ar}$)$_2$**	2,6355E-02	2,1184E-05	724,55	1,5349E-02	-3,62E-04	1,5711E-02	**6,12**
**CsMn(CH$_2$SiMe$_3$)(N$^{`Ar}$)$_2$**	2,8579E-02	1,7063E-05	771,99	1,3172E-02	-3,86E-04	1,3558E-02	**5,69**

4) Selected infrared spectra

Infrared vibrational spectra, shown in Figures S74 –S78, were obtained as Nujol mulls on NaCl plates. Mulls were prepared in the glove box using anhydrous Nujol, which was dried and stored over activated 4 Å molecular sieves under argon, and then transferred to the spectrometer in a desiccator. Spectra were recorded on a Nicolet 360 FTIR spectrometer spanning 4000-400 cm^{-1} at room temperature.

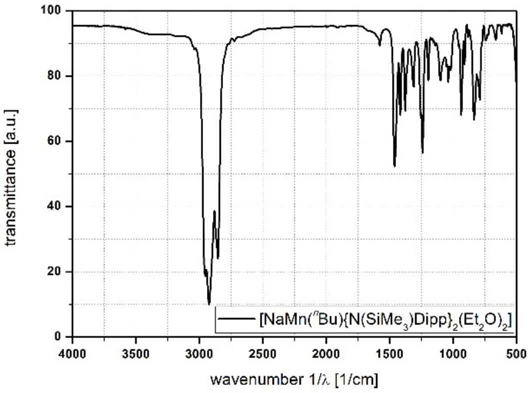

Figure S74. IR vibrational spectrum of [NaMn(^{n}Bu){N(SiMe$_3$)Dipp}$_2$(Et$_2$O)$_2$] (**1**).

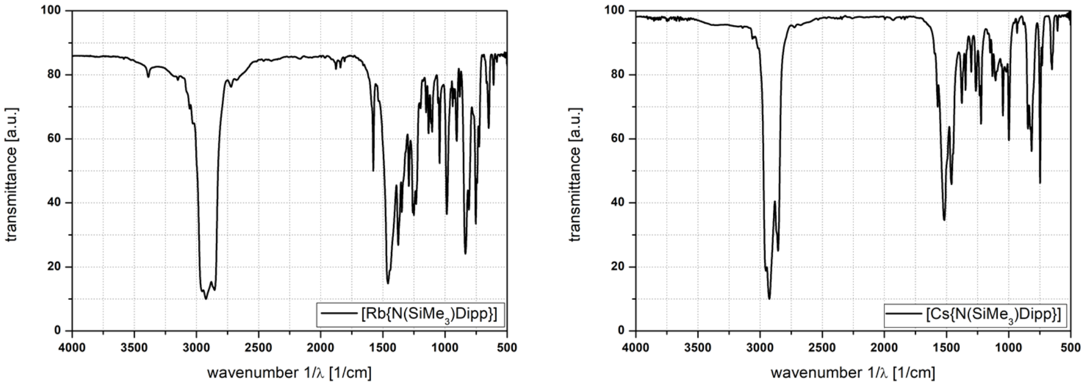

Figure S75. IR vibrational spectrum of [Rb{N(SiMe$_3$)Dipp}] (**3-Rb**) (left) and [Cs{N(SiMe$_3$)Dipp}] (**3-Cs**) (right).

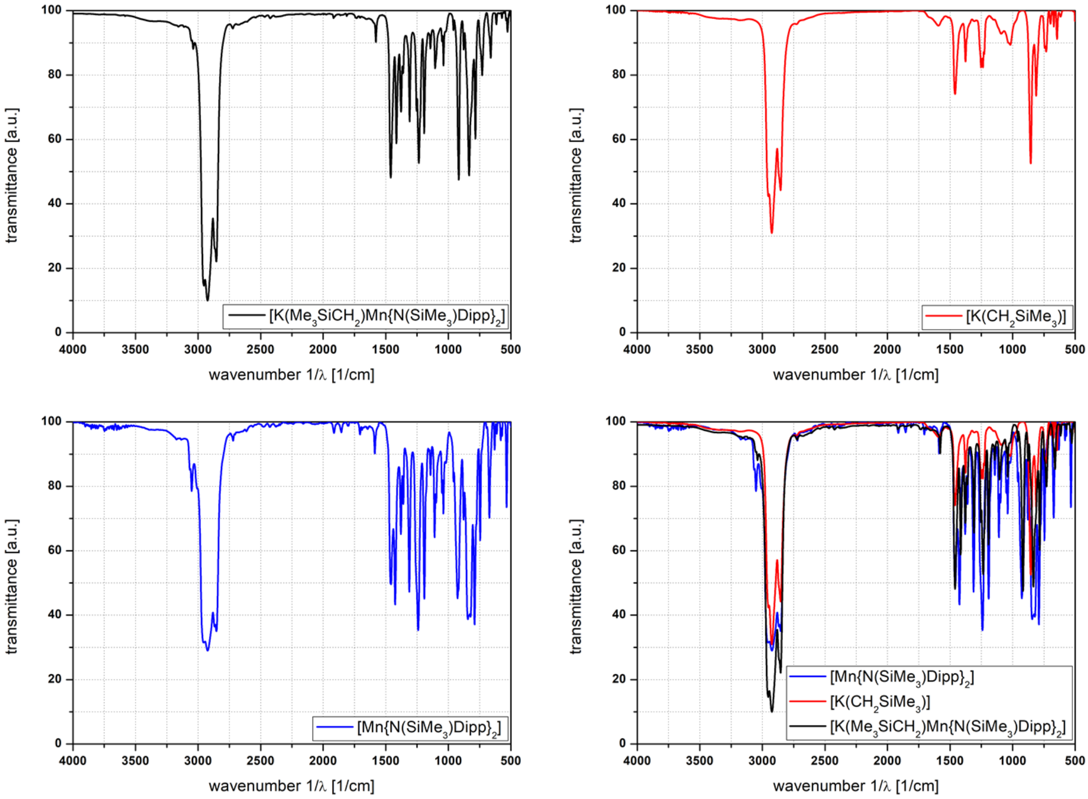

Figure S76. IR vibrational spectrum of [K(Me$_3$SiCH$_2$)Mn{N(SiMe$_3$)Dipp}$_2$] (**4-K**) (top left), K(CH$_2$SiMe$_3$) (top right), (Mn{N(SiMe$_3$)Dipp}$_2$ (bottom left), and superposition of all three spectra (bottom right).

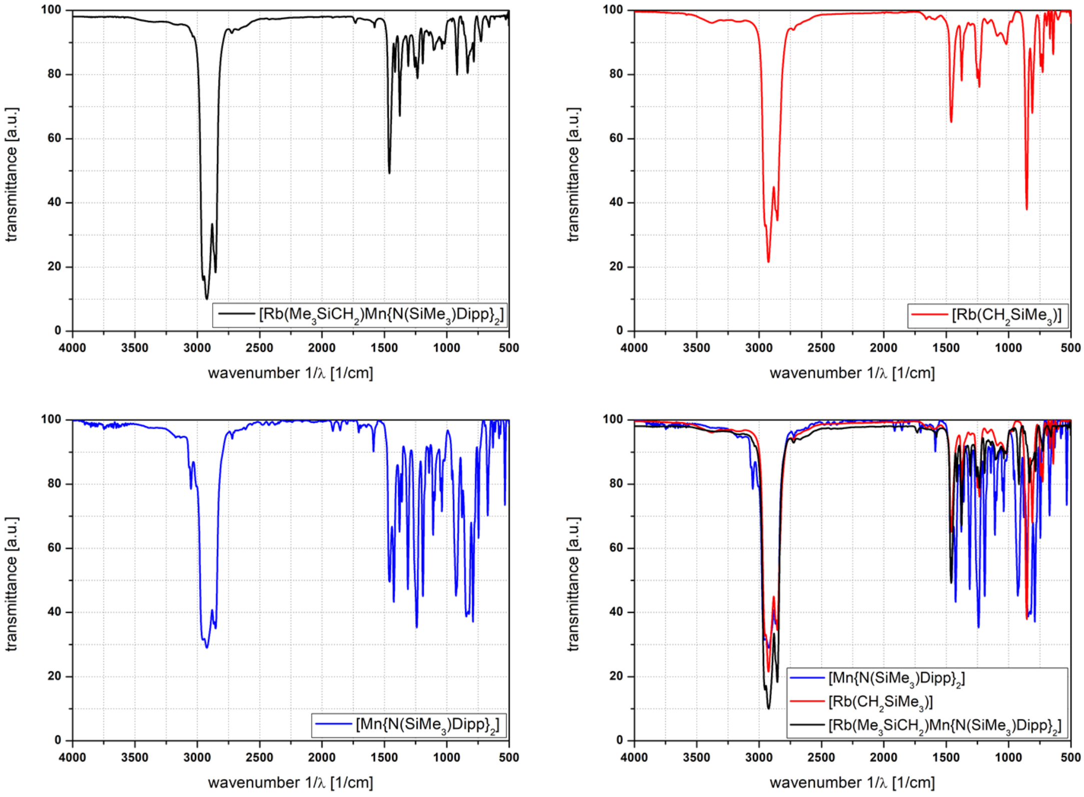

Figure S77. IR vibrational spectrum of [Rb(Me_3SiCH_2)Mn{N($SiMe_3$)Dipp}$_2$] (**4-Rb**) (top left), Rb(CH_2SiMe_3) (top right), (Mn{N($SiMe_3$)Dipp}$_2$ (bottom left), and superposition of all three spectra (bottom right).

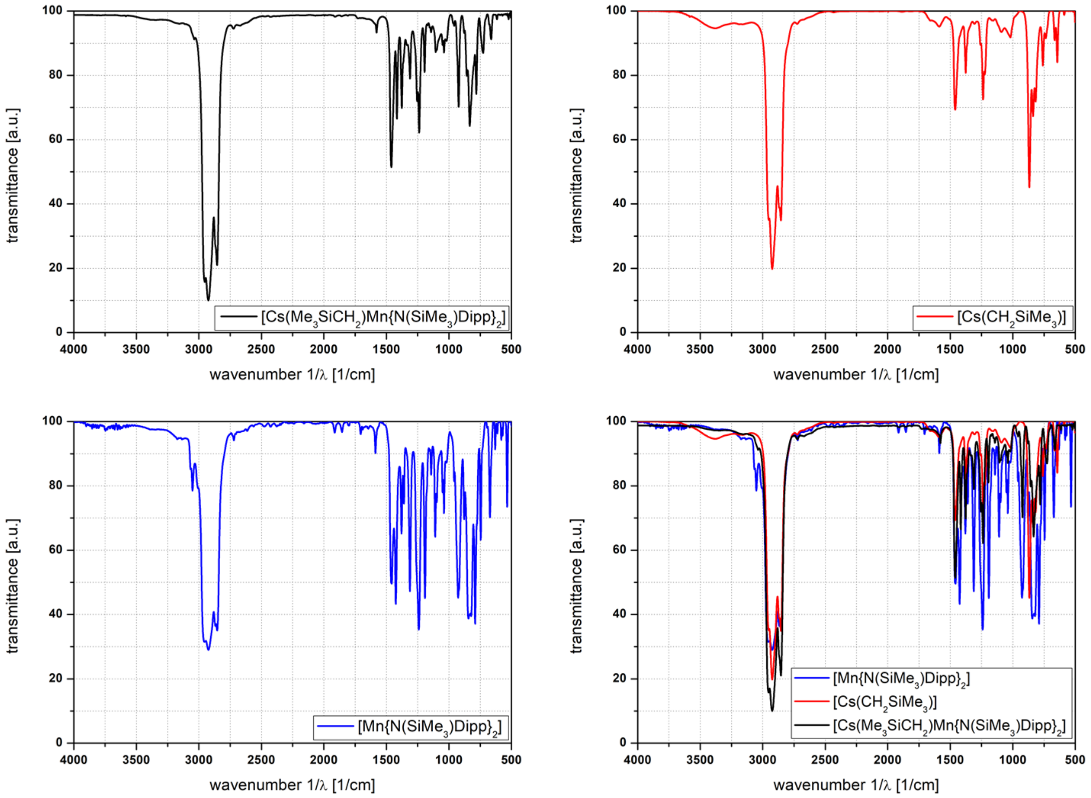

Figure S78. IR vibrational spectrum of [Cs(Me_3SiCH_2)Mn{N($SiMe_3$)Dipp}$_2$] (**4-Cs**) (top left), (CH_2SiMe_3) (top right), (Mn{N($SiMe_3$)Dipp}$_2$ (bottom left), and superposition of all three spectra (bottom right).

5) References

[1] Y. W. Chao, P. A. Wexler, D. E. Wigley, *Inorg. Chem.* **1989**, *28*, 3860–3868.

[2] D. K. Kennepohl, S. Brooker, G. M. Sheldrick, H. W. Roesky, *Chem. Ber.* **1991**, *124*, 2223–2225.

[3] C. Schade, W. Bauer, P. von Ragué Schleyer, *J. Organomet. Chem.* **1985**, *295*, 25–28.

[4] W. Clegg, B. Conway, A. R. Kennedy, J. Klett, R. E. Mulvey, L. Russo, *Eur. J. Inorg. Chem.* **2011**, 721–726.

[5] B. Conway, D. v. Graham, E. Hevia, A. R. Kennedy, J. Klett, R. E. Mulvey, *Chem. Commun.* **2008**, 2638–2640.

[6] S. Neander, U. Behrens, *Z. Anorg. Allg. Chem.* **1999**, *625*, 1429 – 1434.

[7] A. I. Ojeda-Amador, A. J. Martínez-Martínez, A. R. Kennedy, C. T. O'Hara, *Inorg. Chem.* **2016**, *55*, 5719–5728.

[8] W. Clegg, A. R. Kennedy, J. Klett, R. E. Mulvey, L. Russo, *Eur. J. Inorg. Chem.* **2012**, 2989–2994.

[9] M. Kaiser, J. Klett, *Dalton Trans.* **2018**, *47*, 12582–12586.

[10] D. F. Evans, *J. Chem. Soc.* **1959**, *81*, 2003–2005.

[11] E. M. Schubert, *J. Chem. Educ.* **1992**, *69*, 62.

[12] C. Piguet, *J. Chem. Educ.* **1997**, *74*, 815.

[13] 2018, CrysAlisPro Software system, version 1.171.39.46, Rigaku Corporation, Oxford, UK Rigaku Oxford DiffractionRigaku Oxford Diffraction, **2018**.

[14] O. v. Dolomanov, L. J. Bourhis, R. J. Gildea, J. A. K. Howard, H. Puschmann, *J. Appl. Cryst.* **2009**, *42*, 339–341.

[15] G. M. Sheldrick, *Acta Cryst.* **2015**, C*71*, 3–8.

[16] G. M. Sheldrick, *Acta Cryst.* **2008**, A*64*, 112–122.

[17] L. J. Farrugia, *J. Appl. Cryst.* **2012**, *45*, 849–854.

[18] D. K. Kennepohl, S. Brooker, G. M. Sheldrick, H. W. Roesky, *Z. Naturforsch. B* **1992**, *47*, 9–16.

[19] C. G. Werncke, E. Suturina, P. C. Bunting, L. Vendier, J. R. Long, M. Atanasov, F. Neese, S. Sabo-Etienne, S. Bontemps, *Chem. Eur. J.* **2016**, *22*, 1668–1674.
